# Sweet Potato Gene Clusters Control Anthocyanin Biosynthesis and Leaf Morphology

**DOI:** 10.1111/pbi.70636

**Published:** 2026-03-24

**Authors:** Dong An, Tiechen Shen, Shiyu Wu, Yanshen Li, Weijuan Fan, Mengxiao Yan, Yinghui Meng, Xinyi Wang, Ximing Xu, Zunfu Lv, Ling Yuan, Jun Yang, Guoquan Lu, Hongxia Wang

**Affiliations:** ^1^ Shanghai Key Laboratory of Plant Functional Genomics and Resources Shanghai Chenshan Botanical Garden Shanghai China; ^2^ College of Advanced Agricultural Sciences, Zhejiang A&F University Hangzhou Zhejiang China; ^3^ CAS Center for Excellence in Molecular Plant Sciences Shanghai Institute of Plant Physiology & Ecology, Chinese Academy of Sciences Shanghai China; ^4^ University of Chinese Academy of Sciences Beijing China; ^5^ Department of Plant and Soil Sciences, and Kentucky Tobacco Research and Development Center University of Kentucky Lexington Kentucky USA

**Keywords:** anthocyanin, GWAS, leaf morphology, leaf pigmentation, sweet potato, tuberous root pigmentation

## Abstract

Sweet potato ( *Ipomoea batatas* ) exhibits diversity in pigmentation and leaf morphology, yet the genetic architecture and regulatory organisation underlying these traits remain poorly resolved, particularly with respect to organ‐specific control. We hypothesised that phenotypic variation is governed by clustered genetic modules comprising regulatory and structural genes operating in an organ‐specific manner. To test this, we conducted genome‐wide association studies (GWAS) using 4.6 million SNPs across 260 diverse accessions, integrated with transcriptomic, haplotype and functional analyses. GWAS identified two tandem clusters of MYB transcription factors on chromosome 5 as the primary regulators of leaf anthocyanin accumulation. Expression profiling, heterologous expression and transcriptional activation assays demonstrated that *IbMYB2* and *IbMYB3* function as key transcriptional activators and form a mutually reinforcing regulatory module. In contrast, pigmentation in storage roots was associated with a spatially distinct genomic region enriched in anthocyanin biosynthetic genes, including *IbAOMT*, *Ib3GGT* and *IbLDOX*, indicating different regulations between aerial and underground organs. Comparative genomic analysis further revealed expansion and conservation of MYB clusters in sweet potato, suggesting evolutionary selection for enhanced transcriptional control. In addition, GWAS uncovered a major locus on chromosome 7 controlling leaf shape variation. Functional analyses demonstrated that conserved developmental regulators, including *BEL1‐like* (*g29974*), *WD40* (*g26165*) and *LMI1‐like* (*g29859*) genes, play causal roles in leaf margin development. CRISPR/Cas9‐mediated knockout of *g26165* directly reduced leaf lobing, confirming its functional importance. These findings reveal clustered regulatory and structural gene modules underlying key agronomic traits and provide insights into the genetic and evolutionary mechanisms driving phenotypic diversification in sweet potato.

## 1 Introduction

Sweet potato [ *Ipomoea batatas* (L.) Lam.] is a globally important crop contributing to food security, bioenergy production and diverse industrial applications due to its resilience and nutritional value, particularly in developing regions (Sapakhova et al. 2023). Its adaptability and rich nutritional profile, including carbohydrates, fibre, β‐carotene, minerals and anthocyanins, further enhance its value (Mano et al. 2007). Several key agronomic traits, leaf pigmentation, tuberous root pigmentation and leaf morphology, not only define the crop's visual diversity but also carry substantial nutritional, economic and ecological significance. Leaf pigmentation influences photosynthetic efficiency and stress responses, while tuberous root pigmentation, particularly anthocyanin accumulation in purple‐fleshed varieties, confers potent antioxidant properties with substantial health benefits. Leaf morphology directly impacts canopy architecture, light interception efficiency, and ultimately, yield potential. Understanding the genetic basis of these complex traits is therefore critical for precision breeding aimed at improving sweet potato productivity and quality.

Dissecting the genetic architecture of these traits in sweet potato presents unique challenges. As a highly heterozygous, auto‐allohexaploid species (2*n* = 6× = 90), sweet potato possesses a large and complex genome (Yang et al. 2017). This genomic complexity, coupled with historical bottlenecks and relatively limited genomic resources compared with major diploid crops, has hindered forward genetics approaches. Nevertheless, abundant phenotypic diversity and natural genetic variation enable the application of genome‐wide association studies (GWAS) and quantitative trait locus (QTL) mapping to identify loci associated pigment production (e.g., anthocyanin and β‐carotene), storage root development, starch content and leaf architecture (Yada et al. 2017; Haque et al. 2020; Yamakawa et al. 2021; Zhang, Tang, et al. 2025). Recent population‐level analyses further revealed a relatively narrow genetic base among Chinese germplasms shaped by frequent introductions and admixture (Xiao et al. 2023).

Anthocyanins, a major class of flavonoid polyphenols, confer red, purple and blue pigmentation to plant tissues. Their biosynthesis follows a conserved enzymatic pathway initiated from phenylalanine and mediated by enzymes including chalcone synthase (CHS), chalcone isomerase (CHI), flavanone 3‐hydroxylase (F3H), dihydroflavonol 4‐reductase (DFR) and leucoanthocyanidin dioxygenase (LDOX/ANS), culminating in the production of anthocyanidins such as cyanidin. Subsequent modifications, including glycosylation by UDP‐glucosyltransferases (e.g., 3GGT) and methylation by anthocyanin O‐methyltransferases (AOMTs), enhance stability and vacuolar sequestration (Pourcel et al. 2010). This pathway is transcriptionally regulated by the MYB‐bHLH‐WD40 (MBW) transcriptional complex, with MYB proteins acting as key determinants of spatiotemporal specificity (Dong and Lin 2021). In sweet potato, IbMYB1 is a major regulator of anthocyanin accumulation in purple‐fleshed varieties (Mano et al. 2007; Tanaka et al. 2012; Hou et al. 2023), alongside core biosynthetic genes such as *PAL*, *CHS*, *CHI*, *F3H*, *DFR*, *ANS* and *UF3GT* (Lalusin et al. 2006). Anthocyanins also contribute to protection of the photosynthetic apparatus under high light and stress conditions (Li et al. 2023).

Leaf morphology further influences photosynthetic performance, as structural attributes optimise light capture and gas exchange (Bielczynski et al. 2017). Plants exhibit remarkable morphological diversity in leaf shape, a trait tightly associated with stress resilience and agronomic performance (Mukherjee et al. 2009; Bhatia et al. 2021). Leaf development is governed by conserved genetic modules, including the KNOX1‐BEL1 pathway (regulating lobing via TALE homeodomain TFs and auxin signalling) (Kumar et al. 2007; Wang et al. 2013; Furumizu et al. 2015; Jeon and Byrne 2021), the LMI1/RCO pathway (involving HD‐ZIP TFs in margin complexity) (Wang et al. 2021; Chang et al. 2019; Kierzkowski et al. 2019) and the Auxin‐CUC2 pathway (defining boundaries through NAC TFs and PIN1‐mediated auxin transport) (Bhatia et al. 2023). Although these pathways are well characterised in model species, the genetic basis of natural leaf shape variation in sweet potato remains poorly understood (Rosero et al. 2019; Jackson et al. 2020). GWAS studies have implicated *IbFBW2* on chromosome 7 and *IbYABBY1* on chromosome 12 as important regulators of leaf morphology (Chen et al. 2021; Xiao et al. 2023; Zhang, Tang, et al. 2025), and additional candidate genes have been proposed based on transcriptomic analyses (Gupta et al. 2020). However, functional validation and integration into coherent regulatory networks remain limited.

The genomic organisation of regulatory loci adds another layer of complexity. Plants frequently harbour tandemly duplicated transcription factor (TF) clusters, such as MYB or ERF genes, that regulate specialised processes (Shoji and Yuan 2021). Additionally, non‐homologous biosynthetic gene clusters (BGCs) assemble enzymes, TFs, transporters and regulators required for specialised metabolism (Zhan et al. 2022), facilitating coordinated expression, metabolic efficiency and adaptive inheritance (Nützmann et al. 2016). Whether such genomic clustering underlies pigmentation and leaf morphology diversification in sweet potato remains unresolved.

In this study, we resequenced 260 sweet potato germplasm accessions and integrated GWAS, transcriptomics and functional validation to dissect the genetic architecture of pigmentation and leaf morphology. Using a mixed linear model framework and rigorous variant filtering, we identified two tandem MYB TF clusters on chromosome 5 as core regulators of leaf anthocyanin accumulation. Comparative genomic and phylogenetic analyses revealed expansion and structural conservation of these clusters across subgenomes. Functional validation, together with emerging evidence for cooperative MYB regulation, supports a TF‐driven module underlying leaf pigmentation. In contrast, tuberous root pigmentation was associated with a spatially distinct cluster of structural anthocyanin biosynthetic genes, consistent with previous genetic mapping studies. Finally, a major locus on chromosome 7 harbouring BEL1‐like, LMI1‐like, TMK1, WD40 repeat‐containing and RABH1e genes, components of conserved developmental pathways, was identified as a central regulator of leaf morphology.

Together, these findings reveal organ‐specific regulatory architectures shaped by genomic clustering and conserved developmental networks, providing a comprehensive framework for breeding sweet potato varieties with optimised pigmentation and canopy architecture.

## 2 Results

### 2.1 Resequencing 260 Sweet Potato Accessions Reveals Structured but Admixed Populations

**FIGURE 1:**
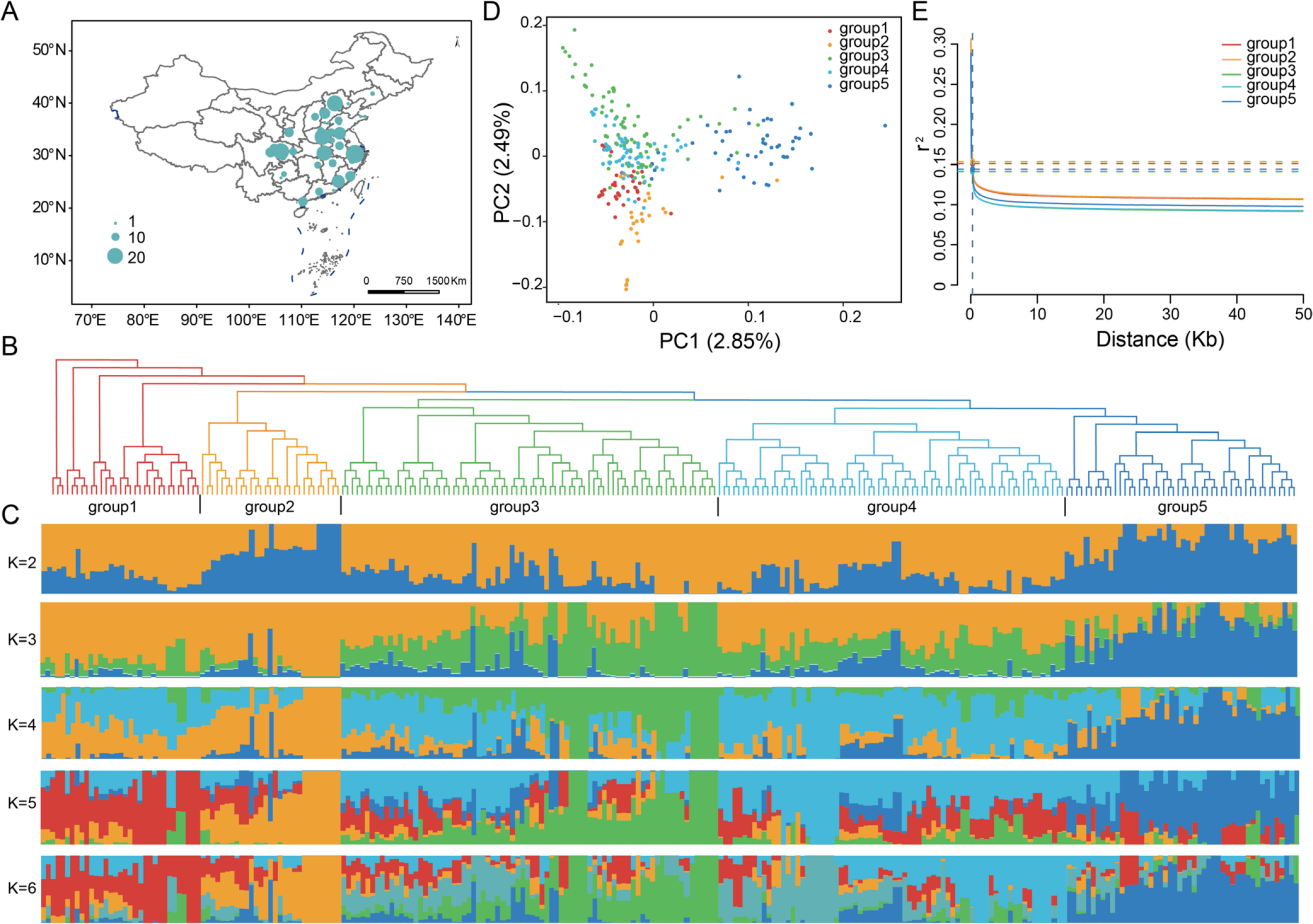
Population structure and genetic diversity of 260 sweet potato germplasms. (A) Geographical distribution of the sweet potato accessions across China. Circle size reflects the number of accessions collected from each location. (B) Maximum likelihood (ML) unrooted phylogenetic tree showing genetic relationships among the 260 accessions. Branch colours indicate inferred genetic subpopulations highlighting clustering patterns of related accessions. (C) Population structure inferred using ADMIXTURE at *K* = 2–6. Each vertical bar represents an individual accession, and colours indicate the proportion of ancestry derived from each inferred subpopulation. Accessions are ordered along the *x*‐axis according to their position in the ML tree. (D) Principal component analysis (PCA) of the 260 accessions based on genome‐wide SNP data. Each point represents one accession, and colours correspond to the inferred genetic subpopulations. The first two principal components are shown. (E) Linkage disequilibrium (LD) decay curves for the five sweet potato genetic subpopulations. Mean pairwise LD (*r* ^2^) was plotted against physical distance (kb) for each subpopulation, with lines coloured according to the respective subpopulation. Vertical dashed lines indicate the distance at which *r* ^2^ declined to half of its maximum value (half‐decay), and horizontal dashed lines show the corresponding *r* ^2^ thresholds. Filled coloured circles mark the intersection of the half‐decay distance and half‐max *r* ^2^ for each subpopulation. Half‐decay distances ranged from 0.244 to 0.276 kb.

To capture natural variation underlying pigmentation and leaf morphology, we resequenced 260 sweet potato accessions collected from diverse regions of China (Figure 1A and Table S1). Using the DNBSEQ‐T7 platform by MGI, sequencing generated 4670.66 Gb of raw data, averaging 17.96 Gb per sample, yielding 4556.56 Gb of high‐quality reads after filtering, averaging 17.53 Gb per sample (Table S2). Alignment to the Taizhong6 reference genome (Yang et al. 2017) identified 4 585 655 high‐confidence SNPs distributed across the genome (Figure S1A), including 964 329 exonic variants, of which 515 602 were nonsynonymous (Table S3). Unrooted phylogenetic analysis based on 236 923 fourfold degenerate SNPs resolved five genetic clusters (Figure 1B). Admixture analysis supported *K* = 5 as the optimal number of ancestral model (Figures 1C, S1B). However, nearly half of the accessions exhibited mixed ancestry, indicating extensive historical admixture. This ambiguous population structure likely reflects frequent introductions, complex crossbreeding and high gene flow, resulting in a narrow genetic base for Chinese sweet potato germplasms (Xiao et al. 2023). TreeMix analysis further inferred two major migration events (*m* = 2), revealing gene flow from group 1 to group 2 and from group 5 to group 4 (Figure S2A,B). The residual fit plot (Figure S2C) reveals additional covariance patterns among populations that are not fully explained by the tree alone, reinforcing the presence of admixture events. The f‐branch diagram (Figure S2D) summarises the extent of gene introgression, showing pronounced gene flow, particularly involving group 1, consistent with the migration edges identified in the TreeMix analysis. These results indicate that modern Chinese sweet potato germplasm reflects frequent introduction and recombination.

Principal component analysis (PCA) confirmed partial separation among subpopulations with considerable overlap (Figure 1D). Linkage disequilibrium (LD) decayed rapidly in all groups, with *r* ^2^ declining to half of its maximum value within 0.244–0.276 kb (Figure 1E). The extremely rapid LD decay observed in sweet potato likely reflects both biological and technical factors. In addition to high recombination and heterozygosity, the complex autohexaploid genome, frequent admixture among cultivars, reference‐genome bias and diploid‐based SNP calling may further contribute to reduced apparent LD estimates. Kinship analysis revealed low relatedness across the panel (median = −0.013), with only 0.14% of pairwise comparisons exceeding 0.5. Genomic inflation factors (*λ* = 0.95–1.03; mean 1.006) across 43 traits confirmed effective control of population structure. Collectively, these analyses demonstrate that this panel combines substantial genetic diversity and limited redundancy features that provide strong power for high‐resolution GWAS in a hexaploid genome.

### 2.2 Phenotyping Enables Reliable GWAS of Pigmentation and Leaf Morphology

We evaluated 43 agronomic traits across two years and two locations, including 35 quantitative and 8 qualitative traits (Tables S1, S4). To assess phenotypic reproducibility, pairwise correlations were first calculated among the four independent environments (2021BQ, 2021NZY, 2022BQ and 2022NZY). Most traits exhibited moderate to strong cross‐environment correlations (Figure S1C), supporting phenotypic stability. We further examined trait–trait relationships by Spearman's rank correlation analysis. Correlation analysis revealed structured relationships among traits with integrated values across these four environments, which were obtained by averaging quantitative traits and taking the mode for qualitative traits. (Table S1). The resulting data revealed significant associations among several traits, with three key categories (leaf colouration, tuberous root pigmentation and leaf shape) showing strong inter‐trait correlations (Figure S3). Broad‐sense heritability (*H* ^2^) varied widely: pigmentation and leaf morphology traits exhibited high heritability (often > 0.8), whereas yield‐related traits showed stronger environmental influence (Figure S1C and Table S4).

To better characterise the five genetic groups, we compared 43 phenotypic traits across all accessions. Twenty‐five traits showed significant differences among groups (FDR ≤ 0.05; Figure S4), whereas the remaining 18 traits did not differ significantly, indicating that the groups share core agronomic characteristics while exhibiting measurable phenotypic differentiation. All groups consist of cultivated sweet potato germplasm and share fundamental domestication traits. The most noticeable variation was observed in storage‐root pigmentation, with some groups showing a higher proportion of purple‐fleshed accessions. Variation was also present in leaf morphology, pigmentation, plant architecture and yield‐related traits, reflecting phenotypic diversity across groups while indicating partially shared genetic backgrounds.

Using 4 585 655 SNPs and a mixed linear model incorporating kinship and five principal components (Zhou and Stephens 2012), we conducted GWAS primarily using integrated phenotypic values (Table S1) to maximise genetic signal (Table S5). Several intercorrelated traits, such as leaf pigmentation, tuberous root pigmentation and leaf shape, showed overlapping association signals (Figures S7, S18, S23). Across four environments (2021BQ, 2021NZY, 2022BQ and 2022NZY), association peaks were highly consistent (Figures S8, S19, S24), confirming robustness. Furthermore, pairwise colocalisation analyses showed that multiple correlated traits within these categories share common genetic loci (PP.H4 > 0.8; Figure S5), with substantial overlap of candidate genes identified from GWAS‐significant SNPs (Table S6). These results establish a reliable genetic framework for dissecting pigmentation and leaf architecture.

### 2.3 Two Tandem MYB Clusters Underlie Leaf Anthocyanin Accumulation

**FIGURE 2:**
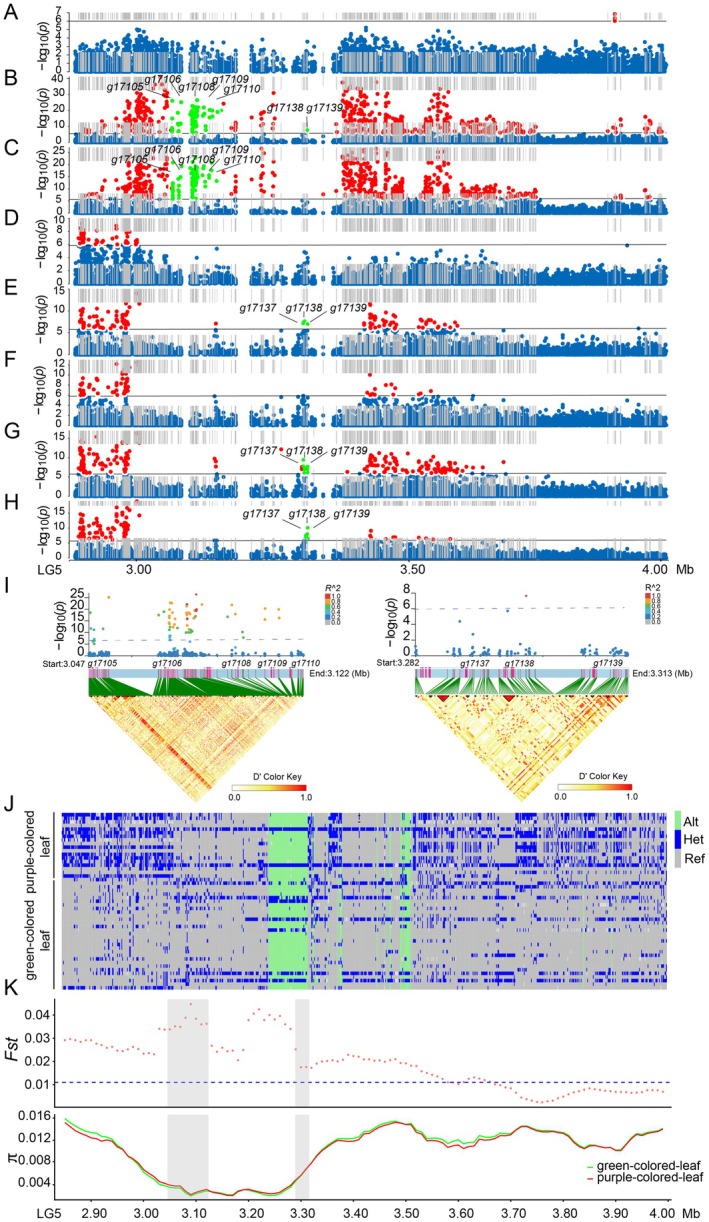
Two tandem MYB clusters regulate anthocyanin accumulation in leaves (A–H). Manhattan plots showing GWAS signals for eight leaf pigmentation traits within the genomic interval on chromosome 5 (LG5): 2 847 423–3 995 468, including immature leaf margin pigmentation (A), terminal bud pigmentation (B), immature leaf blade pigmentation (C), adaxial leaf vein pigmentation (D), abaxial leaf midvein pigmentation (E), abaxial leaf lateral vein pigmentation (F), pigmentation at the base of leaf vein (G) and pigmentation at the base of petiole (H). The horizontal black lines represent the genome‐wide significance threshold (*p* ≤ 1e‐06). Grey lines indicate SNP positions that exceed the significance threshold for at least one trait and are aligned across panels to facilitate comparison of association signals across traits; these lines serve only as visual guides and do not imply statistical colocalization. Significant SNPs within candidate MYB genes are highlighted in green, whereas other trait‐specific significant SNPs (*p* ≤ 1e−06) are shown as red points. Black lines indicate the genome‐wide significance threshold (1e‐06) on the Manhattan plots. (I) LD heatmaps of the two MYB cluster regions (LG5:3.047–3.122 Mb and LG5:3.282–3.313 Mb), showing strong LD and haplotype structure within each cluster. (J) Haplotype analysis of selected accessions within the GWAS interval (LG5: 2 847 423–3 995 468 bp), comparing purple‐leaf accessions (*n* = 18; pigmentation score ≥ 5 for multiple vein and petiole pigmentation traits) and green‐leaf accessions (*n* = 31; pigmentation score = 1). Distinct haplotype patterns are observed between phenotypic groups. (K) Population differentiation and nucleotide diversity within the MYB cluster region. Genetic differentiation (*Fst*) between purple‐ and green‐leaf accessions was calculated using a sliding window approach (window size = 100 kb; step size = 10 kb). Nucleotide diversity (π) was calculated separately for each group using the same parameters. Grey shaded regions indicate the candidate MYB clusters (cluster1: 3 045 131–3 122 000 bp and MYB cluster 2: 3 289 147–3 314 000 bp). The dashed blue line indicates the genome‐wide top 5% *Fst* threshold (0.011). Red and green lines correspond to π values for the purple‐ and green‐leaf groups, respectively.

Eight above‐ground pigmentation traits, including adaxial and abaxial vein colouration, immature leaf pigmentation and terminal bud colouration, were used to quantify leaf anthocyanin accumulation (Figure S6A). These traits exhibited strong positive correlations with integrated phenotypic values across four environments (Figure S6B) and skewed phenotypic distributions (Figure S6C), consistent with qualitative pigmentation transitions. All leaf pigmentation traits mapped to a prominent locus on chromosome 5 (LG5:2 847 423–3 995 468) (Figure S7). The eight leaf pigmentation‐related traits exhibited high broad‐sense heritability (*H* ^2^ = 0.657–0.881) (Figure S1C, Table S4), accompanied by consistent association signals across environments (Figure S8). Fine mapping identified ~130 annotated genes within the target interval. Among these genes, two tandem MYB clusters, including eight R2R3‐MYB transcription factors (g17105‐g17110; g17137‐g17139) – members of a family well known for its role in anthocyanin regulation – were identified as strong candidates for controlling leaf pigmentation (Figures 2A–H, S9A).

LD haplotype block analysis revealed tight linkage among significant SNPs within two tandem MYB clusters (Figure 2I). To specifically evaluate genetic differentiation associated with leaf pigmentation, accessions were classified into purple‐ and green‐leaf groups based on extreme pigmentation phenotypes across the full diversity panel (Figure S10A). These phenotype‐based groups are distinct from the SNP‐defined genetic subpopulations identified by structure analysis (groups 1–5; Figure 1), thereby allowing assessment of pigmentation‐associated divergence largely independent of underlying population structure. Haplotype analysis across the LG5:2 847 423–3 995 468 interval clearly distinguished purple‐ and green‐leaf accessions, demonstrating strong genotype–phenotype correspondence (Figure 2J). Population differentiation analysis using the fixation index (*Fst*) and nucleotide diversity (π) revealed elevated *Fst* values across the MYB cluster region, accompanied by moderate nucleotide diversity (Figure 2K). These patterns indicate pronounced genetic differentiation associated with leaf pigmentation variation and provide population‐genetic support for the MYB clusters as key determinants of anthocyanin accumulation.

### 2.4 Functional Validation of the Candidate *MYB* Cluster Genes

**FIGURE 3:**
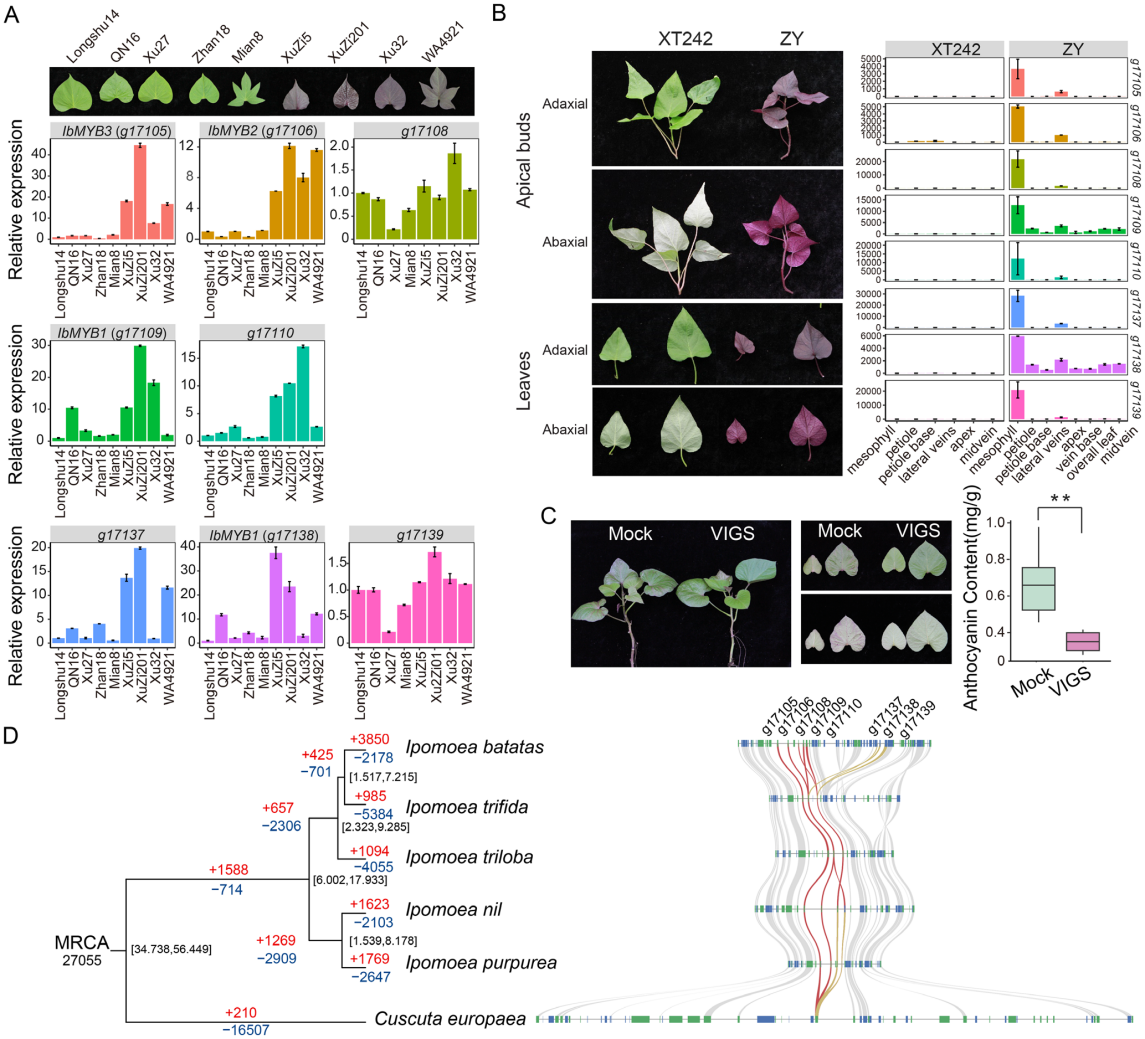
Functional and evolutionary analysis of *MYB* gene clusters regulating leaf anthocyanin accumulation. (A) Relative expression levels of two MYB cluster genes in selected sweet potato accessions. Longshu14, QN16, Zhan18, Mian8, Xu37 and WA4921 were obtained from the Zhejiang A&F University germplasm collection, whereas Xu27, XuZi201 and XuZi5 are maintained in our lab. (B) Tissue‐specific expression patterns of *MYB* cluster genes in different leaf tissues, including the midvein, lateral veins, mesophyll and petiole base, in green‐leaf (TX242) and purple‐leaf (ZY) accessions. Both accessions are from our laboratory collection. (C) Virus‐induced gene silencing (VIGS) of MYB cluster genes in the Long14 line. Representative phenotypes of plants subjected to VIGS targeting the conserved MYB domain and mock‐treated controls (empty vector), along with quantification of anthocyanin content. Data represent mean ± SE. Asterisks indicate statistically significant differences (*p* ≤ 0.01, as determined by Student's *t*‐test). (D) Comparative genomic and microsynteny analysis of MYB clusters across Ipomoea species, including *I. trifida* , *I. triloba* , *I. nil* , *I. purpurea* , with *Cuscuta europaea* as the basal group. Phylogenomic analysis indicates that *I. batatas* is most closely related to *I. trifida* and exhibits significant expansion of *MYB* cluster genes. Microsynteny analysis further demonstrates conservation and expansion of MYB clusters in *I. batatas* . Divergence times are indicated in brackets, and red and blue numbers denote expanded and contracted gene families, respectively. Coloured lines (red, yellow and grey) represent syntenic relationships between *MYB* cluster genes and adjacent genomic regions across species.

The two *MYB* gene clusters were identified as the most promising candidates associated with leaf anthocyanin accumulation (Figure 2). Homology‐based sequence alignment analysis confirmed that *g17109* and *g17138* correspond to *IbMYB1*, while *g17106* and *g17105* correspond to *IbMYB2* and *IbMYB3*, respectively (Tanaka et al. 2012; Mano et al. 2007). These MYB proteins show strong conservation within the R2R3 domain and cluster phylogenetically with anthocyanin‐related homologues from Ipomoea species, including *I. trifida* (ItrifidaMYB75), *I. triloba* (ItrilobaMYB113) and *I. nil* (InMYB75), as well as functionally characterised *Arabidopsis* regulators, such as AtPAP1, AtMYB90 and AtMYB113/114 (Figure S9B,C). Comparative genomic analysis across *Convolvulaceae* species revealed that *I. batatas* is most closely related to *I. trifida* but exhibits a marked expansion of *MYB* genes. Microsynteny analysis confirmed conservation and lineage‐specific expansion of these MYB clusters, suggesting an evolutionary basis for the diversification of anthocyanin regulation in sweet potato (Figure 3D).

To validate the functional relevance of the GWAS‐identified MYB clusters, we first examined transcript abundance across nine accessions with contrasting leaf pigmentation. qRT‐PCR analysis demonstrated significantly higher expression of cluster *MYB* genes in purple‐leaf accessions compared to green‐leaf accessions (Figure 3A). Spatial expression profiling across mesophyll, petiole, petiole base, lateral veins, apex, vein base and midvein tissues revealed a strong correspondence between *MYB* transcript abundance and anthocyanin distribution, with elevated expression in pigmented tissues and uniformly low expression in non‐pigmented accessions (Figure 3B). Consistent with these findings, RNA‐seq analysis identified 3104 differentially expressed genes between green and purple terminal buds (Figure S11A,B), including six *MYB* cluster genes that were strongly upregulated in purple accessions, with *IbMYB2* and *IbMYB3* showing much higher expression levels than other *MYBs* (Figure S11C,D, Table 1). These results demonstrate tight transcriptional coupling between *MYB* cluster activation and anthocyanin accumulation.

**TABLE 1:** Candidate genes for leaf pigmentation revealed by combined GWAS and RNA‐seq analyses.

Gene annotation_geneid	Position of Peak SNP	Ref	Alt	*p* (GWAS)	BaseMean_green_leaves	BaseMean_purple_leaves	Log_2_FoldChange	*p* (RNAseq)
40S ribosomal protein S5_g10413	LG3:7098381	C	T	1.44E‐07	13399.62	4855.80	−1.46	3.71E‐06
Protein argonaute 4_g11045	LG3:11992197	T	G	6.59E‐07	507.62	236.47	−1.1	1.70E‐07
BTB/POZ domain‐containing protein At1g67900‐like_g11919	LG3:18575262	A	G	6.43E‐08	55.99	320.59	2.51	2.81E‐13
Putative late blight resistance protein homologue R1A‐10_g12030	LG3:19275155	T	C	1.15E‐07	322.29	751.90	1.22	3.04E‐04
g14350	LG4:11415252	G	A	7.86E‐10	299.48	1612.56	2.43	3.91E‐05
Galactinol synthase 2_g17011	LG5:2474404	T	A	9.11E‐07	17.41	79.69	2.2	8.11E‐06
Dihydroflavonol 4‐reductase (DFR)_g17019	LG5:2515633	T	A	2.58E‐07	681.63	6315.51	3.21	5.65E‐09
Dihydroflavonol 4‐reductase (DFR) partial_g17020	LG5:2515633	T	A	2.58E‐07	822.33	20141.22	4.61	9.12E‐17
Scarecrow‐like protein 15_g17059	LG5:2748502	A	G	1.36E‐08	524.18	1212.49	1.21	3.38E‐03
IbMYB3_g17105	LG5:3053618	T	C	7.63E‐26	154.73	1175.23	2.93	2.46E‐09
IbMYB2_g17106	LG5:3053618	T	C	7.63E‐26	255.46	2393.93	3.23	6.09E‐08
MYB‐related protein 308‐like_g17108	LG5:3097059	C	G	1.49E‐22	5.64	27.79	2.28	1.19E‐03
IbMYB1_g17109	LG5:3113741	C	A	3.82E‐22	0.32	72.21	7.63	2.81E‐15
MYB transcription factor_g17110	LG5:3113741	C	A	3.82E‐22	0.00	12.62	6.07	1.78E‐07
IbMYB1_g17138	LG5:3298932	T	G	8.05E‐11	12.26	66.96	2.45	4.36E‐04
g17149	LG5:3381680	C	T	1.35E‐35	0.92	2902.98	11.75	2.73E‐64
Protein PHLOEM PROTEIN 2‐LIKE A10‐like_g17171	LG5:3555471	C	T	3.67E‐34	15.82	82.30	2.38	2.45E‐04
Uncharacterised protein LOC104648891_g17190	LG5:3685073	G	A	5.71E‐08	23.55	1.75	−3.77	1.07E‐04
30S ribosomal protein 3, chloroplastic‐like_g18875	LG5:16202048	G	A	2.77E‐09	123.81	1048.18	3.08	8.31E‐09
Uncharacterised protein LOC105167219_g21343	LG6:3649510	C	A	7.20E‐07	330.61	150.39	−1.14	1.46E‐03
g21501	LG6:4939288	A	G	5.04E‐07	2.69	44.75	4.08	4.05E‐05
g51002	LG12:30601293	A	C	4.41E‐07	5.06	26.06	2.4	9.40E‐04

Comparative genomic analysis using newly generated PacBio HiFi assemblies from three purple‐fleshed cultivars with contrasting aerial pigmentation (Ayamurasaki and WA4921 with purple immature leaves, XuZi8 with green immature leaves) and the Tanzania reference genome revealed that *MYB* genes are organised in tandem clusters and exhibit lineage‐specific duplication and copy number variation (Figure S12A,C). *IbMYB2* and *IbMYB3* showed multiple tandem copies in some accessions, whereas *IbMYB1* displayed fewer copies and a more conserved genomic structure. No fixed copy number differences consistently segregated with leaf colour, indicating that regulatory polymorphisms, gene dosage effects, or their combined influence likely contribute to pigmentation diversity. Consistently, sequence analysis identified numerous SNPs in coding and regulatory regions of MYB cluster genes, including exonic variants in *g17105* and *g17138* and promoter polymorphisms in *g17106*, *g17108* and *g17110*, suggesting potential functional and regulatory divergence (Figure S13 and Table S7).

To test functional necessity, we performed virus‐induced gene silencing (VIGS) targeting conserved regions within the two *MYB* clusters, specifically in the 3′ regions that show low homology to other MYB family members (Figure S14), resulting in approximately 70% reduction in anthocyanin content and strong downregulation of *MYB* transcript levels, confirming their essential role in leaf pigmentation (Figures 3C, S15B). Off‐target effects were excluded through phylogenetic and expression analyses of closely related MYB homologues (Figure S15). These results establish that the tandem MYB clusters are not only genetically associated with leaf pigmentation but are also transcriptionally activated in pigmented tissues and functionally required for anthocyanin accumulation.

**FIGURE 4:**
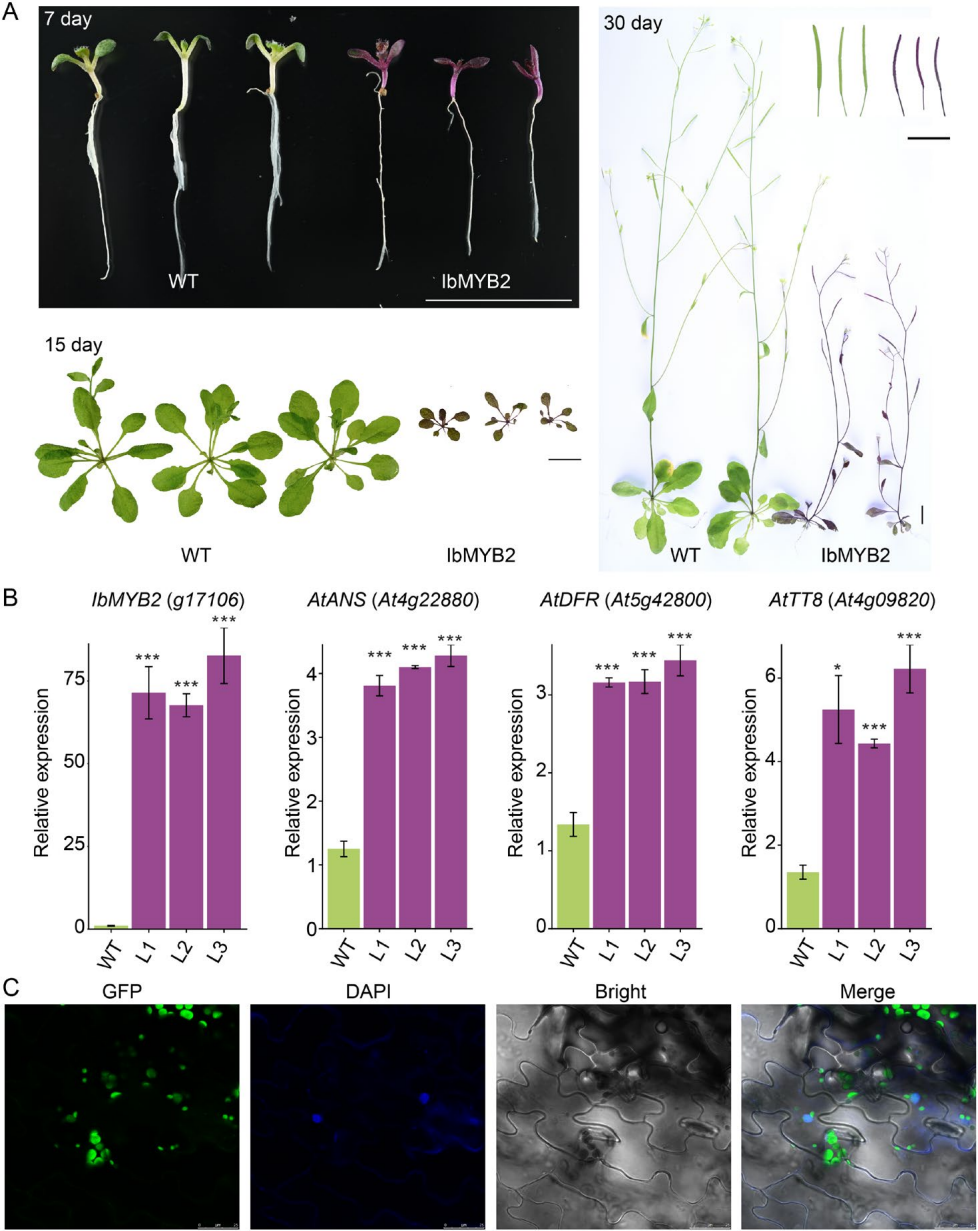
Functional characterisation of IbMYB2 by heterologous expression in *Arabidopsis thaliana* . (A) Phenotypic comparison between wild‐type (WT; ecotype Columbia) and transgenic Arabidopsis plants expressing *IbMYB2*. Transgenic lines exhibit enhanced purple pigmentation compared with WT plants. Scale bar = 1.0 cm. (B) qRT‐qPCR analysis of *IbMYB2* and anthocyanin biosynthetic genes in WT and *IbMYB2*‐expressing lines. Expression levels were normalised to the internal reference gene, and data are shown as mean ± SE of three biological replicates (*n* = 3). Statistical significance was determined using Student's *t*‐test (*p ≤ 0.05, **p ≤ 0.01, ***p ≤ 0.001). (C) Subcellular localisation of the IbMYB2 protein in Arabidopsis. IbMYB2‐GFP fluorescence is localised to the nucleus, consistent with its role as a transcription factor. DAPI staining was used as a nuclear marker. Scale bar = 25 μm.

Furthermore, *IbMYB2* was expressed in Arabidopsis under its native promoter. Transgenic plants overexpressing *IbMYB2* exhibited pronounced purple pigmentation throughout aerial tissues, including cotyledons, leaves, stems and siliques, whereas no visible pigmentation was observed in roots (Figure 4A). Correspondingly, endogenous structural genes (*DFR*, *ANS* and *TT8*) were upregulated (Figure 4B). Nuclear localisation confirmed that IbMYB2 co‐localises with DAPI in the nucleus (Figure 4C).

Promoter analysis using PlantCARE identified multiple MYB‐binding *cis*‐acting elements within the MYB cluster promoters, including canonical MYB motifs (TAACCA, CAACCA, CAACAG), MYB‐like sequences (TAACCA) and Myb/Myb‐binding site motifs (TAACTG, CAACAG). The abundance of these putative MYB‐binding sites varied among promoters: *IbMYB3* and *IbMYB2* contained eight to nine sites, whereas the *IbMYB1* promoter harboured seven sites (Figure S16A), suggesting differential potential for MYB‐mediated transcriptional regulation. To test whether these MYB proteins directly regulate one another, we performed a transient dual‐luciferase‐based transactivation assay (Figure S16B). The assays demonstrated that IbMYB2 and IbMYB3 strongly activate each other's promoters, whereas IbMYB1 showed minimal regulatory responsiveness (Figure S16C,D).

### 2.5 Anthocyanin Structural Gene Cluster Regulating Tuberous Root Pigmentation

Purple pigmentation in sweet potato tuberous roots is a key breeding trait associated with nutritional value and market preference. In this study, we identified 5 traits associated with tuberous root pigmentation: secondary colour of tuberous flesh, main colour of root cuticle, cortical flesh colour, main flesh colour and secondary colour distribution of tuberous flesh. These traits also exhibited strong correlations (Figure S17A) and similarly skewed distributions (Figure S17B). Four of these traits showed moderate‐to‐high heritability (*H* ^2^ = 0.36–0.957) (Figure S1C, Table S4). GWAS revealed strong association peaks at the terminal region of LG5 (19.5–30.5 Mb), which were consistently detected in both integrated analyses (Figure S18) and environment‐specific analyses using trait values from each of the two years and two locations (Figure S19).

**FIGURE 5:**
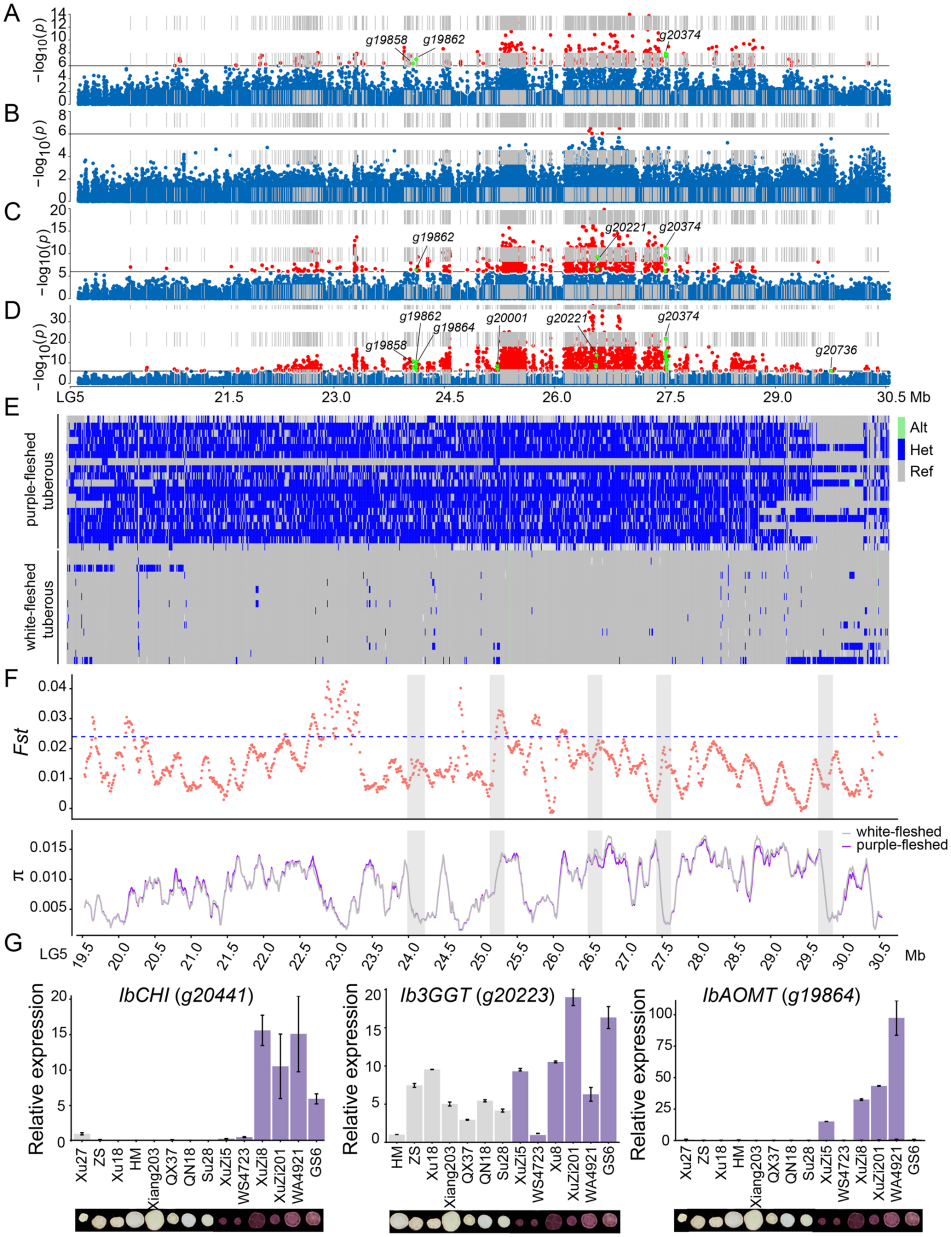
GWAS identification and functional validation of loci associated with tuberous root pigmentation. (A–D) Manhattan plots showing GWAS signals for 4 tuberous root pigmentation traits within the genomic interval on chromosome 5 (LG5:19 525 493–30 543 772), including secondary colour of tuberous flesh (A), main colour of root cuticle (B), cortical flesh colour (C) and main flesh colour (D). Significant SNPs within candidate anthocyanin biosynthesis‐related genes are highlighted in green, while other trait‐specific significant SNPs (*p* ≤ 1e‐06) are shown in red points. Grey connecting lines indicate shared physical positions of significant SNPs across traits and serve as visual guides; these do not imply statistical colocalisation. Black lines indicate the genome‐wide significance threshold (1e‐06) on the Manhattan plots. The horizontal black lines indicate the genome‐wide significance threshold (1e‐06). (E) Haplotype analysis of white‐ and purple‐fleshed accessions within the same genomic interval (LG5: 19 525 493–30 543 772), showing distinct haplotypic patterns between phenotypic groups. The white‐fleshed group included 16 accessions with pigmentation scores of < 8 for root cuticle colour and ≤ 1 for cortical flesh colour, main flesh colour and secondary colour. The purple‐fleshed group consisted of 19 accessions with pigmentation scores ≥ 9 for all four traits. (F) *Fst* and π analysis between white‐ and purple‐fleshed groups within the candidate region. *Fst* was calculated using a sliding window approach (window size = 100 kb; step size = 10 kb). Nucleotide diversity (π) was calculated separately for each group using the same parameters, and the dashed horizontal line indicates the top 5% genome‐wide *Fst* threshold (0.022). Grey shaded regions indicate candidate intervals identified by merging adjacent genes separated by ≤ 50 kb and extending 100 kb upstream and downstream. Purple and grey lines represent nucleotide diversity (π) for the purple‐ and white‐fleshed groups, respectively. (G) Relative expression levels of candidate anthocyanin biosynthetic genes *IbCHI* (*g20441*), *Ib3GGT* (*g20223*) and *IbAOMT* (*g19864*) in white‐ and purple‐fleshed accessions. Expression levels were normalised to the reference accession and are the mean ± SE.

High‐resolution mapping of LG5:19 525 493–30 543 772 interval identified 764 annotated genes (Table S10). Among these, multiple structural genes involved in anthocyanin biosynthesis and modification, such as *anthocyanin O‐methyltransferases* (*IbAOMT*; *g19858*, *g19862*, *g19864*, *g20001*), *anthocyanidin 3‐O‐glucoside 2‐O‐xylosyltransferase* (*Ib3GGT*; *g20221*) and *leucoanthocyanidin dioxygenase* (*IbLDOX*; *g20374*) (Figure 5A–D). Most significant variants associated with storage‐root purple pigmentation were located in intergenic or regulatory regions near *g19858*, *g19862*, *g19864*, *g20001*, *g20221* and *g20374*, indicating that *cis*‐regulatory variation likely plays a predominant role. In addition, a small number of coding changes, including nonsynonymous substitutions in *g20001* and *g20221*, may further contribute to phenotypic variation (Figure S20). Additional structural genes were also identified within this interval, despite lacking SNP variation, including *g19510*, *g19515* and *g19518* (*IbPAL*), *g19859* (*IbAOMT*), *g20223* (*Ib3GGT*) and *g20441* (*IbCHI*).

To further elucidate the genetic basis of tuberous root pigmentation, we compared accessions with extreme phenotypes. Purple‐ and white‐fleshed tuberous accessions were sampled broadly across the entire diversity panel, with a slight enrichment observed in subpopulation groups 5 and 3, respectively (Figure S10B). Comparison of extreme phenotypes (15 white‐fleshed vs. 19 purple‐fleshed accessions) revealed two distinct haplotype groups within the LG5 interval (Figure 5E). Although overall population differentiation was modest, localised peaks of elevated *Fst* were detected (Figure 5F), with AOMT (g20001) residing within one differentiated region, suggesting allele‐specific contribution to pigmentation intensity.

To identify causal genes responsible for anthocyanin accumulation in tuberous roots, we performed RNA‐seq analysis on five purple‐fleshed (WA4921, WS4723, XuZi8, XuZi201 and XuZi5) and four white‐fleshed cultivars (Xu18, Xu27, QN18 and Xiang203) (Figure S21A). PCA analysis separated the two groups, indicating distinct transcriptomic profiles (Figure S21B). RNA‐seq analysis identified 3478 DEGs (Table S11), of which 53 overlapped with the GWAS candidate genes, including 41 located within the LG5 association interval (Figure S21C,D, Table 2). Several anthocyanin biosynthesis‐related genes, including *IbAOMT* (*g19864* and *g20001*), *IbLDOX* (*g20374*) and *IbCCR* (*g20736*), showed significant differential expression (Table 2). qRT‐PCR confirmed elevated expression levels of *Ib3GGT* (*g20223*), *IbCHI* (*g20441*) and *IbAOMT* (*g19864*) in purple‐fleshed accessions compared to white‐fleshed controls (Figure 5G). These data demonstrate that tuberous root pigmentation is primarily governed by coordinated upregulation of structural anthocyanin biosynthetic genes.

**TABLE 2:** Candidate genes for tuberous root pigmentation revealed by combined GWAS and RNA‐seq analyses.

Gene annotation_geneid	Position of peak SNP	Ref	Alt	*p* (GWAS)	BaseMean_white_fleshed	BaseMean_purple_fleshed	Log_2_FoldChange	*p* (RNAseq)
g14266	LG4:10791829	C	T	4.82E‐08	0.10	14.12	5.94	3.60E‐05
g14267	LG4:10793275	C	T	5.13E‐12	0.40	18.21	5.59	6.07E‐05
Serine/threonine‐protein kinase HT1‐like_g17060	LG5:2757318	T	A	6.99E‐07	7.89	0.00	−5.42	9.65E‐08
14–3‐3‐like protein_g18871	LG5:16176459	C	T	7.55E‐07	35.60	91.76	1.36	1.08E‐05
Unknown protein_g19445	LG5:20831086	T	C	1.09E‐07	5.06	28.04	2.46	3.99E‐03
Unknown protein_g19600	LG5:22024337	A	T	3.81E‐08	16.36	129.49	2.98	1.91E‐04
Unknown protein_g19639	LG5:22276662	T	A	5.95E‐09	51.29	1.13	−5.52	5.41E‐06
Homeobox protein BEL1 homologue_g19659	LG5:22470035	G	C	9.21E‐10	15.07	114.46	2.93	3.70E‐09
g19674	LG5:22611243	A	G	3.08E‐07	41.19	0.69	−5.81	1.73E‐08
Unknown protein_g19677	LG5:22648422	G	T	1.04E‐13	110.96	46.29	−1.27	4.72E‐06
g19678	LG5:22653387	G	A	3.41E‐09	27.51	13.31	−1.05	4.93E‐03
Unknown protein_g19714	LG5:22975476	G	A	2.88E‐07	15.84	60.96	1.94	5.71E‐03
Peroxisomal fatty acid beta‐oxidation multifunctional protein AIM1_g19742	LG5:23206414	T	A	5.21E‐08	227.27	108.10	−1.07	6.79E‐04
Heat stress transcription factor A‐7a‐like_g19755	LG5:23309598	C	G	1.99E‐17	548.21	262.03	−1.07	6.18E‐03
Putative ABC transporter B family member 8_g19793	LG5:23658255	A	C	1.37E‐12	2056.53	779.78	−1.40	9.40E‐07
Phosphatidylinositol/phosphatidylcholine transfer protein SFH9‐like isoform X1_g19809	LG5:23726785	A	G	1.01E‐07	16.27	37.37	1.20	1.90E‐04
AOMT_g19864	LG5:24125089	C	T	2.11E‐11	5.75	15264.39	11.36	1.16E‐49
Leucine‐rich repeat receptor‐like protein kinase_g19873	LG5:24197570	C	T	3.29E‐08	39.30	137.89	1.81	5.83E‐05
Peroxisomal fatty acid beta‐oxidation multifunctional protein AIM1‐like_g19939	LG5:24718828	C	A	4.18E‐07	24.99	63.95	1.35	1.01E‐04
Probable polyamine oxidase 5_g19965	LG5:24956397	C	G	3.08E‐19	76.34	32.27	−1.24	2.30E‐03
Protein arginine N‐methyltransferase 1.1‐like_g19985	LG5:25097692	C	A	7.61E‐07	20.22	160.64	2.99	9.35E‐06
Probable leucine‐rich repeat receptor‐like protein kinase_g19989	LG5:25124452	T	C	1.20E‐07	36.82	141.24	1.94	5.76E‐08
AOMT_g20001	LG5:25223190	T	C	1.65E‐09	1.67	10108.91	12.52	4.61E‐60
Protein bem46 isoform X1_g20018	LG5:25336001	T	G	8.89E‐20	134.12	51.84	−1.37	4.69E‐04
GDSL esterase/lipase At5g03610‐like_g20070	LG5:25668172	C	T	1.37E‐08	23.81	7.35	−1.71	9.79E‐05
GDSL esterase/lipase At5g03610‐like_g20087	LG5:25811441	C	T	9.30E‐08	15.03	1.97	−2.93	1.01E‐03
TMV resistance protein N isoform X2_g20113	LG5:25956344	G	A	4.94E‐13	283.81	581.45	1.03	3.31E‐03
TMV resistance protein N‐like_g20167	LG5:26290139	T	C	2.08E‐14	688.59	1841.93	1.42	9.55E‐04
Unknown protein_g20225	LG5:26591130	G	A	1.20E‐16	0.00	24.63	7.11	1.23E‐09
Caffeoylshikimate esterase‐like_g20237	LG5:26675610	C	T	1.46E‐25	15.95	68.92	2.12	4.42E‐03
Zinc finger protein ZAT12‐like_g20250	LG5:26747089	A	C	5.93E‐17	0.38	7.76	4.36	2.74E‐04
Putative gag‐pol polyprotein, identical_g20259	LG5:26803879	G	A	5.73E‐12	1.93	10.58	2.43	5.51E‐03
CASP‐like protein PIMP1_g20274	LG5:26880351	T	A	6.85E‐31	7.01	73.75	3.39	6.12E‐05
Unknown protein_g20287	LG5:26945446	C	A	2.24E‐22	6.71	17.40	1.37	6.74E‐03
7‐deoxyloganetic acid glucosyltransferase‐like_g20330	LG5:27230011	C	T	8.34E‐12	26.04	102.02	1.97	9.81E‐04
WUSCHEL‐related homeobox 5‐like_g20352	LG5:27360134	T	C	2.90E‐18	12.97	0.92	−3.83	1.57E‐03
Cytochrome P450 90A1‐like_g20364	LG5:27451645	C	T	8.84E‐16	2737.50	1097.10	−1.32	2.93E‐05
LDOX_g20374	LG5:27507718	C	T	1.65E‐22	43.81	20.91	−1.07	4.38E‐03
Unknown protein_g20391	LG5:27719199	G	C	4.02E‐07	2.82	27.74	3.30	3.23E‐03
21 kDa protein‐like_g20505	LG5:28416102	T	C	3.69E‐07	2.15	14.05	2.68	5.60E‐03
protein EXORDIUM‐like 3_g20509	LG5:28446770	T	A	1.72E‐14	148.71	367.87	1.31	2.31E‐03
Protein TRANSPARENT TESTA 12‐like_g20601	LG5:29023026	C	T	6.24E‐07	2.48	521.58	7.71	7.02E‐18
ABC transporter G family member 8‐like_g20614	LG5:29114897	G	A	6.93E‐07	89.54	350.82	1.97	6.81E‐03
Probable inactive receptor kinase At4g23740‐like isoform X1_g20625	LG5:29183483	A	G	9.88E‐08	66.72	356.82	2.42	8.57E‐15
cinnamoyl‐CoA reductase 1‐like_g20736	LG5:29753405	T	G	6.54E‐07	168.20	738.29	2.13	1.72E‐11
Anthocyanin 5‐aromatic acyltransferase‐like_g21428	LG6:4269003	A	T	8.47E‐07	0.27	3.19	3.41	1.40E‐03
Unknown protein_g22441	LG6:11826186	G	A	5.67E‐11	9.51	208.43	4.45	4.78E‐03
Unknown protein_g26640	LG7:9918553	C	T	7.88E‐08	0.76	17.04	4.52	9.73E‐06
Probable LRR receptor‐like serine/threonine‐protein kinase At1g74360_g51265	LG13:816860	A	C	7.65E‐07	43.82	0.29	−7.04	4.51E‐11
Unknown protein_g59091	LG14:26538853	C	T	4.13E‐07	0.47	5.05	3.38	1.28E‐03
60S acidic ribosomal protein P0_g62022	LG15:15343047	C	A	5.78E‐07	5.53	35.12	2.67	4.24E‐04
Uncharacterised protein K02A2.6‐like_g63555	LG15:26702383	C	T	8.22E‐13	16.35	0.87	−4.22	3.02E‐05
U‐box domain‐containing protein 12_g8109	LG2:28839807	C	A	1.11E‐13	7.34	0.00	−5.33	4.31E‐04

To investigate whether the extended GWAS association signal reflects large‐scale structural rearrangements, we compared three purple‐fleshed genomes (Ayamurasaki, XuZi8 and WA4921) with the Tanzania reference. Within the GWAS‐associated interval (~19–30 Mb) on Taizhong6 LG5, no major structural variations, such as inversions or translocations, were detected across the corresponding A‐E subgenomes of Tanzania chromosome 12. Although a previously reported internal inversion was observed on chromosome 12F, this region likely represents a genuine genomic segment rather than an assembly artifact (Wu et al. 2025). Consistently, synteny analyses using contigs from Ayamurasaki, XuZi8 and WA4921 revealed strong collinearity with Tanzania chromosome 12 across all subgenomes, with no evidence of major structural variations (SVs) within the association interval (Figure S12D). These results suggest that the extended GWAS signal is unlikely to be driven by large‐scale chromosomal rearrangements. Instead, it more likely reflects sequence‐level polymorphisms and regulatory divergence within clustered anthocyanin biosynthetic genes. Collectively, these findings indicate that storage‐root pigmentation is controlled by a spatially distinct structural gene cluster on LG5, in contrast to the MYB transcription factor‐mediated module controlling leaf pigmentation.

### 2.6 Key Regulators Underlying Leaf Shape Variation

Leaf morphology influences both the visual characteristics of accessions and their photosynthetic efficiency (Bielczynski et al. 2017). We analysed eight leaf shape‐related traits, including overall shape, marginal serration type, sinus depth and lobe number, in both immature and mature leaves (Figure S22A). Similar to pigmentation traits, leaf shape traits exhibited skewed distribution patterns across accessions (Figure S22B). The eight leaf shape‐related traits exhibited high heritability, with *H* ^2^ values ranging from 0.787 to 0.942 (Figure S1C, Table S4).

**FIGURE 6:**
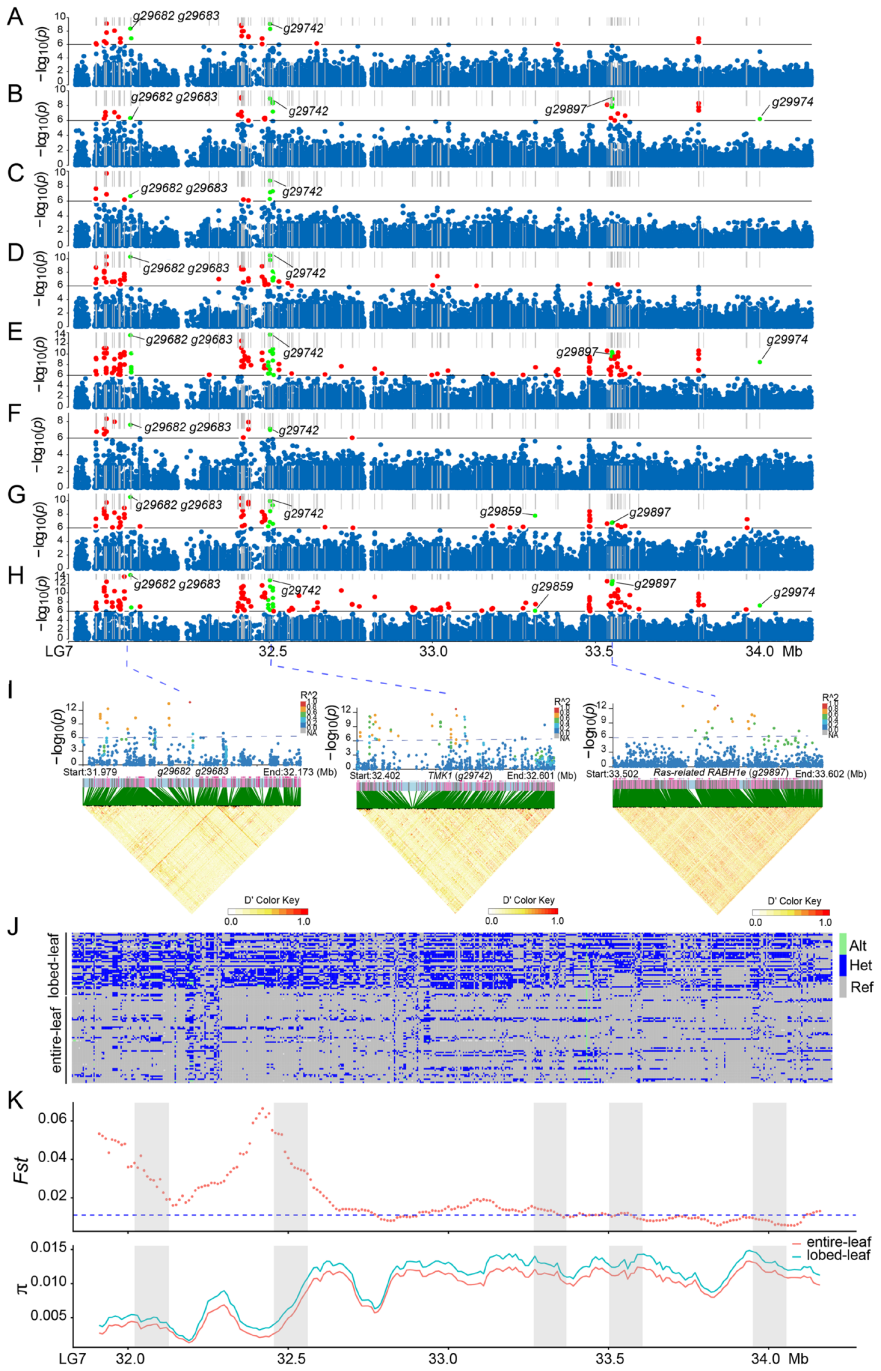
GWAS identification of genomic loci associated with leaf shape variation. (A–H) Manhattan plots showing GWAS signals for eight leaf shape traits within the genomic interval on chromosome 7 (LG7: 31 901 264–34 161 590): Shape of immature leaf (A), shape of mature leaf (B), lobe number of immature leaf (C), marginal serration type of immature leaf (D), sinus depth of immature leaf (E), lobe number of mature leaf (F), marginal serration type of mature leaf (G) and sinus depth of mature leaf (H). The horizontal black lines represent the threshold for statistical significance (*p* ≤ 1e‐06). (I) LD analysis of three major association peaks. LD blocks were defined as regions extending 100 kb upstream and downstream of the lead SNP for the first two peaks and 50 kb for the third peak. Candidate genes located within these LD blocks, including *TMK1* (*g29742*) and *RABH1e* (*g29897*), are highlighted. (J) Haplotype analysis comparing entire‐leaf and lobed‐leaf accessions within the GWAS interval (LG7: 31901264‐34 161 590). The lobed‐leaf group (*n* = 37) included accessions with immature and mature leaf shape scores of 6, sinus depth ≥ 3, lobe number ≥ 2 and marginal serration type ≥ 2. The entire‐leaf group (*n* = 64) consisted of accessions with lower scores for these traits. Distinct haplotype patterns are observed between the two phenotypic groups. (K) Genetic differentiation and nucleotide diversity analysis within the candidate region. Population differentiation (*Fst*) was calculated using a sliding window approach (window size = 100 kb; step size = 10 kb). Nucleotide diversity (π) was calculated separately for each group using the same parameters. The dashed horizontal line indicates the top 5% genome‐wide *Fst* threshold (0.01). Grey shaded regions indicate candidate intervals identified by merging adjacent genes separated by ≤ 50 kb and extending 100 kb upstream and downstream. Red and light blue lines represent nucleotide diversity (π) for the entire‐leaf and lobed‐leaf groups, respectively.

GWAS consistently identified a strong association signal at the terminal region of LG7 (31.9–34.2 Mb) across integrated trait values (Figure S23) and trait values from each of the two years and two locations (Figure S24). Fine‐mapping identified a key signal spanning LG7:31 901 264–34 161 590 (Figure 6A–H), which included three strong LD blocks containing the most significant association peaks (Figure 6I). Within this region, 145 candidate genes were associated with eight leaf shape GWAS regions, with 100 genes located within the LG7 interval (Table S12).

To further assess the genetic basis of leaf shape variation, we classified 37 accessions as the lobed‐leaf group and 64 accessions as the entire‐leaf group. These accessions were selected from across the full diversity panel, with a slight enrichment of entire‐leaf accessions in group 4 and lobed‐leaf accessions in groups 3 and 5 (Figure S10C). Haplotype analysis revealed distinct genetic patterns within the LG7 interval (31 901 264–34 161 590), consistent with their morphological differentiation (Figure 6J). Similarly, *Fst* analysis detected significant genetic differentiation between the lobed‐ and entire‐leaf groups across this region. Consistent with these findings, nucleotide diversity (π) differed between the two phenotypic classes, suggesting divergence at this locus associated with leaf shape variation. Notably, candidate genes *g29742*, *g29859* and *g29897* reside within these differentiated regions, providing additional support for their potential contribution to natural variation in leaf morphology (Figure 6K).

To capture early transcriptional differences associated with leaf shape divergence, we performed RNA‐seq analysis on young leaf tissues from four entire‐leaf and four lobed‐leaf accessions (Figure S25A). PCA did not fully separate the two groups, likely reflecting the polygenic and complex nature of leaf shape regulation (Figure S25B). Differential expression analysis identified 2166 DEGs between the two phenotypic classes (Table S13). Intersection of these DEGs with the 145 GWAS candidate genes (Table S12) identified 10 overlapping genes as strong candidates for regulating leaf shape (Figure S25C,D, Table 3). Further SNP‐level association analysis revealed multiple genome‐wide significant variants located within or proximal to five of these candidate genes (Figure S26), providing convergent genetic and transcriptional evidence supporting their roles in leaf shape variation.

**TABLE 3:** Candidate genes for leaf shape revealed by combined GWAS and RNA‐seq analyses.

Gene annotation_geneid	Position of peak SNP	Ref	Alt	*p* (GWAS)	BaseMean_entire_leaf	BaseMean_lobed_leaf	Log_2_FoldChange	*p* (RNAseq)
F‐box protein At5g49610‐like isoform X1_g1349	LG1:8126956	A	G	9.58E‐08	30.37	2.55	−3.56	8.85E‐07
Unknown protein_g20391	LG5:27716580	G	A	8.68E‐08	620.74	6.70	−6.54	2.48E‐09
WD40 repeat‐containing protein_g26165	LG7:5992365	C	T	6.45E‐16	39.61	103.05	1.38	1.04E‐03
TMK1_g29742	LG7:32501807	C	G	2.34E‐14	1314.40	520.18	−1.34	8.49E‐07
Unknown protein_g29821	LG7:33050627	C	T	1.61E‐07	1252.52	545.14	−1.2	1.08E‐03
LMI‐like_g29859	LG7:33316146	A	C	3.20E‐08	11665.56	6875.31	−0.76	6.81E‐06
Glutamate receptor 3.6‐like _g29866	LG7:33378393	T	G	2.13E‐07	432.96	143.10	−1.6	3.95E‐05
ras‐related protein RABH1e_g29897	LG7:33550527	A	G	8.45E‐13	526.52	143.09	−1.88	5.52E‐04
BEL1‐like_g29974	LG7:34003429	G	T	3.37E‐09	3825.87	2330.10	−0.72	4.72E‐05
GDSL esterase/lipase At1g28600‐like_g46175	LG11:35669393	C	T	4.14E‐07	926.28	378.09	−1.29	8.28E‐04

### 2.7 Functional Characterisation of Leaf Shape Regulators

**FIGURE 7:**
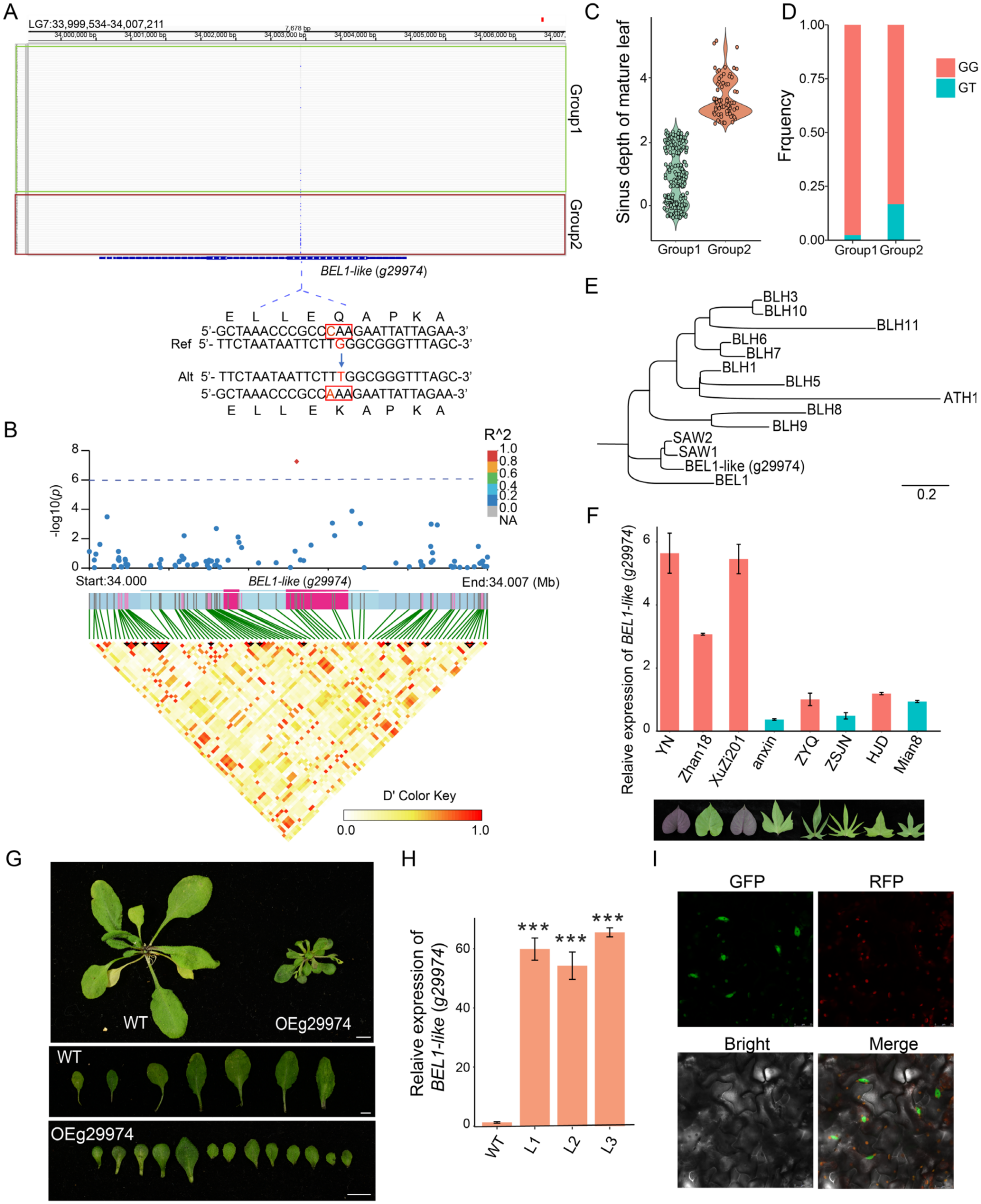
Genetic, expression and functional characterisation of the *BEL1*‐like gene (*g29974*) associated with leaf shape variation. (A) Integrative Genomics Viewer (IGV) visualisation showing haplotype variation of the *BEL1‐like* gene (*g29974*) between contrasting leaf shape groups. Gene structure and the nonsynonymous amino acid substitution are indicated. (B) LD analysis highlighting a significant SNP within the *BEL1‐like* gene (*g29974*) associated with leaf shape variation. (C) Distribution of sinus depth in mature leaves across phenotypic groups. Group 1 exhibits shallow or absent lobes (sinus depth scores 0–2), whereas Group 2 displays deeper lobes (scores 3–5). (D) Allele frequency distribution of the BEL1‐like SNP in the two phenotypic groups showing enrichment of the alternative allele in lobed‐leaf accessions. (E) Phylogenetic analysis of the BEL1‐like protein (g29974) and its Arabidopsis homologues SAW1/SAW2, indicating evolutionary conservation. (F) Relative expression levels of *g29974* in accessions with contrasting leaf shapes, determined by qRT‐PCR. Zhan18 and Mian8 were obtained from the Zhejiang A&F University, whereas YN, anxin, ZYQ, HJD, ZSJN and XuZi201 are maintained in our laboratory collection. Colours correspond to genotypes defined in panels A and D. (G) Representative phenotypes of wild‐type (WT; Columbia‐0) and *g29974*‐overexpressing (OEg29974) Arabidopsis plants. Overexpression of *g29974* resulted in reduced leaf size and suppressed vegetative growth. Scale bar = 1.0 cm. (H) qRT‐PCR analysis confirming elevated expression of *g29974* in transgenic lines relative to WT plants. Data represent mean ± SE. Statistical significance was determined using Student's *t*‐test (***p ≤ 0.001). (I) Subcellular localisation of the g29974 protein in Arabidopsis. GFP fluorescence indicates nuclear localisation, consistent with its function as a transcription factor. Scale bar = 25 μm.

Within the LG7 candidate interval, *g29974* emerged as a strong candidate gene for regulating leaf lobe depth. Integrative Genomics Viewer (IGV) and LD block analysis identified a significant SNP (LG7:34 003 429, G/T) strongly associated with leaf shape variation (Figures 7A,B, S26E). This SNP explained 11.42%, 14.30% and 12.03% of the phenotypic variance (PVE) for mature leaf outline, immature leaf notch depth and leaf notch depth, respectively (Table S5). This polymorphism results in a glutamine‐to‐lysine substitution (Q → K), suggesting potential functional consequences at the protein level. On the basis of sinus depth, we divided the accessions into two groups. Group 1, characterised by shallow or absent lobes, exhibited a low allele frequency of the alternative G/T allele (2.4%). In contrast, Group 2, displaying deeply lobed leaves, showed a markedly elevated allele frequency of 16.7%, highlighting a strong association between this SNP and leaf lobe development (Figure 7C,D). Deep resequencing further confirmed allelic differentiation: all six entire‐leaf accessions (YN, Zhan18, XuZi201, XuZi5, Xu32 and QN16) carried the reference allele (G/G), whereas six of eight lobed‐leaf accessions (Anxin, ZSJH, Mian8, Xu37, XuZi8 and WA4921) harbored the alternative allele (G/T), with the exception of ZYQ and HJD. This consistent genotype–phenotype association strongly supports a functional role for *g29974* in regulating leaf sinus depth.

Sequence analysis revealed g29974 as a BEL1‐like TALE homeodomain TF. In Arabidopsis, BEL homologues SAW1 and SAW2 regulate leaf serration, with reduced expression associated with increased lobe depth (Kumar et al. 2007). Phylogenetic analysis placed g29974 in a sister clade with SAW1 and SAW2, indicating close evolutionary conservation (Figure 7E). Consistent with this relationship, *g29974* expression was significantly lower in lobed‐leaf accessions compared with entire‐leaf accessions (Figure 7F), suggesting a conserved role in repressing excessive leaf margin dissection.

To validate the biological function, we generated transgenic Arabidopsis plants overexpressing *g29974* under the constitutive 35S promoter. Compared with wild‐type controls (WT, Col‐0), the overexpression lines (OEg29974) exhibited markedly reduced leaf size and overall suppressed vegetative growth (Figure 7G), resembling phenotypes observed in 35S:SAW1 overexpression in Arabidopsis (Kumar et al. 2007). qRT‐PCR confirmed strong transgene expression in independent lines (Figure 7H). Subcellular localisation analysis demonstrated that the g29974 protein is localised to the nucleus (Figure 7I), consistent with its predicted function as a transcriptional regulator. Together, these results indicate that g29974 likely functions as a BEL1‐like homeodomain protein that negatively regulates leaf growth and modulates leaf margin development.

We also identified *g26165*, encoding a WD40 repeat‐containing protein, as a major candidate regulator of leaf morphology. Three intronic SNPs (LG7:5 992 365; LG7:5 992 371; LG7:5 992 387; C/T) were consistently associated with all eight leaf shape traits (Figures S26A, S27A–I). These SNPs explained 10%–26% of PVE, with the strongest effects observed for notch depth traits (Table S5). Six major genotypes were defined, with genotype1 (209 accessions) matching the reference genome and genotype 6 (41 accessions) showing complete heterozygosity (Figure S27J). Notably, genotype 6 was significantly enriched in deeply lobed accessions (Figure S27K). Consistent with this association, *g26165* expression was significantly upregulated in lobed‐leaf accessions (Figure S27L). Genotypic analysis of three accessions confirmed that QN16 and Xu32 (entire‐leaf accessions) carried genotype 1, whereas Xu37 (lobed‐leaf accession) carried genotype 6. To functionally validate its role of *g26165* in leaf morphology, we generated CRISPR/Cas9‐mediated knockout lines in the lobed‐leaf cultivar Xushu22. Three independent edited lines carrying small insertions or deletions were obtained (Figure S27M). All knockout lines exhibited reduced leaf lobe depth and significantly lower dissection index (LDI) compared with the wild type (Figure S27N,O), demonstrating that *g26165* promotes leaf lobing in sweet potato.

Another strong candidate gene, *g29859*, encodes a homologue of Arabidopsis LATE MERISTEM IDENTITY1 (LMI1), a key regulator of leaf shape and meristem identity (Saddic et al. 2006; Chang et al. 2019). Two significant SNPs (LG7: 33 314 665 and LG7: 33 316 146), located in the upstream regulatory region of *g29859* (Figure S26C), each explained approximately 11%–16% of the phenotypic variance, with a stronger effect observed for Type of leaf lobe (Table S5). These variants defined five major haplotypes across the population (Figure S28A,B). Among these, genotype 1 (144 accessions), genotype 3 (83 accessions) and genotype 4 (23 accessions) were the three most predominant genotypes. Notably, accessions in group 2, characterised by deeper sinus depth (Figure 7C), exhibited significantly higher allele frequency of genotype 3 and genotype 4 compared to group 1 (Figure S28C). Consistent with this association, *g29895* expression was significantly downregulated in lobed‐leaf accessions (Figure S28D). Genotypic analysis of three accessions (QN16 and Xu37) revealed that QN16 (entire‐leaf) carried genotype 1, while Xu37 (lobed‐leaf) carried genotype 2.

To further validate the functional role of *g29859* in leaf morphogenesis, heterologous overexpression analyses were performed in Arabidopsis. Functional validation using Arabidopsis overexpression lines showed that ectopic expression of *g29859* (OEg29859) enhanced leaf lobing compared to WT plants (Figure S28E). Elevated expression was confirmed by qRT‐PCR (Figure S28F). These results support g29859 as a conserved regulator of leaf margin development.

Two additional candidate genes were supported by GWAS and expression evidence. *g29742*, encoding a TMK1 receptor‐like kinase, resides within a high‐LD interval (Figures 6I and S29A). Expression was significantly reduced in lobed‐leaf accessions (Figure S29B). In Arabidopsis, TMK1 mediates auxin‐dependent signalling pathways controlling cell expansion and growth (Friml et al. 2022), suggesting a role in hormone‐mediated leaf morphogenesis. Similarly, *g29897*, encoding a Ras‐related RABH1e protein involved in vesicle trafficking, was downregulated in lobed‐leaf accessions (Figure S25D), implicating intracellular trafficking processes in shaping leaf margin architecture.

## 3 Discussion

This study was guided by the overarching hypothesis that phenotypic diversity in sweet potato, particularly leaf and tuberous root pigmentation, as well as leaf morphology, is governed by both tissue‐specific genetic programs and shared regulatory frameworks shaped by environmental adaptation and evolutionary pressures. By integrating GWAS, RNA‐seq, qRT‐PCR and functional assays such as VIGS, we dissected the genetic architecture underlying pigmentation and leaf shape variation in 260 diverse germplasm accessions.

**FIGURE 8:**
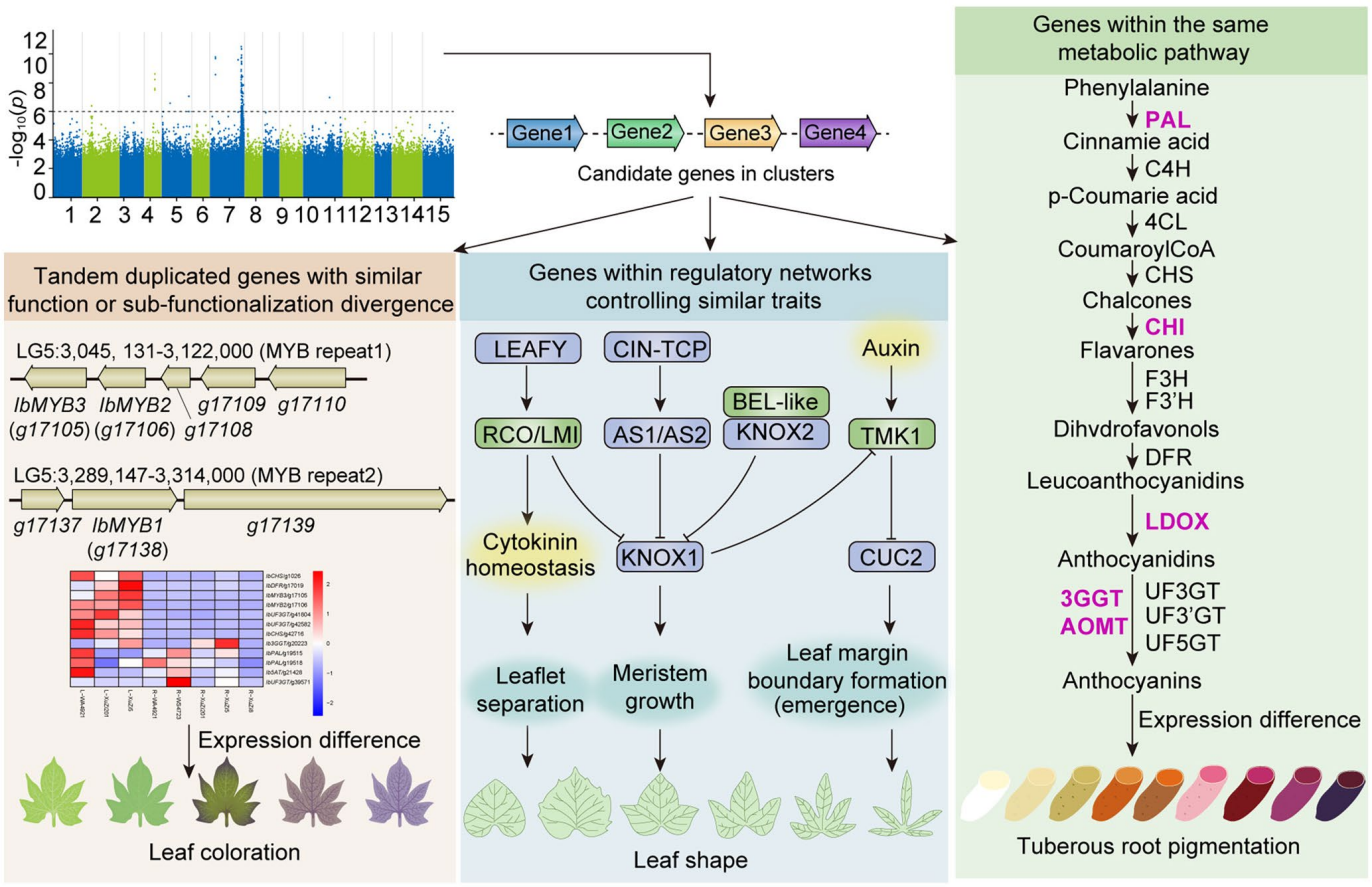
Conceptual model illustrating the genetic architecture underlying key agronomic traits identified by GWAS in sweet potato. This model summarises the genetic framework controlling major phenotypic traits, including leaf colouration, tuberous root pigmentation and leaf shape, which show strong and consistent GWAS association signals. Fine mapping of these loci revealed that candidate genes are frequently organised into genomic clusters, including tandemly duplicated transcription factor genes (left), structural genes within the same metabolic pathway (right) and regulators within interconnected developmental or signalling networks (middle). Such genomic clustering likely facilitates coordinated regulation and contributes to the accumulation of significant association signals within discrete chromosomal regions. Moreover, differential expression of these candidate genes among sweet potato accessions is proposed to drive phenotypic variation, reflecting functional divergence and adaptive specialisation.

Our findings demonstrate that leaf anthocyanin pigmentation is primarily controlled by two tandem clusters of MYB transcription factors on LG5. Transcriptomic and functional analysis suggested that IbMYB2 and IbMYB3 serve as central activators of anthocyanin biosynthesis in leaves (Table 1, Figures 4, S11, S16), while structural genes clustered at the terminal region of LG5 underlie pigmentation in tuberous roots (Figure 5). From an evolutionary standpoint, divergent transcription factors in leaves evolved to provide adaptive plasticity, while divergent enzymes in roots evolved to provide metabolic robustness and precision. Together, these complementary strategies reflect how different organs balance flexibility versus stability in pigment biosynthesis according to their ecological roles. These results expand the classical model of anthocyanin regulation in sweet potato, highlighting the role of gene clustering and differential expression in generating diverse pigmentation patterns (Figure 8).

IbMYB1 was the first transcription factor identified as a key regulator of anthocyanin biosynthesis in sweet potato, playing a role in both tuberous roots and leaves (Mano et al. 2007; Deng et al. 2020; Li et al. 2019; Dong et al. 2026). Through eQTL mapping and GWAS analyses, IbMYB1 has been confirmed as a crucial regulator of anthocyanin biosynthesis (Chen et al. 2021; Xiao et al. 2023). Recently, deep sequencing of 294 sweet potato accessions, combined with whole‐genome allele dosage variation mapping, further linked IbMYB1 to skin colour determination (Zhang, Tang, et al. 2025). Additionally, subsequent studies have implicated IbMYB2 and IbMYB3 as positive regulators (Deng et al. 2020; Wang et al. 2025). Our functional analysis demonstrates that *IbMYB2*, when expressed in Arabidopsis under the control of its native promoter, induces robust anthocyanin accumulation and activates endogenous biosynthetic genes, confirming its conserved regulatory role (Figure 4). Moreover, reciprocal activation between *IbMYB2* and *IbMYB3* suggests the existence of a self‐reinforcing transcriptional module that enhances pathway activation. Similar cooperative regulatory interactions have been reported among transcription factor networks controlling specialised metabolism (Paul et al. 2020), supporting the biological plausibility of this regulatory mechanism.

However, we also observed that in some population mapping studies, significant association peaks were detected, but they were notably distant from the physical location of the *IbMYB1* gene. For instance, a SNP at chromosome Chr12:20 608 324 was strongly associated with anthocyanin content in storage roots based on polyploid QTL‐seq. However, the location of the *IbMYB1* homologue in *I. trifida* on chromosome 12 (2.4–2.5 Mb in v3 NSP306) is far from the QTL identified at Chr12:20 608 324 (Yamakawa et al. 2021). In another study, the major GWAS signal was near Itr_chr05_27 090 704, while the *IbMYB1* homologue was located at Itr_chr05:2 573 593–2 574 056, indicating a substantial physical separation (Yada et al. 2017). These positional discrepancies suggest that other MYB‐related or unidentified genes may contribute to anthocyanin accumulation in sweet potato. In this study, we identified a tandem cluster of eight MYB genes associated with leaf anthocyanin biosynthesis (Table 1). Although *IbMYB1* showed relatively low basal expression in leaves (Table 1), its expression was high and markedly upregulated in purple‐fleshed cultivars (Table S11), consistent with its well‐established role in regulating anthocyanin accumulation in tuberous roots (Mano et al. 2007; Park et al. 2015). Intriguingly, the genomic location of *IbMYB1* did not coincide with the major GWAS signals for root pigmentation (Figures 2, 5), which instead encompassed several structural anthocyanin biosynthetic genes (Figures 5A–D, S20). The association interval contained structural biosynthetic genes whose expression patterns closely matched pigmentation phenotypes (Figure 5G, Table 2; He et al. 2020). These findings suggest that structural gene variation, rather than transcription factor variation, may play a dominant role in determining root pigmentation, highlighting a shift in regulatory hierarchy between organs.

Why different tissues rely on distinct regulatory mechanisms remains an intriguing question. We propose that anthocyanin accumulation in aerial tissues is highly dynamic and environmentally responsive, requiring flexible transcriptional regulation by MYB transcription factors (Dong and Lin 2021; Liu et al. 2023). In contrast, anthocyanin accumulation in storage roots appears to be developmentally programmed, with pigment levels rising during root maturation and stabilised by post‐synthetic modifications such as acylation and glycosylation (Wang, Wang, Fan, et al. 2018). This distinction between transcriptional plasticity and enzymatic stability represents an adaptive strategy that allows plants to balance environmental responsiveness with metabolic robustness.

In parallel with pigmentation, leaf morphology was strongly associated with a genomic interval on LG7 harbouring five candidate regulators: BEL1‐like (g29974), LMI1‐like (g29859), TMK1 (g29742), WD40 repeat‐containing protein (g26165) and RABH1e (g29897) (Figures 6, S26, Table 3). These genes represent three conserved developmental pathways, the BEL‐KNOX, RCO/LMI and auxin‐CUC/TMK modules, that collectively determine leaf lobing, serration and overall architecture (Kumar et al. 2007; Furumizu et al. 2015; Jeon and Byrne 2021; Wang et al. 2021; Chang et al. 2019; Kierzkowski et al. 2019; Bhatia et al. 2023). Rather than implying that all five genes are causal for a single trait, these candidates may contribute to distinct but related sub‐traits of leaf morphology within the associated interval. SNP‐haplotype analyses, expression profiling and phylogenetic comparisons revealed that allelic variation in these loci underlies the striking morphological differences among accessions (Figures 6, S25, S26). Moreover, functional validation experiments, including heterologous overexpression in Arabidopsis and CRISPR/Cas9‐mediated gene knockout in sweet potato (Figures 7, S27, S28), confirmed causal roles for several of these genes in shaping leaf morphology, indicating evolutionary conservation of developmental regulatory mechanisms.

Among these candidates, the BEL1‐like gene *g29974* showed strong genetic association with leaf lobing and shared evolutionary ancestry with Arabidopsis SAW1 and SAW2, which repress excessive leaf serration (Kumar et al. 2007). Reduced expression of *g29974* in lobed accessions and its growth‐suppressive effects when overexpressed support its role as a negative regulator of leaf margin outgrowth (Figure 7G,H). Similarly, *g29859*, an LMI1 homologue and *g26165*, a WD40 repeat‐containing gene, were strongly associated with leaf lobing and validated through functional assays. The identification of TMK1 and RABH1e homologues further implicates auxin signalling and intracellular trafficking in regulating leaf shape, highlighting the integration of transcriptional, hormonal and cellular regulatory processes. Unlike *lmi1* mutants in Arabidopsis, where lobing is lost (Vuolo et al. 2018), *g29859* was downregulated in lobed‐leaf accessions, suggesting functional divergence despite sequence conservation (Figure S28D, Table 3). Transgenic Arabidopsis plants overexpressing *g29859* exhibited enhanced leaf lobing compared with wild‐type controls (Figure S28E,F).

Notably, the strongest GWAS signals corresponded to three major trait categories – leaf pigmentation, root pigmentation and leaf morphology, suggesting genomic clustering of functionally related genes. Similar clustering has been reported in sweet potato and other plant species (Yang et al. 2024; Xiao et al. 2023; Zhang, Lyu, et al. 2025; Zhan et al. 2022). Such clustering may facilitate coordinated gene regulation, efficient inheritance of adaptive traits and rapid evolutionary diversification. The tandem MYB clusters and structural biosynthetic gene clusters identified here exemplify this genomic organisation and provide insight into how complex traits evolve through modular regulatory architecture.

Importantly, our results point to a conceptual connection between pigmentation and morphology as components of adaptive plant responses. Anthocyanins protect the photosynthetic apparatus against photoinhibition and oxidative stress (Wang, Wang, Fan, et al. 2018), while leaf shape influences light capture, heat dissipation and gas exchange. Both traits are therefore subject to parallel selective pressures and may be coordinated through shared regulatory networks, hormonal signalling pathways, or genomic linkage. At the molecular level, they may be linked through shared regulators (e.g., MYBs with roles in both pigmentation and morphogenesis), hormonal signalling pathways (auxin and jasmonate), or coordinated genomic clustering that facilitates co‐regulation (Wang, Wang, Xu, et al. 2018). Our data suggest that co‐occurrence of pigmentation and morphological variation across accessions supports the hypothesis that these traits contribute to integrated adaptive strategies.

The novelty of this study lies in the simultaneous dissection of pigmentation and morphology within a unified genomic and functional framework. We provide the first evidence that TF clusters (MYBs) and developmental regulators (BEL/LMI/TMK1/WD40/RABH1e) underpin two seemingly independent traits. By identifying TF clusters controlling leaf pigmentation, structural gene clusters controlling root pigmentation and conserved developmental regulators governing leaf morphology, we provide a comprehensive view of the genetic basis of phenotypic diversity in sweet potato. This work demonstrates how distinct regulatory modules operate in different organs while maintaining overall coordination within the genome (Figure 8).

From a practical perspective, these findings have direct implications for sweet potato breeding. The MYB clusters and structural anthocyanin genes are promising targets for enhancing pigmentation and nutritional quality, while BEL/LMI/TMK1 loci offer opportunities to optimise canopy architecture for improved photosynthesis and stress tolerance. The integrative model proposed here – where pigmentation and morphology are co‐regulated adaptive traits – provides a conceptual framework for designing sweet potato ideotypes that combine high nutritional value with agronomic performance (Figure 8).

In conclusion, this study uncovers the genetic basis of pigmentation and morphology in sweet potato and highlights their interconnected regulation. By linking metabolic specialisation with morphological adaptation, we provide new insights into plant evolution and valuable tools for crop improvement.

## 4 Materials and Methods

### 4.1 Plant Materials and Resequencing

The association panel comprised 260 sweet potato germplasm accessions grown at two locations in Hangzhou, China: Banqiao (119.76° E, 30.14° N) and Zhejiang Agriculture and Forestry University (Zhejiang A&F University; 119.72° E, 30.23° N) over two consecutive growing seasons (2021 and 2022). The four environment‐year combinations are referred to as 2021BQ, 2021NZY, 2022BQ and 2022NZY. For each accession, six clonal replicates for quantitative traits were evaluated per environment. Phenotypic measurements for 43 traits were conducted according to the standardised protocols described in Descriptors for Sweet potato Germplasm Resources (NY/T 2939‐2016), issued by the National Public Service Platform for Standards Information. For quantitative traits, integrated phenotypic values across environments were calculated as the mean of all replicates, whereas for qualitative traits, the modal value was used (Table S1).

In this study, three kinds of accessions were used for functional verification, such as RNA‐seq, qRT‐PCR analysis, VIGS, etc. Among these accessions, Mian8, Xu37, WA4921, Longshu14, QN16, Zhan18, Xu32, QN18, Xiang203, WS4723, Xu37, GS6, Long14 and XCG are from the 260 accessions and provided by Zhejiang A&F University, while XuZi201, XuZi5, Xu27, XT242, ZY, ZS, ZYQ, ZSJN and HJD are preserved in our lab, and Xu22, Xu18, HM and XuZi8 are part of the 260 accessions but preserved in our lab.

Fresh leaves of these germplasm resources were sampled, and genomic DNA was isolated using DNA secure Plant Kit (TIANGEN, Beijing, China). About 500 ng of qualified DNA for each sample was collected and constructed the library according to Plus DNA Library Prep Kit for MGI V2 (#NDM627). A qualified library was used for sequencing on the DNBSEQ‐T7 platform by MGI. The insert was 350, and 150 bp paired‐end sequencing was generated. For RNA‐seq analysis, young unexpanded leaves and tuberous root flesh were collected from 5 month old plants representing 15 sweet potato accessions: Mian8, XuZi8, WA4921, Xu37, QN16, Xu32, XuZi201, XuZi5, ZYQ, ZSJN, Xu18, Xu27, QN18, Xiang203 and WS4723. All samples were harvested at the same developmental stage under uniform growth conditions to minimise variation arising from developmental or environmental differences.

### 4.2 Correlation and Broad‐Sense Heritability (*H* ^2^) Analysis

Phenotypic data collected from four independent environments (2 years × 2 locations: 2021BQ, 2021NZY, 2022BQ, 2022NZY) were processed as follows. For quantitative traits, phenotypic values were averaged across clonal replicates within each environment, whereas qualitative traits were represented by their modal values. Pairwise correlations of phenotypic values across environments were calculated using Pearson's correlation coefficient in *R* to assess environmental consistency. Statistical significance of correlations was evaluated at thresholds of *p* ≤ 0.05, *p* ≤ 0.01, *p* ≤ 0.001, and correlation patterns were visualised using heatmaps with corresponding significance annotations.

To evaluate relationships among traits, correlation analyses were conducted using Spearman's rank correlation with the rcorr() function in the R package Hmisc, providing both correlation coefficients (*r* values) and associated *p*‐values.

Broad‐sense heritability (*H* ^2^) for both quantitative and qualitative traits was estimated using linear mixed models (LMMs) implemented in the R package lme4. In these models, genotype was treated as a random effect, with year, location and their interactions included to account for environmental variation. Variance components were extracted from the fitted models and *H* ^2^ was calculated as the ratio of genetic variance to total phenotypic variance. Traits lacking sufficient data for reliable variance estimation were excluded from heritability analysis.

### 4.3 Reads Alignment, Variant Calling and Annotation

All raw resequencing reads were filtered to remove adapters and low‐quality bases using fastp (v0.20.0) and then all clean reads for each sample were mapped to the reference genome of Taizhong6 (Yang et al. 2017) by BWA with default parameters. Samtools was used to remove low‐mapping quality reads (MQ < 30) and the alignments were sorted according to mapping coordinates. PCR duplications were removed using Picard 2.24.0 (https://broadinstitute.github.io/picard/). HaplotypeCaller and CombineGVCFs modules of GATK4.0 were used to call SNPs and InDels, and the variant filtration module was selected to filter low‐quality variants with the following criteria: for SNP filtering, QUAL < 40, QD < 2.0, FS > 60.0, MQ < 40.0, SOR > 3.0, MQRankSum < −12.5 and ReadPosRankSum < −8.0; for InDel filtering, QUAL < 40, QD < 2.0, FS > 200.0, MQ < 40.0, MQRankSum < −12.5 and ReadPosRankSum < −20.0 (DePristo et al. 2011). For GWAS, the raw SNP set was further filtered using Vcftools with the following parameters: ‐‐max‐missing 0.2 ‐‐maf 0.05 ‐‐minDP 4 ‐‐hwe 1e‐5 and finally filtered using PLINK by LD as following parameters: ‐‐biallelic‐only ‐‐indep‐pairwise 50 5 0.5 (Purcell et al. 2007). The output SNP set was used to conduct GWAS. The genetic variants were annotated using ANNOVAR (Wang et al. 2010).

### 4.4 Phylogenetic Tree Construction, PCA, Population Structure and LD Analysis

ADMIXTURE (Alexander et al. 2009) was used to infer the optimum number of clusters (*K*) among all accessions. *K* values from 2 to 10 were examined and the cross‐validation error was calculated to identify the most likely number of clusters. A PCA was performed using EIGENSOFT (Patterson et al. 2006). To infer relationships among accessions, a maximum likelihood tree was constructed based on fourfold degenerate sites in the 260 sweet potato accessions. SNPs were extracted and compared with the 11 433 913 four‐fold degenerate sites identified in the genome using iTools (20180520) (Dinov et al. 2008). SNPs from each individual were merged into one file and aligned using mafft (version 7.407) (Katoh and Standley 2013), then low‐quality regions were trimmed with trimAl (version 1.4.rev22) (Capella‐Gutiérrez et al. 2009). The final 236 923 fourfold degenerate sites were then used to construct maximum likelihood unrooted tree using IQ‐TREE (version 1.6.12) with the parameters ‐alrt 1000 ‐bb 1000 (ultrafast bootstrap) (Nguyen et al. 2015).

Pairwise linkage disequilibrium (LD) between SNPs was calculated separately for each population using PopLDdecay (Zhang et al. 2019). For each chromosome, genotype data were used to compute the squared correlation coefficient (*r* ^2^) between all SNP pairs within a maximum physical distance of 200 kb. To summarise LD patterns across the genome, pairwise *r* ^2^ values were grouped into distance bins, and the mean *r* ^2^ was plotted against physical distance to visualise LD decay. The LD decay distance was defined as the physical distance at which the mean *r* ^2^ decreased to half of its maximum value, a commonly used threshold indicating moderate linkage between loci. Analyses were conducted independently for each population to capture population‐specific LD patterns.

### 4.5 Haplotype, Population Fixation Index (*Fst*) and Nucleotide Diversity (π) Analysis

To construct haplotype heatmaps for different phenotypic groups, we selected accessions exhibiting extreme trait values. For leaf colour classification, 18 accessions with high pigmentation scores (≥ 5) for abaxial leaf midvein pigmentation, abaxial leaf lateral vein pigmentation, pigmentation at the base of leaf vein and petiole were assigned to the purple‐coloured group, while 31 accessions with minimal pigmentation (score = 1) were classified as the green‐coloured group.

For tuberous root colour classification, the white‐fleshed tuberous included 16 accessions with main colour of root cuticle score < 8, cortical flesh colour = 1, main flesh colour = 1 and secondary colour of tuberous flesh ≤ 1. In contrast, the purple‐fleshed tuberous included 19 accessions exhibiting all the above‐mentioned phenotypic scores ≥ 9.

For leaf shape classification, the lobed‐leaf group was defined by 37 accessions with shape of immature and mature leaf score = 6, sinus depth of immature and mature leaf ≥ 3, lobe number of immature and mature leaf ≥ 2 and marginal serration type of immature and mature leaf ≥ 2. The entire leaf group comprised 64 accessions that scored below these thresholds.

After defining the groups, we combined the selected accessions and re‐ordered them in the original VCF file using vcftools. The phased haplotypes were inferred using Beagle, and haplotype heatmaps were generated using R scripts with the vcfR package.

Genetic differentiation among phenotypic groups was assessed using VCFtools (v0.1.17). Pairwise population differentiation was quantified by calculating Weir and Cockerham's *Fst* values between contrasting phenotypic groups, including purple‐ versus green‐leaf accessions, white‐ versus purple‐fleshed storage root accessions and lobed‐ versus entire‐leaf accessions. A sliding window approach was applied with a window size of 100 kb and a step size of 10 kb to identify genomic regions exhibiting elevated allele frequency divergence.

To complement the *Fst* analysis, nucleotide diversity (π) was calculated independently for each phenotypic group using the same sliding window parameters (100 kb window size and 10 kb step size). This analysis enabled genome‐wide characterisation of within‐group genetic diversity and facilitated comparison of π variation between phenotypic groups, particularly in regions showing elevated genetic differentiation.

### 4.6 Gene Flow Analysis

Population structure and historical gene flow were inferred using TREEMIX (v1.13) based on allele frequency data from the five inferred populations (Pickrell and Pritchard 2012). A maximum likelihood population tree was constructed without specifying a root, and the block size was set to 10 000 SNPs to account for linkage among adjacent markers. The number of migration edges (*m*) was tested from 1 to 5 to evaluate alternative models of gene flow. The optimal number of migration events was determined using the OptM algorithm, which identifies the point of diminishing returns in model likelihood improvement. Model fit was further assessed by examining residual covariance matrices, visualised as heatmaps, to identify population pairs exhibiting excess covariance indicative of gene flow.

To further quantify introgression, D‐statistics (ABBA‐BABA tests) were calculated using Dsuite v0.5, allowing quantification of gene flow among population trios (Malinsky et al. 2021). The extent of introgression at specific loci was estimated based on the relative frequency of ABBA and BABA site patterns, and results were visualised using an f‐branch diagram generated with accompanying dtools.py script.

### 4.7 GWAS Analysis

GWAS was performed using the GEMMA software package (v0.98.1) with 4 585 655 high‐quality SNPs to identify genetic loci associated with the phenotypic traits (Zhou and Stephens 2012). The analysis incorporated linear mixed models (LMM) to account for population structure and familial relatedness. The population kinship matrix and the first five PCA components were obtained by smartpca, a program from the EIGENSOFT 8.0.0 software package. The association significance threshold was set as 1e‐06. Candidate genes were annotated based on the sweet potato reference genome using ANNOVAR.

Phenotypic values integrated across two ‐years and two locations were used as the primary input. In parallel, environment‐specific datasets were analysed in parallel for three representative trait categories (leaf colouration, tuberous root pigmentation and leaf shape) to assess the reproducibility and environmental effects. Significant SNPs were mapped to the sweet potato reference genome to identify and annotate candidate genes. Manhattan plots were generated using the CMplot R package, with −log_10_(*p*) values plotted across chromosomes for multiple traits simultaneously (plot.type = ‘*m*’, multraits = TRUE). Only SNPs with at least one trait *p*‐value below 1e‐04 were retained for visualisation across two years and two locations; those exceeding the significance threshold (*p* ≤ 1e‐06) were coloured distinctly and optionally highlighted. Point transparency and size were adjusted (points.alpha = 200, signal.cex = 1) to improve readability, and the legend was centrally positioned.

### 4.8 PVE Calculation

To estimate the proportion of phenotypic variance explained (PVE) by each SNP, we applied a standard quantitative genetic model. For each trait, the total phenotypic variance (Var_y) was calculated from the raw phenotypic values across all accessions. The PVE for each SNP was then computed using its additive effect size (*β*) and allele frequency (AF) obtained from GEMMA association analyses. Specifically, PVE was calculated as:

$$\mathrm{PVE}=2\times \mathrm{MAF}\times \left(1-\mathrm{MAF}\right)\times \beta^{²}/\mathrm{Var}\_y$$

where MAF represents the minor allele frequency and *β* is the additive effect estimated under the univariate linear mixed model. This formulation quantifies the proportion of phenotypic variance attributable to each SNP given its allele frequency and estimated effect size. The resulting PVE values are reported for all SNPs surpassing the genome‐wide significance threshold (*p* ≤ 1 × 10^−6^).

### 4.9 Kinship Matrix and Genomic Inflation Factor (*λ*) Estimation

To account for relatedness among individuals in the GWAS panel, a standardised kinship matrix was calculated using GEMMA (−gk 2) based on 4 585 655 high‐quality SNPs. The resulting kinship matrix (*K*) quantifies pairwise genetic relatedness among all 260 accessions. Pairwise kinship coefficients were extracted from the upper triangle of the matrix and summarised to characterise the overall distribution of relatedness, including the maximum value, mean and proportion of pairwise comparisons exceeding 0.5.

To assess potential inflation of GWAS test statistics caused by population structure or cryptic relatedness, genomic inflation factors (*λ*) were calculated for each of the 43 traits. SNP‐level *p*‐values generated by GEMMA association analyses (−assoc output, Wald test *p*‐values) were converted to chi‐square statistics with one degree of freedom using the transformation: $\begin{array}{l} \chi^{²}=\text{qchisq}\left(1-p,\mathrm{df}=1\right) \end{array}$. The genomic inflation factor was then computed as the ratio of the median observed chi‐square statistic to the theoretical median under the null hypothesis (0.455 for df = 1): *λ* = median ($\chi^{²}$_obs_)/0.455. This procedure was applied to all traits and summary statistics (the mean, standard deviation and distribution) were calculated to evaluate overall inflation across the dataset. A *λ* value close to 1 indicates minimal test statistic inflation and confirms effective correction of population structure and relatedness in the GWAS model.

### 4.10 Colocalisation Analysis

Pairwise genetic colocalisation analyses were performed using the coloc R package based on GWAS summary statistics. For each trait, the top three lead SNPs were selected according to GWAS significance, and genomic loci were defined as ±100 kb windows centred on each lead SNP. Colocalisation analysis was conducted for trait pairs with at least 50 overlapping SNPs within the corresponding loci. Posterior probabilities for five alternative hypotheses were estimated using the approximate Bayes factor model, and loci with a posterior probability for a shared causal variant (PP.H4) ≥ 0.8 were considered to show strong evidence of genetic colocalisation. When multiple loci were tested for a given trait pair, the locus with the highest PP.H4 value was retained as the representative colocalisation signal.

### 4.11 Structural Variation Analysis of the GWAS‐Associated Region and MYB Copy Number Variation Analysis

To determine whether large‐scale structural variations contribute to the extended GWAS association signal on chromosome 5, comparative genomic analyses were performed using three purple‐fleshed sweet potato cultivars (Ayamurasaki, XuZi8 and WA4921). These accessions were deliberately selected to control for storage‐root pigmentation (all purple‐fleshed) while capturing variation in aerial pigmentation: Ayamurasaki and WA4921 exhibit purple immature leaves, whereas XuZi8 displays green immature leaves (Figure S12B). This contrast enables discrimination between structural variants associated with root pigmentation per se and those potentially linked to leaf pigmentation. High‐molecular‐weight genomic DNA was extracted from apical immature leaves and sequenced on the PacBio Revio platform (Wuhan Benagen Technology Co. Ltd., Wuhan), generating HiFi reads totalling 43.16, 45.22 and 28.65 Gb with read N50 values of 14.4, 16.2 and 16.4 kb, respectively (Figure S12A). *De novo* genome assemblies were generated using hifiasm (v0.19.9) (Cheng et al. 2021), producing high‐quality contig‐level assemblies with N50 values ranging from 2.18 to 6.55 Mb.

Chromosomal correspondence between reference genomes was established by aligning the Tanzania reference genome (v3) (Wu et al. 2025) to the Taizhong6 reference genome using Minimap2 (Li 2018), confirming that Tanzania chromosome 12 is syntenic with Taizhong6 chromosome 5. To assess structural conservation within the GWAS‐associated interval (~19–30 Mb) on Taizhong6 chromosome 5, contigs longer than 1 Mb with alignment start positions exceeding 19 Mb were extracted and aligned to Tanzania chromosome 12. Collinearity and structural relationships across subgenomes were examined using dot plot visualisations generated with pafCoordsDotPlotly.R. Structural variation analyses focused on detecting large‐scale genomic rearrangements, including inversions and translocations, with particular attention to previously reported inversion regions.

To assess copy number variation (CNV) among MYB genes, comparative genomic analyses were conducted using three purple‐fleshed sweet potato genomes (Ayamurasaki, XuZi8 and WA4921) together with the published yellow‐fleshed Tanzania reference genome. Candidate MYB genes identified from the Taizhong6 reference genome were first aligned to the Tanzania genome using BLAST to validate chromosomal localisation and confirm the integrity of the MYB cluster. Subsequently, MYB gene sequences were aligned to the assemblies of the three purple‐fleshed cultivars to identify corresponding genomic contigs. Gene prediction was performed on these contigs with BRAKER v1.9 with default parameters (Hoff et al. 2016) to annotate gene models. Collinearity and local gene organisation were examined using JCVI (Tang et al. 2008) to compare MYB cluster structure across subgenomes and accessions. MYB copy numbers were then enumerated per contig to quantify CNV among accessions and subgenomes. This approach allowed direct comparison of MYB gene dosage and cluster organisation among cultivars.

### 4.12 RNA Isolation, Transcriptome Sequencing and qRT‐PCR

Total RNA was extracted from leaves using RNAprep Pure Plant Plus kit (DP441; TIANGEN, Beijing, China) according to the manufacturer's instructions. After DNase treatment, reverse transcription was performed using ReverTra Ace qPCR RT Master Mix (Code No. FSQ‐201; TOYOBO, Osaka, Japan). Then the cDNA was used as the template for qRT‐PCR, which was performed using Hieff qPCR SYBR Green Master Mix (11203ES08; Yeasen Biotechnology, Shanghai, China) on the CFX96 Touch Real‐Time PCR Detection System with three biological replicates. Gene expression level was normalised to the expression of actin gene (*g27691*) and calculated for each sample from the $2^{-∆∆C_{t}}$ method. The primers used in qRT‐PCR are listed in Table S14.

RNA‐seq libraries were prepared using the Illumina TruSeq RNA Library Prep Kit and sequenced on the Illumina HiSeq 4000 platform. Raw reads were processed using fastp (version 0.36) for adapter removal and quality control. Clean reads were aligned to the sweet potato reference genome Taizhong6 using HISAT2 (version 2.1.0). Gene expression levels were quantified using HTseq_count (version 0.13.5) and differential expression analysis was performed using DESeq2.

### 4.13 Anthocyanin Extraction and Quantification

Anthocyanins were extracted from sweet potato leaves following a modified method based on previous protocols. Fresh tissue samples were first homogenised in liquid nitrogen using a mortar and pestle, followed by the addition of 1 mL of 80% methanol (v/v) containing 2% formic acid (HCCOH) as the solvent. The ~100 g homogenate was incubated at room temperature for 30 min with gentle shaking to ensure complete extraction in the dark. After incubation, the mixture was centrifuged at 12 000× ***g*** for 15 min at 4°C to remove debris. The supernatant was collected, and the anthocyanin content was quantified using a spectrophotometer by measuring absorbance at 530 nm (for anthocyanin). For quantitative analysis, the anthocyanin content was calculated using a formula *y* = 0.1594*x* − 0.0001 (mg/mL) and the results were expressed as mg of anthocyanin per gram of fresh weight (mg/g FW).

### 4.14 Construction Vector of VIGS and VIGS Infection

The genome and transcript database of *Ipomoea batatas* cultivar Taizhong6 was used as the reference. Vector construction and VIGS infection were performed as described by Zhang et al. (2024) with slight modifications. A 513 bp consensus fragment of the MYB gene was amplified using primers myb‐vigs‐LP and myb‐vigs‐RP (Table S14) and cloned into the SBG51‐based VIGS vector via homologous recombination (C115‐02‐AA, Vazyme). The recombinant constructs were subsequently introduced into *Agrobacterium tumefaciens* strain AGL1. Overnight cultures were pelleted, resuspended in induction medium and finally adjusted in inoculation buffer.

For co‐inoculation, sweet potato cuttings with 6–8 leaves were soaked in a mixed suspension of the SPLCV infectious clone and the recombinant VIGS constructs in the dark, briefly recovered in water and then transferred to soil for further growth. For mock controls, plants were inoculated with the SPLCV infectious clone together with the empty SBG51 VIGS vector. Plants were monitored for VIGS phenotypes; gene silencing efficiency and viral infection efficiency were assessed at 10–14 days post inoculation.

### 4.15 Leaf Dissection Index (LDI) Measurement

Leaf shape complexity was quantified using the leaf dissection index (LDI), calculated as *P* ^2^/(4π*A*), where *P* represents the leaf perimeter and *A* represents the leaf area. Higher LDI values indicate greater leaf margin dissection and increased morphological complexity (Zheng et al. 2016).

### 4.16 Homologous Gene Identification

Homologous MYB proteins in sweet potato were identified by performing BLASTP searches against the Taizhong6 protein database, using Arabidopsis MYB11, MYB12, MYB111, MYB4, PAP1, PAP2, TT2, MYB32 and MYB5 as query sequences with the parameter ‐max_target_seqs 6. Similarly, the BEL1‐like homeodomain gene (g29974) was used as a query in a BLASTP search against the Arabidopsis protein database (−max_target_seqs 1) to identify putative homologues. Using these eight MYB genes as query sequences, a total of 43 highly homologous MYB genes were identified in the sweet potato genome (*E*‐value ≤ 1e‐40). All candidate protein sequences were subjected to multiple sequence alignment using MAFFT, and the alignments were visualised in SnapGene. On the basis of the aligned sequences, phylogenetic relationships were inferred by constructing phylogenetic trees using previously described methods.

### 4.17 Luciferase Complementation Assay in *Nicotiana benthamiana*

Reporter constructs were generated by inserting ~2 kb promoters of *IbMYB1*, *IbMYB2*, or *IbMYB3* from cultivar Ayamurasaki and WA4921 into the KpnI and HindIII sites of the pGreenII‐0800 vector. Effector constructs (35S:*IbMYB1*, 35S:*IbMYB2* and 35S:*IbMYB3*) were generated by cloning the corresponding coding sequences into pRI101 vector using SalI and EcoRI restriction sites. Empty vectors were included as negative controls. All primers used for vector construction are listed in Table S14.

*Agrobacterium tumefaciens* GV3101 carrying the reporter and effector constructs was co‐infiltrated into 5‐week‐old, fully expanded *Nicotiana benthamiana* leaves using a needleless syringe. After 48 h under 16 h light/8 h dark, luciferin substrate (0.94 mM luciferin) was then infiltrated into the leaves, and luminescence signals were captured using a Tanon‐5200 imaging system. Each experiment was performed with at least three independent biological replicates.

Quantitative analysis of luminescence intensity was performed using ImageJ. Relative luminescence was compared among treatments. Statistical significance was determined using one‐way ANOVA followed by Tukey's HSD test. Data are presented as mean ± standard error of the mean (SEM), with individual biological replicates indicated.

### 4.18 CRISPR/Cas9 Vector Construction and Stable Transformation

Guide RNAs (gRNAs) targeting the *g26165* gene were designed using a locally installed CRISPR design program, with the full‐length coding sequence of *g26165* used as the reference. Candidate 20‐bp target sites were selected based on the presence of a 5′‐NGG‐3′ protospacer adjacent motif (PAM) required for SpCas9 recognition and cleavage (Liu et al. 2015).

Complementary oligonucleotides corresponding to the selected target sequence were synthesised, annealed and ligated into the pSGR‐Cas9‐At vector. The recombinant plasmid was verified by Sanger sequencing before introduction into *Agrobacterium tumefaciens* strain LBA4404. Stable genetic transformation was performed using embryogenic calli derived from the sweet potato cultivar ‘Xushu22’. *Agrobacterium*‐mediated transformation, selection and plant regeneration were conducted according to previously established protocol (Yang et al. 2011). Regenerated plantlets were screened under selective conditions, and transgenic lines were confirmed by molecular analysis. To validate genome editing events, genomic regions flanking the target sites were amplified using primers designed approximately 100 bp upstream and downstream of the gRNA target sequence (g26165‐snp‐HiTom‐F and g26165‐snp‐HiTom‐R). Amplicons were subjected to high‐throughput sequencing using high‐throughput sequencing (Liu et al. 2019).

### 4.19 Vector Construction and Arabidopsis Transformation

To generate the *g17106* expression construct, the CaMV 35S promoter in the pCAMBIA1300 vector was removed by digestion with NcoI and SacI. A genomic fragment containing the native promoter and coding sequence of *g17106* was amplified from the sweet potato line WA4921 using primers g17106‐2000Pro‐F (located within the endogenous promoter region) and g17106‐GFP‐RP (located downstream of the coding sequence). The amplified fragment was subsequently PCR‐amplified using primers g17106‐arm‐Pro2000F and g17106‐arm‐R to introduce homologous recombination arms. The resulting fragment was cloned into the modified pCAMBIA1300 vector via homologous recombination and fused in‐frame with GFP to generate a C‐terminal GFP fusion construct.

For *g29859*, the coding sequence was amplified from the WA4921 genomic DNA using primers g29859‐GFP‐FP and g29859‐GFP‐RP, followed by a second PCR with primers g29859‐GFP‐arm‐FP and g29859‐GFP‐arm‐RP to introduce homologous arms. The amplified fragment was then cloned into the pCAMBIA1300 vector via homologous recombination to generate a GFP fusion construct. The full‐length coding sequence of *g29974* was synthesised and cloned into the pCAMBIA1302 vector to generate a GFP fusion construct.

All plasmids were verified by Sanger sequencing prior to transformation. The confirmed constructs were introduced into *Agrobacterium tumefaciens* strain GV3101 and subsequently transformed into *Arabidopsis thaliana* using the floral dip method (Clough and Bent 1998). Transgenic plants were selected on hygromycin‐containing medium, and successful transformants were confirmed by molecular analysis. Primers used for vector construction are listed in Table S14. After surface sterilization with 3% NaClO and sowing on 1/2 MS medium, Arabidopsis seeds were stratified at 4°C for 3 days. Seedlings were then cultivated at 22°C under a 16 h light/8 h dark photoperiod for 2 weeks and finally transferred to soil for continued growth.

## Author Contributions

D.A. conducted the data analysis and drafted the manuscript. T.S. and D.A. performed the laboratory experiments. S.W. carried out the fieldwork for phenotypic evaluation of the population. Y.L. conducted sequence analysis of the MYB clusters and deposited the raw sequencing data in the CNCB database. W.F. and Y.M. assisted with the sweet potato callus transformation. M.Y. confirmed GWAS signals for leaf shape through analysis of another independent population. X.W. performed the analysis on part of the data. X.X. and Z.L. guided fieldwork for phenotypic evaluation of the population. L.Y. provided critical suggestions, contributed to data interpretation, revised the manuscript and supervised the overall study. J.Y. provided guidance on figure and manuscript revision. G.L. contributed the plant materials and designed the study. H.W. designed the study and supervised manuscript revision.

## Conflicts of Interest

The authors declare no conflicts of interest.

## Supporting information


**Data S1:** pbi70636‐sup‐0001‐Figures.doc.

**Data S2:** pbi70636‐sup‐0002‐Tables.xlsx.

## Data Availability

The raw DNA and RNA sequence data generated in this study have been deposited in the China National Center for Bioinformation (CNCB) database under accession numbers CRA028761 and CRA028815, respectively. The variant call files (VCF) used for the GWAS analysis are accessible at CNCB with accession number GVM001130.
