# Supplementary material for: Sweet Potato Gene Clusters Control Anthocyanin Biosynthesis and Leaf Morphology
Source: Plant Biotechnol J. 2026 Mar 24;24(7):4500–27. doi: 10.1111/pbi.70636 (PMC13278536; doi:10.1111/pbi.70636)
Supplement: Supplementary file 1 — Data S1: pbi70636‐sup‐0001‐Figures.doc. [file PBI-24-4500-s002.doc]

**Supplementary Figures: Figure S1–S29**

**
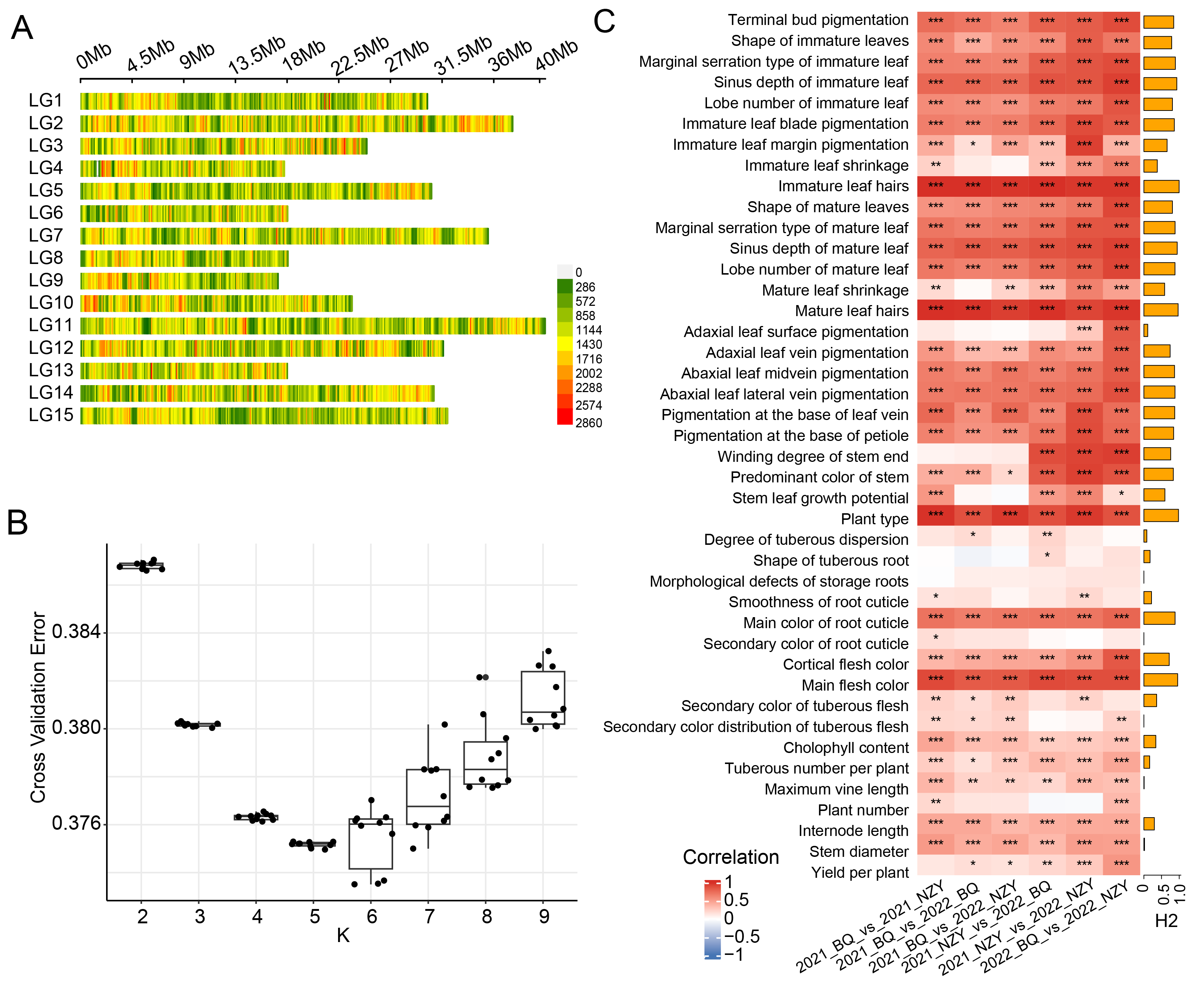
**

**Figure S1. SNP distribution, population structure, and trait correlations across environments.**

**(A)** Distribution of 4,585,655 SNPs across 15 chromosomes, illustrating the genomic variation within the sweet potato population. **(B)** Cross-validation (CV) error curve for Admixture analysis with K = 2–9. The lowest CV error occurs at K = 5, supporting the classification of accessions into 5 genetic groups. **(C)** Correlations of traits across four independent environments. Pairwise correlations among 43 agronomic traits measured across four environments (2021BQ, 2021NZY, 2022BQ, 2022NZY) were calculated using Pearson correlation to assess reproducibility and environmental effects, with statistical significance indicated by asterisks (**p* ≤ 0.05; ***p* ≤ 0.01; ****p* ≤ 0.001). Color intensity reflects the strength and direction of correlations (blue: negative; red: positive). Broad-sense heritability (H²) for each trait was estimated using linear mixed models, with genotype as a random effect and year, location, and their interactions as environmental factors, and is displayed as a right-side bar, where higher bars indicate greater genetic contribution to phenotypic variation.


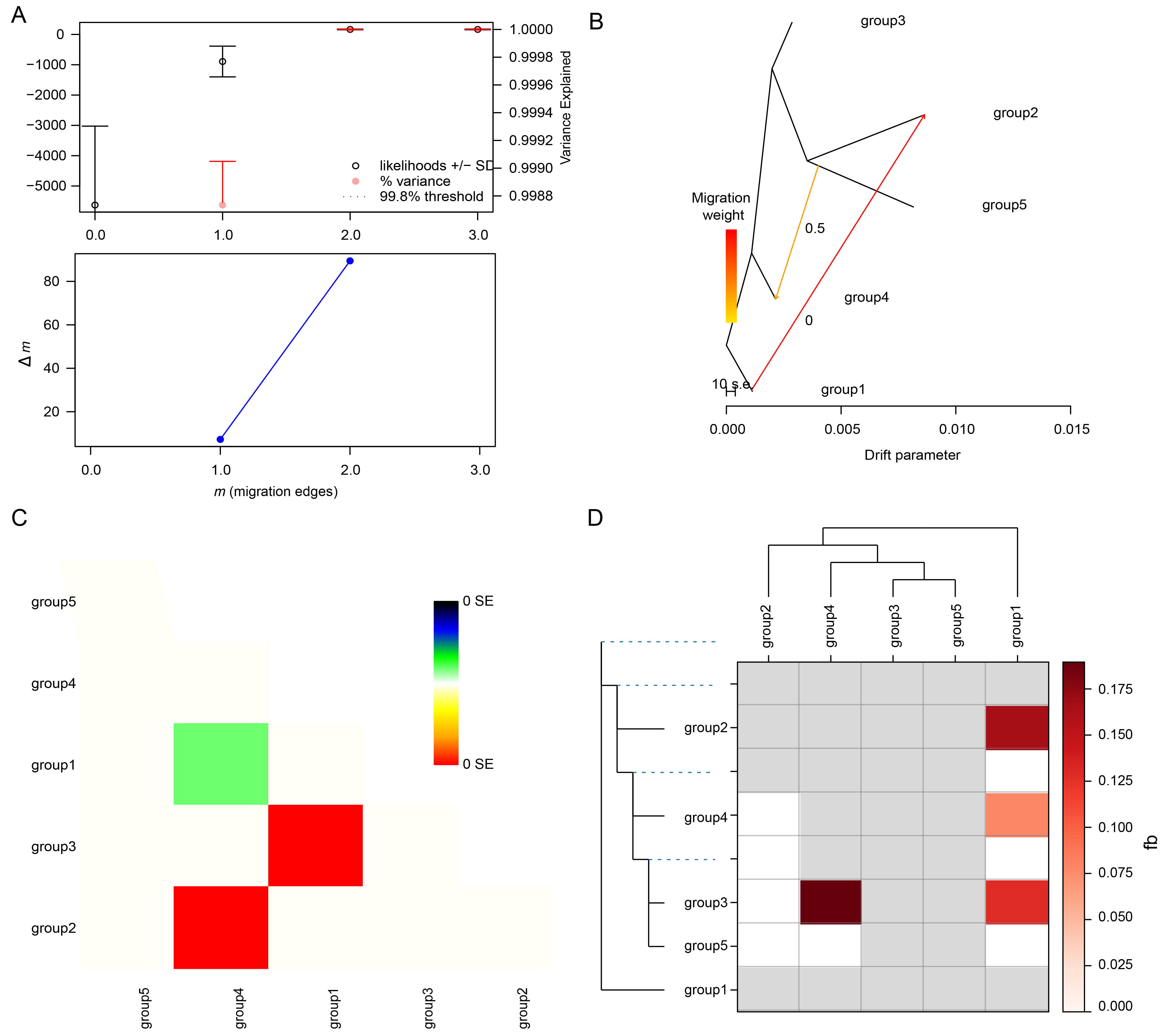


**Figure S2. Gene flow and historical admixture among the five sweet potato subpopulations.**

**(A)** Cross-validation plot from OptM showing model likelihoods for different numbers of migration edges; the minimum CV error indicates the best-fit model at m = 2. **(B)** Maximum likelihood population tree with two inferred migration edges. Solid arrows indicate the direction and relative magnitude of gene flow, showing migration from group 1 to group 2 and from group 5 to group 4. **(C)** Residual fit plot highlighting population pairs whose allele frequency covariance is not fully explained by the tree, revealing additional admixture signals. **(D)** f-branch diagram summarizing gene introgression among the five groups. Color intensity reflects the proportion of introgression, with darker shades indicating higher levels, consistent with the migration edges in **(B)**.


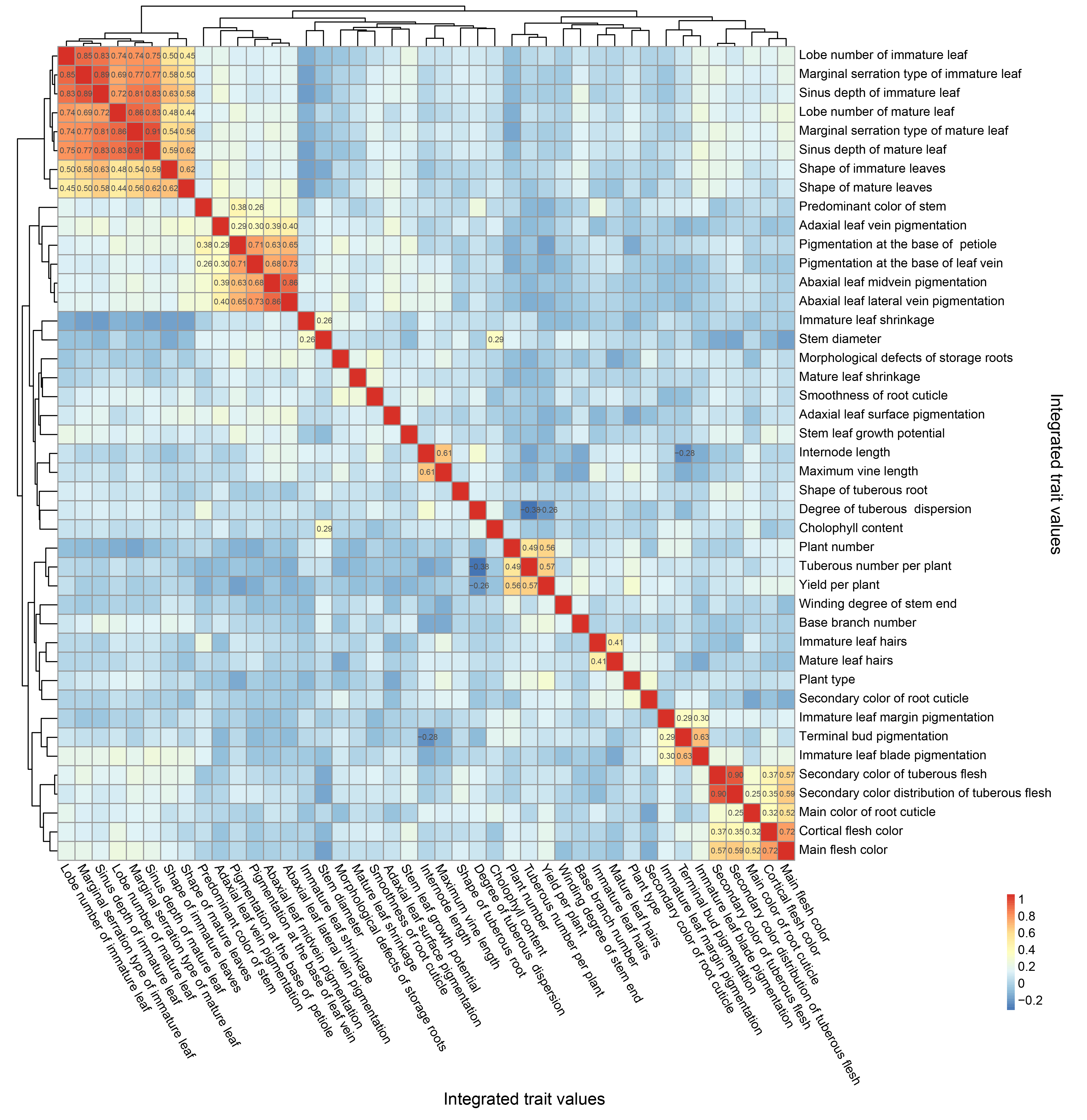


**Figure S3.** Spearman **correlation among 43 agronomic traits.**

This figure shows a correlation matrix depicting relationships among 43 agronomic traits, based on integrated trait values measured across two years and two locations. Integrated values were calculated by averaging quantitative traits and taking the mode for qualitative traits across all accessions. Trait interrelationships were assessed using Spearman’s rank correlation via the rcorr function in the R package Hmisc. Only correlations meeting the thresholds of |r| ≥ 0.25 and *p* ≤ 0.05 are numerically labeled, while non-significant correlations are omitted. Color intensity represents the strength and direction of the correlations, with positive correlations in warm colors and negative correlations in cool colors.


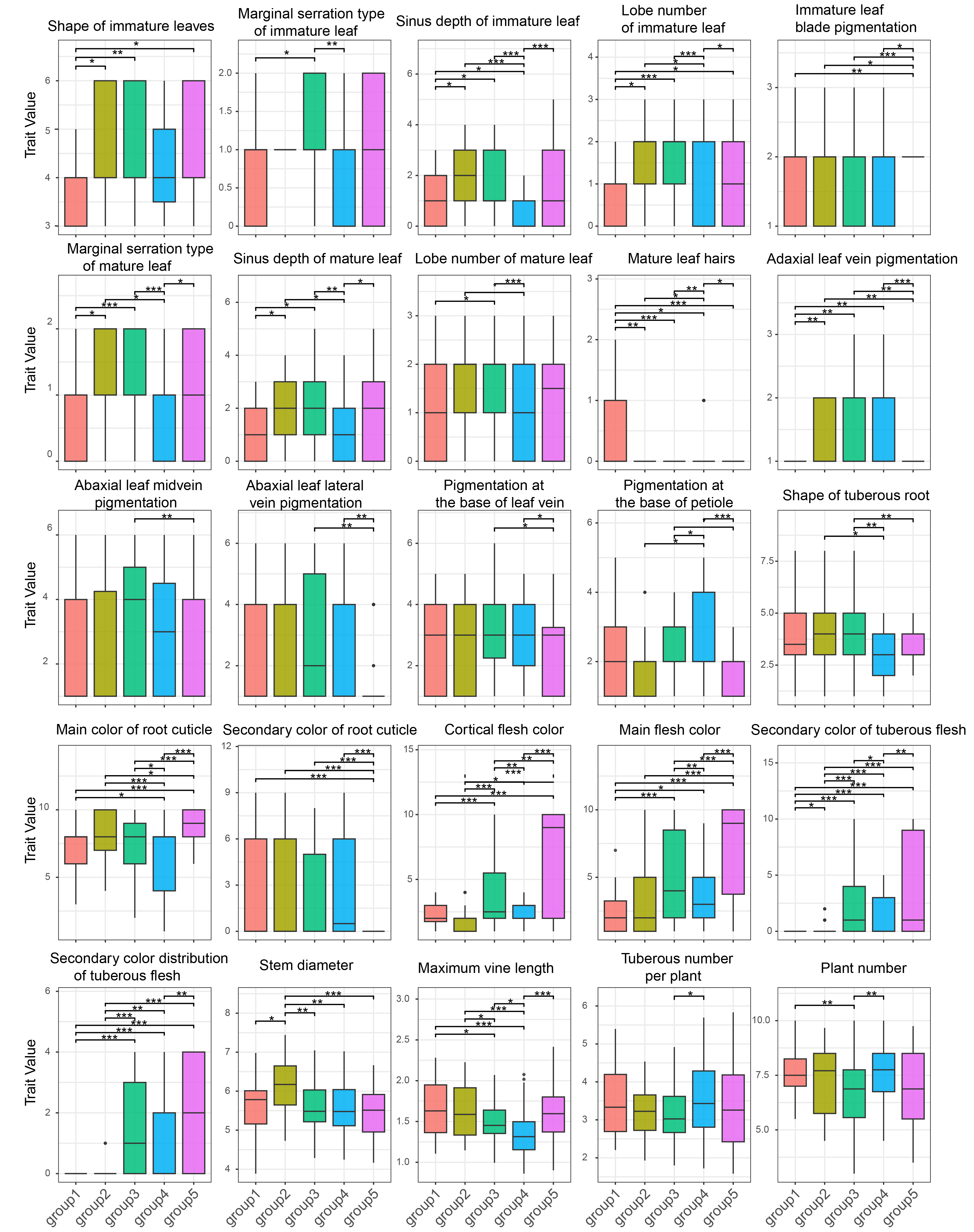


**Figure S4. Phenotypic differentiation among five sweet potato groups.**Distributions of 43 agronomic and morphological traits were compared across the five groups. After removing outliers, 25 traits exhibited significant differences among groups. Boxplots show the median, interquartile range (IQR), and full range for each trait. Statistical significance was evaluated using ANOVA followed by FDR correction, with significant differences indicated by asterisks (*FDR ≤ 0.05, **FDR ≤ 0.01, ***FDR ≤ 0.001).


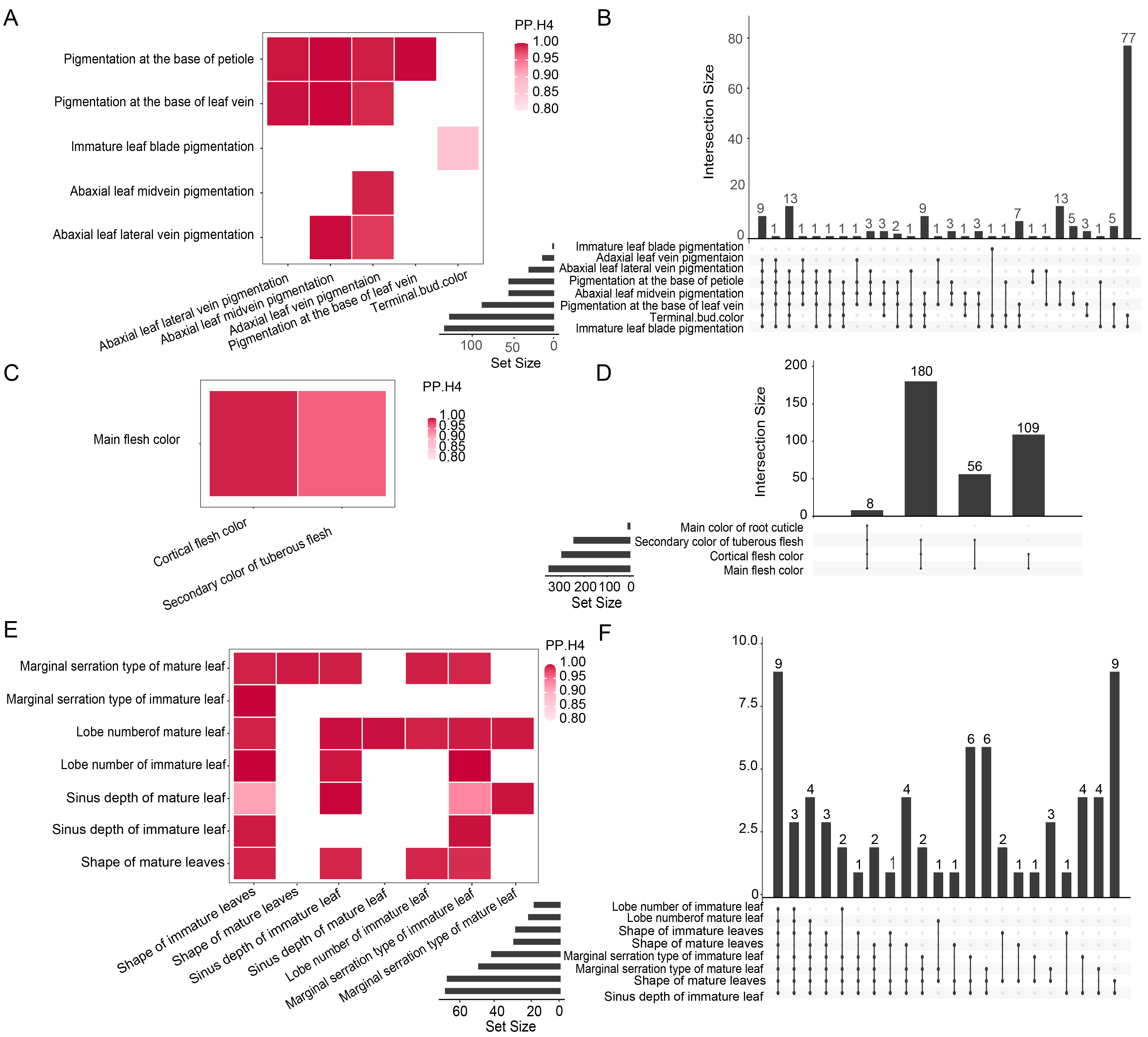


**Figure S5. Genetic colocalization and shared candidate genes among correlated traits.**
**(A, C, E)** Pairwise colocalization heatmaps based on coloc analysis for traits related to leaf coloration **(A)**, tuberous root pigmentation **(C)**, and leaf shape **(E)**. Color intensity represents the posterior probability of a shared causal variant (PP.H4), with higher values indicating stronger evidence for genetic colocalization between trait pairs. **(B, D, F)** UpSetR plots showing overlap of candidate genes identified from GWAS-significant loci among traits associated with leaf coloration **(B)**, tuberous root pigmentation **(D)**, and leaf shape **(F)**. Candidate genes were defined based on SNPs exceeding the genome-wide significance threshold (1e-06). Together, these analyses indicate that phenotypically correlated traits within each category tend to share common genetic loci, supporting partial genetic colocalization.


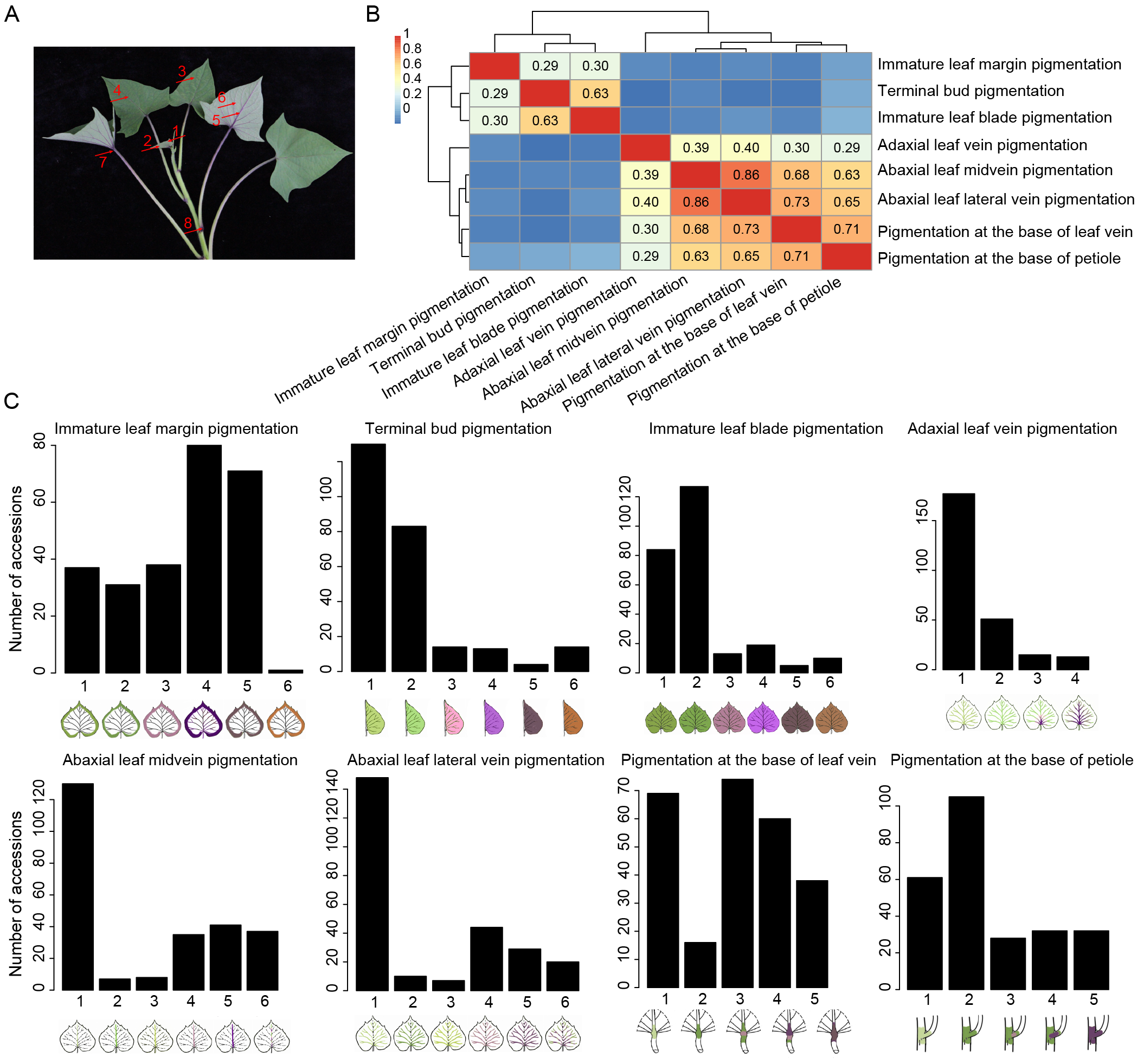


**Figure S6. Phenotypic characterization of leaf coloration traits in sweet potato.**

**(A)** Image showing the leaf coloration traits of sweet potato, with labels indicating the following traits: 1) immature leaf margin pigmentation, 2) terminal bud pigmentation, 3) immature leaf blade pigmentation, 4) adaxial leaf vein pigmentation, 5) abaxial leaf midvein pigmentation, 6) abaxial leaf lateral vein pigmentation, 7) pigmentation at the base of leaf vein, and 8) pigmentation at the base of petiole. **(B)** Spearman correlation matrix of the eight leaf coloration traits, illustrating the relationships between each trait. Correlation coefficients were calculated using Spearman’s rank correlation via the rcorr function in the R package Hmisc. Only statistically significant correlations meeting the criteria of |r| ≥ 0.25 and *p* ≤ 0.05 are numerically labeled; non-significant correlations are omitted. The matrix therefore displays only correlations that satisfy these thresholds. **(C)** Bar chart showing the statistical distribution of the vascular traits across the sweet potato population, depicting the phenotypic variation. The x-axis represents the quantified phenotypic scores, and the y-axis indicates the number of accessions.


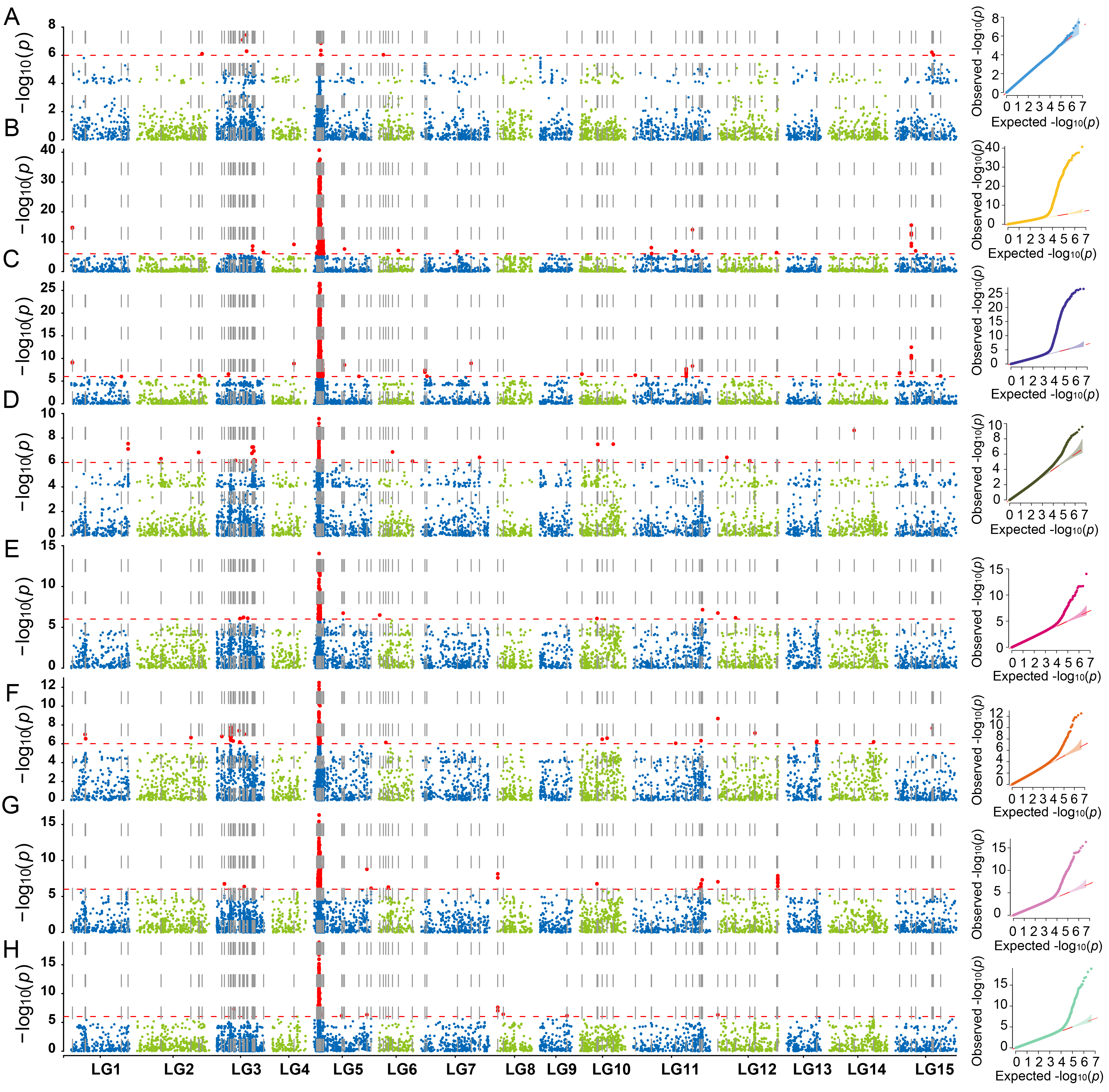


**Figure S7. GWAS identification of significant loci associated with leaf coloration.**
**(A-H)** Manhattan and Quantile-Quantile (Q-Q) plots for various traits: immature leaf margin pigmentation **(A)**, terminal bud pigmentation **(B)**, immature leaf blade pigmentation **(C)**, adaxial leaf vein pigmentation **(D)**, abaxial leaf midvein pigmentation **(E)**, abaxial leaf lateral vein pigmentation **(F)**, pigmentation at the base of leaf vein **(G)**, and pigmentation at the base of petiole **(H)**. Phenotypic integrated trait values (Table S1), obtained across two years and two locations by averaging quantitative traits and using the mode of qualitative traits for each accession, were used for GWAS. Grey lines connect SNPs at identical physical positions that exceed the significance threshold in at least one trait, providing a visual reference for comparing SNP locations across traits; these lines do not indicate statistical colocalization. Trait-specific significant SNPs (p ≤ 1e-06) are highlighted as red points. Red dashed lines represent the genome-wide significance threshold in the Manhattan plots.


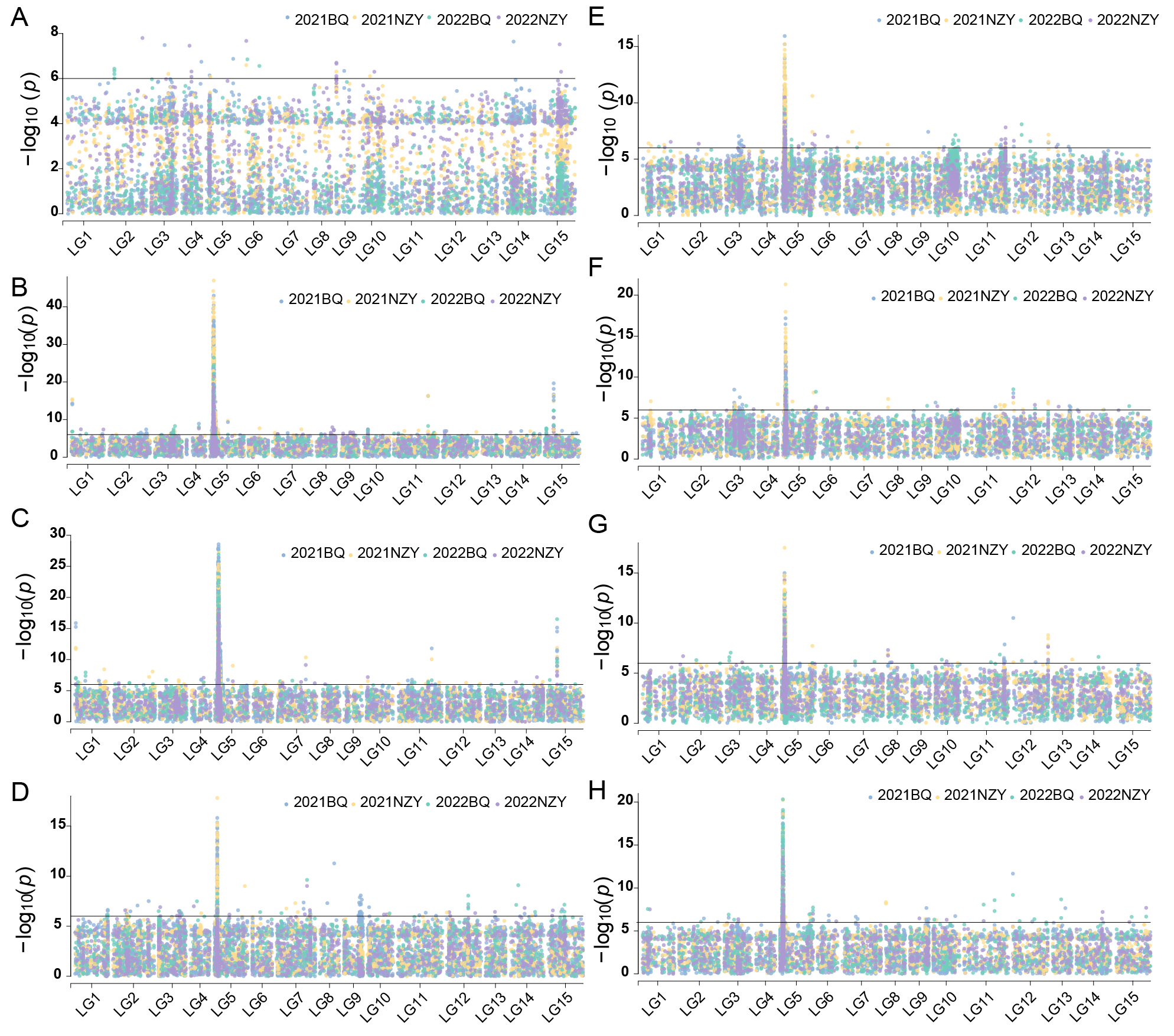


**Figure S8. GWAS identification of significant loci associated with leaf coloration across two years and two locations.**
**(A–H)** Manhattan plots for eight leaf pigmentation traits: immature leaf margin pigmentation **(A)**, terminal bud pigmentation **(B)**, immature leaf blade pigmentation **(C)**, adaxial leaf vein pigmentation **(D)**, abaxial leaf midvein pigmentation **(E)**, abaxial leaf lateral vein pigmentation **(F)**, pigmentation at the base of leaf vein **(G**), and pigmentation at the base of petiole **(H)**. Phenotypic values measured in each of the four environments (2021BQ, 2021NZY, 2022BQ, 2022NZY) were used for GWAS. The black horizontal lines indicate the genome-wide significance threshold (1e-06).


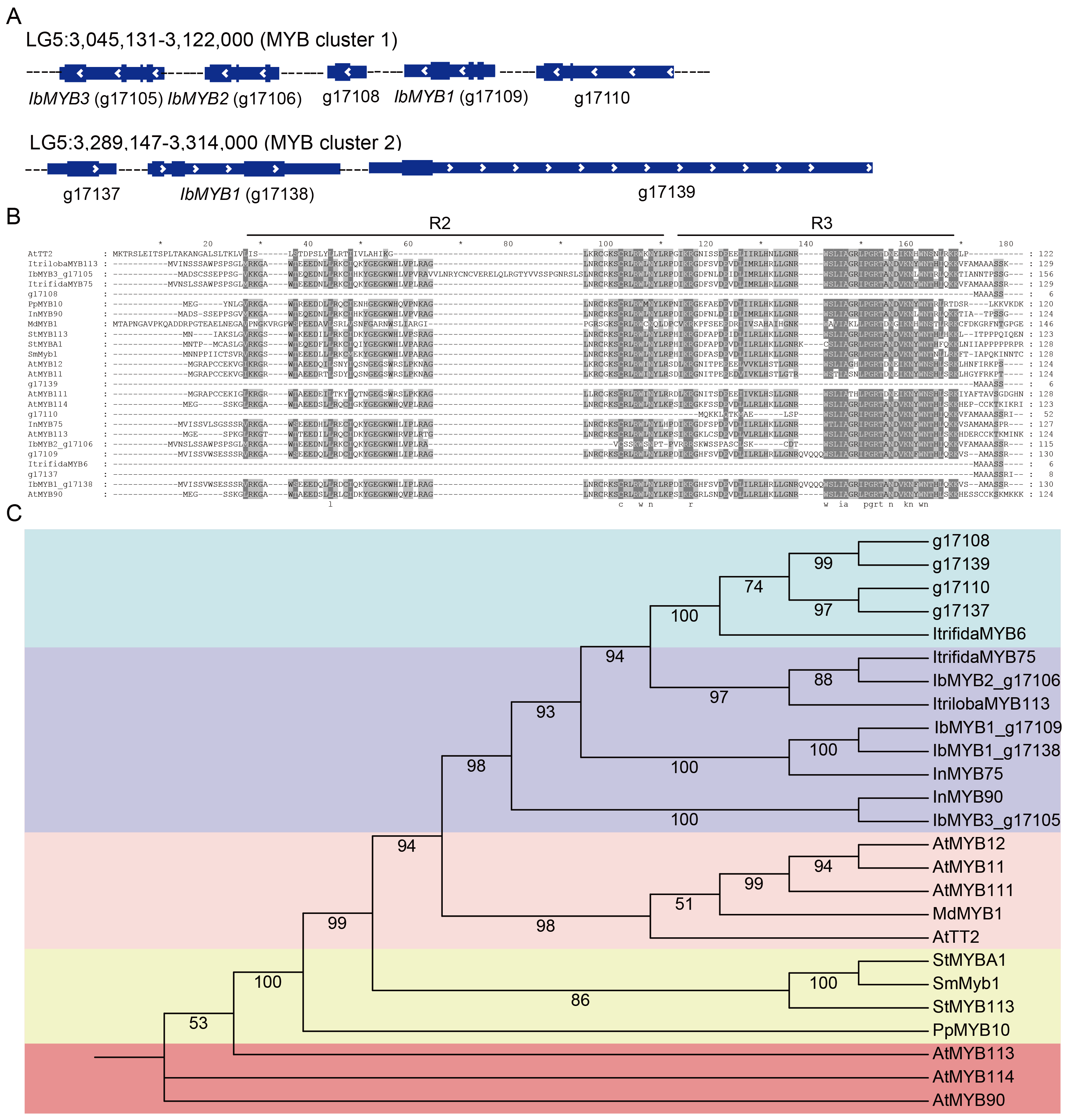


**Figure S9. Analysis of Two MYB Clusters.**
**(A)** Gene structures of the two MYB cluster genes. **(B)** Amino acid alignment of the MYB proteins with Arabidopsis MYB homologs, with conserved R2R3 MYB domains indicated by horizontal lines. **(C)** Phylogenetic tree analysis of the MYB proteins and their homologs.


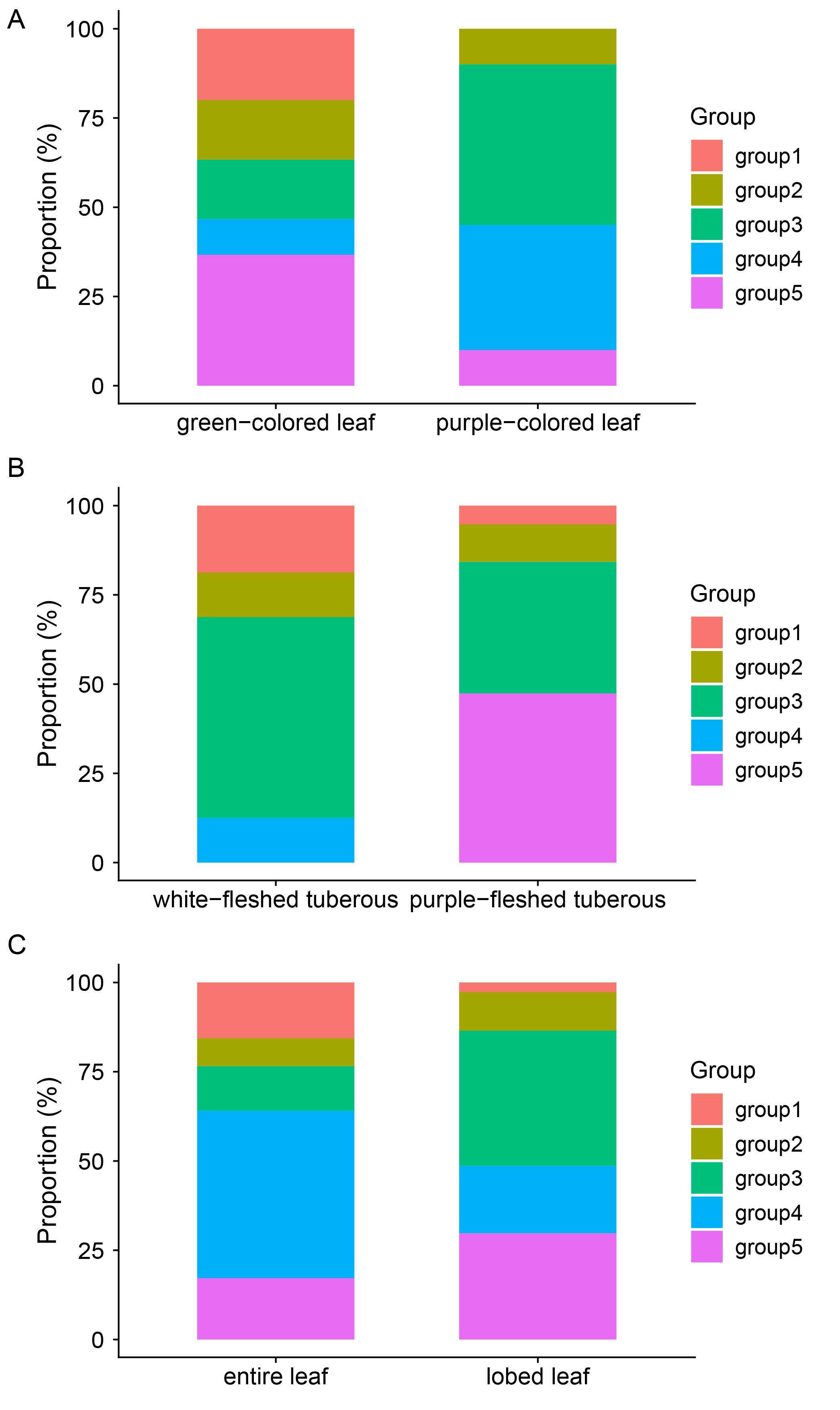


**Figure S10. Proportional representation of phenotype-defined extreme groups within the five genetic subpopulations (groups 1**–**5)**.

(A) Distribution of green- and purple-colored leaf accessions. (B) Distribution of white- and purple-fleshed tuberous root accessions. (C) Distribution of entire- and lobed-leaf accessions. These phenotype-based groups were selected for extreme trait values across the entire diversity panel and are not intended to correspond directly to the previously defined genetic groups or represent discrete evolutionary lineages. Instead, this classification highlights the distribution of extreme phenotypes across the genetic backgrounds. The criteria for phenotype selection are detailed in the Materials and Methods section.


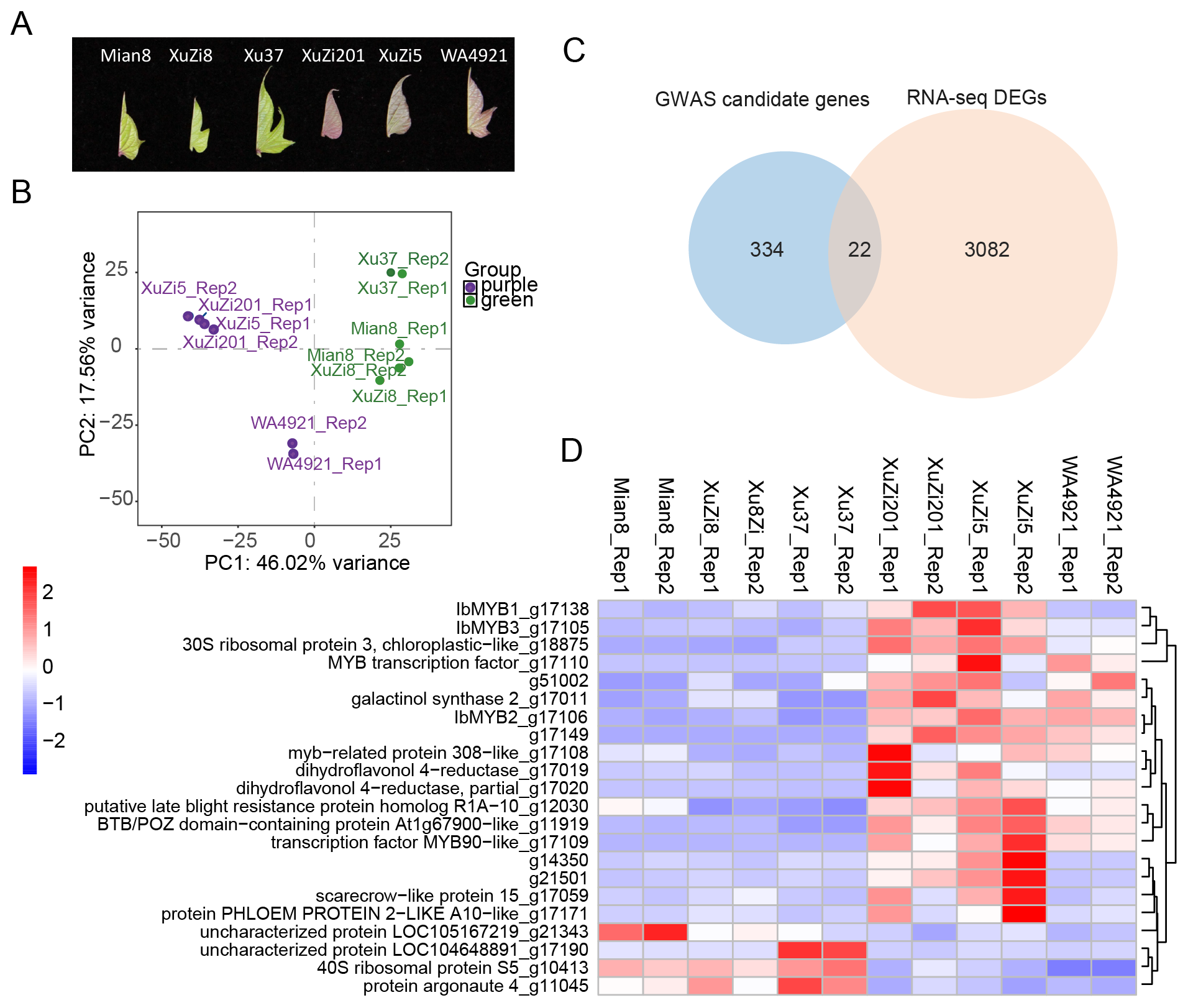


**Figure S11. RNA-seq analysis of candidate genes involved in anthocyanin accumulation in terminal buds.**
**(A)** Phenotypic comparison of green and purple terminal buds. Among these accessions, Mian8, Xu37, and WA4921 are from the 260 germplasm resources provided by Zhejiang A&F University, while XuZi201 and XuZi5 are preserved in our laboratory. Meanwhile, XuZi8 is part of the 260 accessions but also preserved in our laboratory. **(B)** PCA of RNA-seq data, showing distinct clustering of green and purple terminal buds based on transcriptional profiles. Each sample has two biological replicates. **(C)** Venn diagram showing the overlap between candidate genes identified from leaf coloration-related GWAS and DEGs from RNA-seq analysis. **(D)** Heatmap of expression profiles for 22 overlapping candidate genes across different accessions.


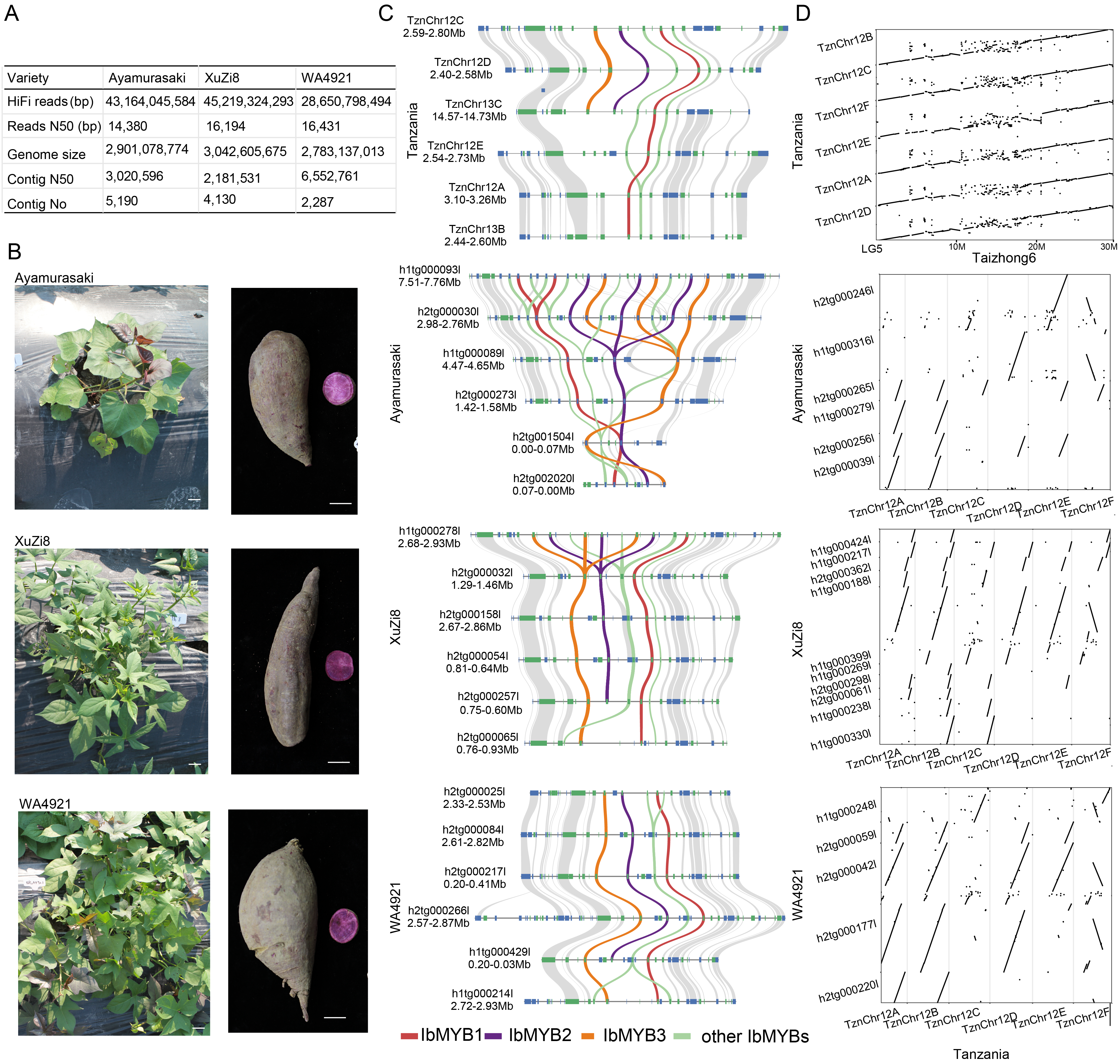


**Figure S12. Comparative genomics of MYB clusters and candidate regions on Chromosome 5 in purple-fleshed sweet potato.**

**(A)** Genome assembly statistics for three purple-fleshed cultivars: Ayamurasaki, XuZi8, and WA4921. **(B)** Representative seedlings and storage roots of the three cultivars. **(C)** Microsynteny of MYB genes across subgenomes of Tanzania, Ayamurasaki, XuZi8, and WA4921 (top to bottom). **(D)** Chromosome-level collinearity between Taizhong6 chromosome 5 and the Tanzania reference genome, together with comparative alignments of the corresponding 19–30 Mb candidate interval from Ayamurasaki, XuZi8, and WA4921 against Tanzania subgenomes (TznChr12A–F), demonstrating structural conservation and assessing the presence of large-scale rearrangements within the candidate region.


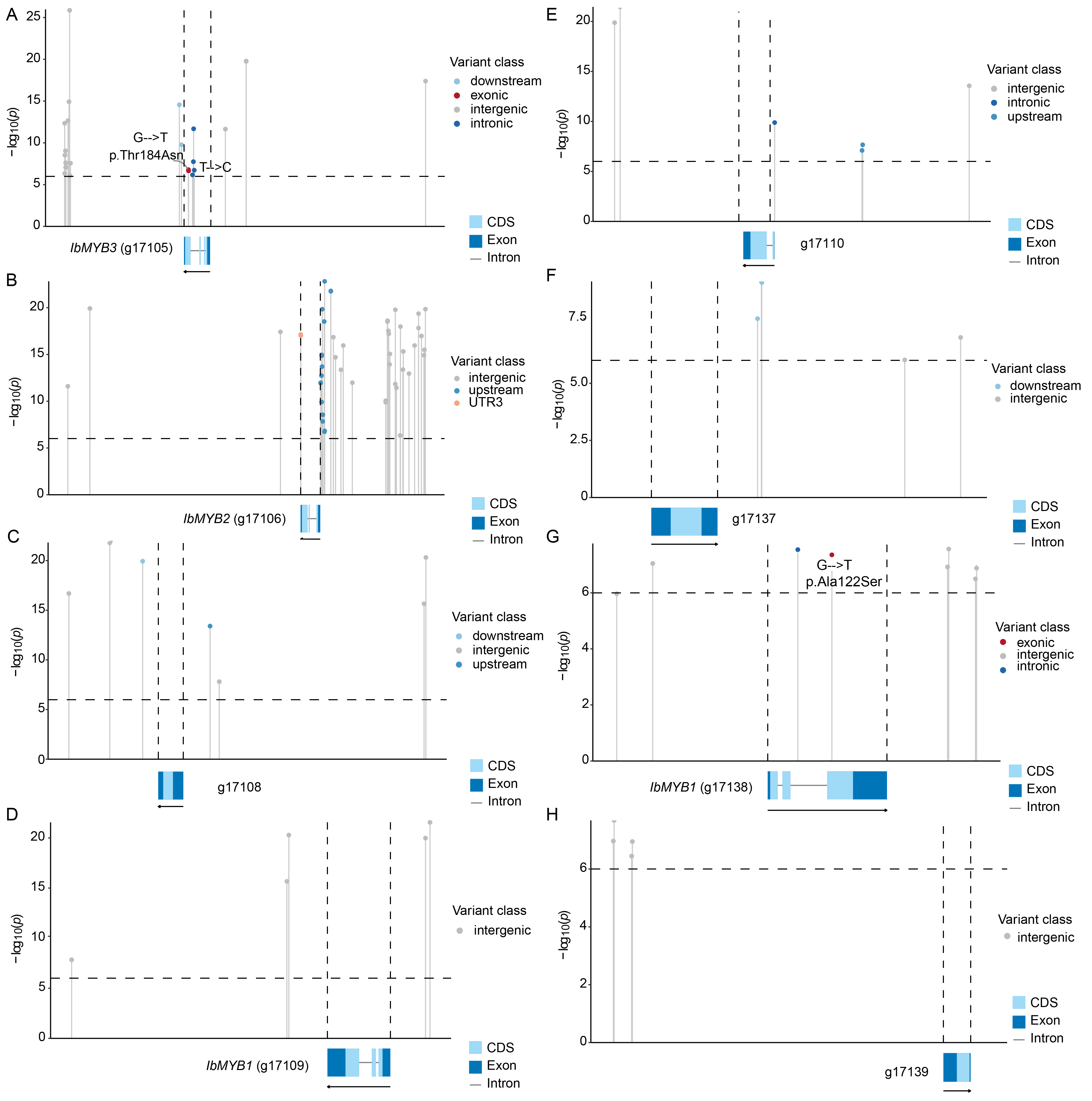

**Figure S13. Significant SNPs in candidate MYB genes.**
**(A–H)** Significant SNPs in eight candidate genes: *g17105* **(A)**, *g17106* **(B)**, *g17108* **(C)**, *g17109* **(D)**, *g17110* **(E)**, *g17137* **(F)**, *g17138* **(G)**, and *g17139* **(H)**. Only SNPs exceeding the genome-wide significance threshold (1e-06) and annotated by ANNOVAR as located within or near the gene are shown. Genomic context is indicated as follows: exonic (coding sequence, may alter protein), intronic (within introns, may affect splicing), upstream/downstream (within 1 kb of gene boundaries, may affect regulation), UTRs (5′ or 3′ untranslated regions, may influence mRNA stability or translation), and intergenic (between genes, potential regulatory effects). Vertical dashed lines mark the start and end positions of each gene.


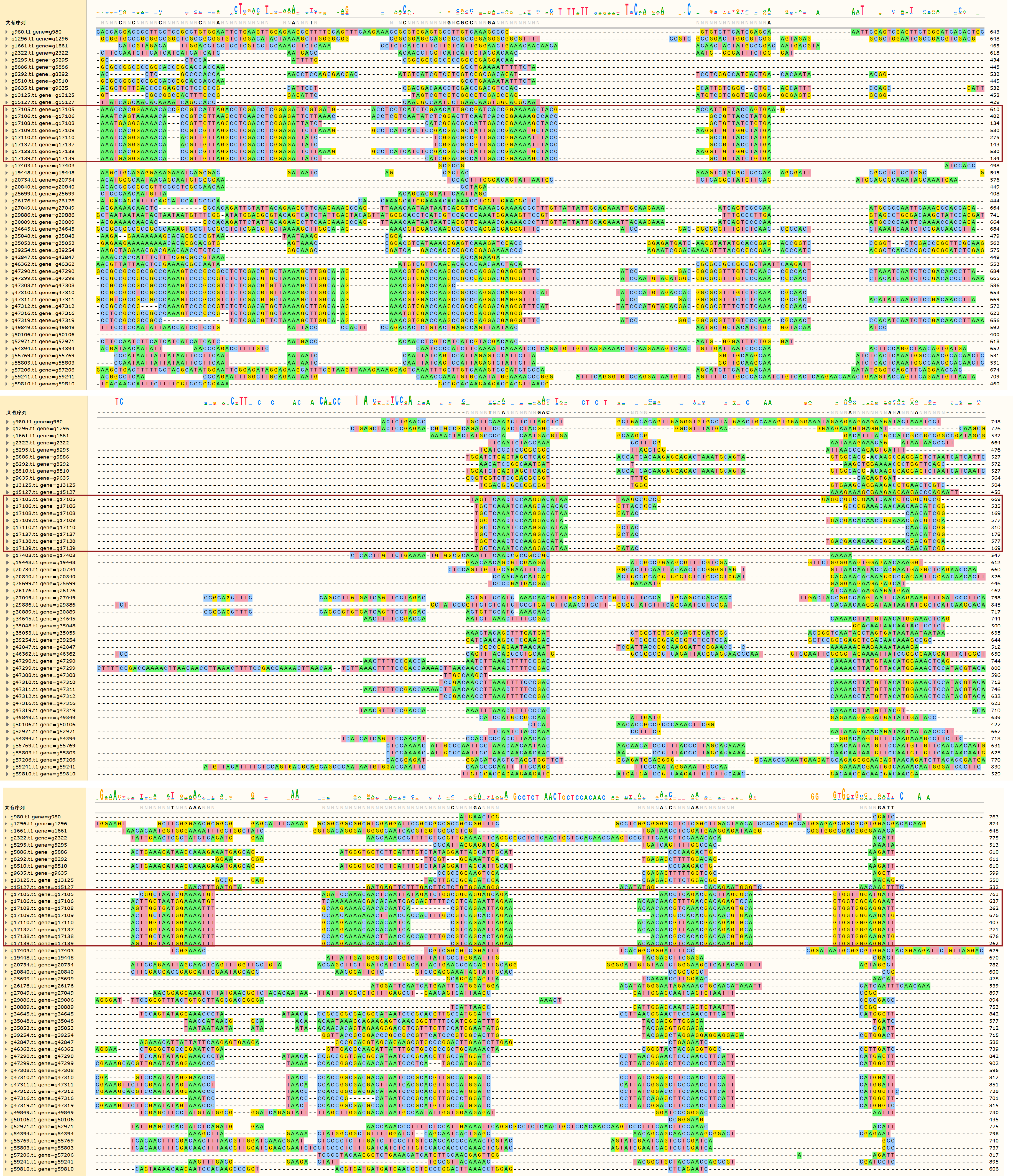


**Figure S14. MYB Homolog Alignment and Conserved VIGS Targets.**

Forty-three MYB homologs in sweet potato, identified using eight target MYB genes as queries (E-value ≤ 1e-40), were aligned with MAFFT and visualized using SnapGene. The red boxes indicate the VIGS-targeted region, which is conserved only within the two MYB clusters.

**
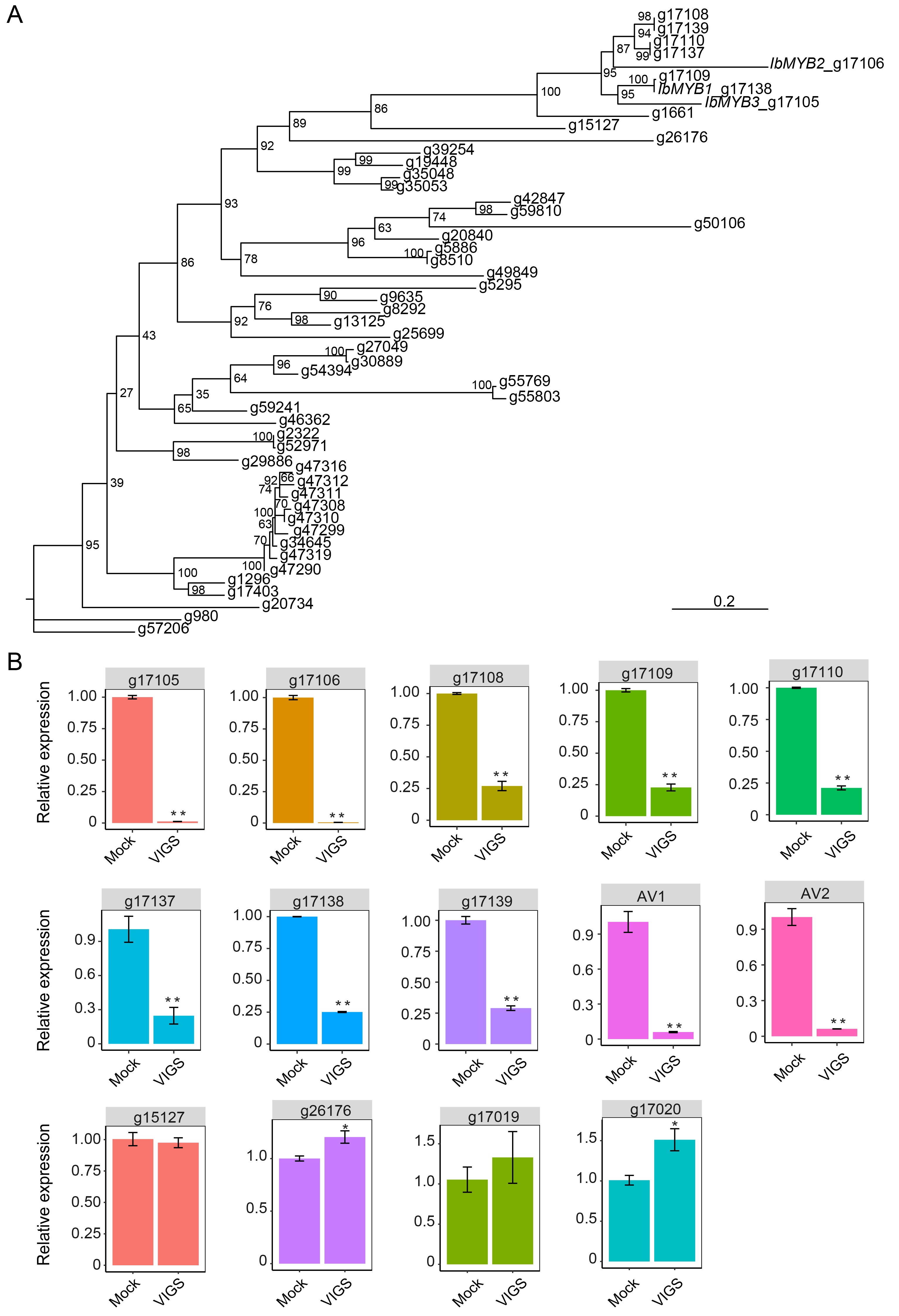
**

**Figure S15. Phylogenetic relationships and VIGS validation of MYB genes.**

(A) Phylogenetic tree constructed based on nucleotide sequence alignments of 51 MYB homologs in sweet potato. (B) Relative expression levels of MYB genes in Long14 lines subjected to VIGS knockdown or mock infection, quantified by qRT-PCR. AV1 and AV2, encoding viral coat protein, were included as controls to assess infection efficiency.


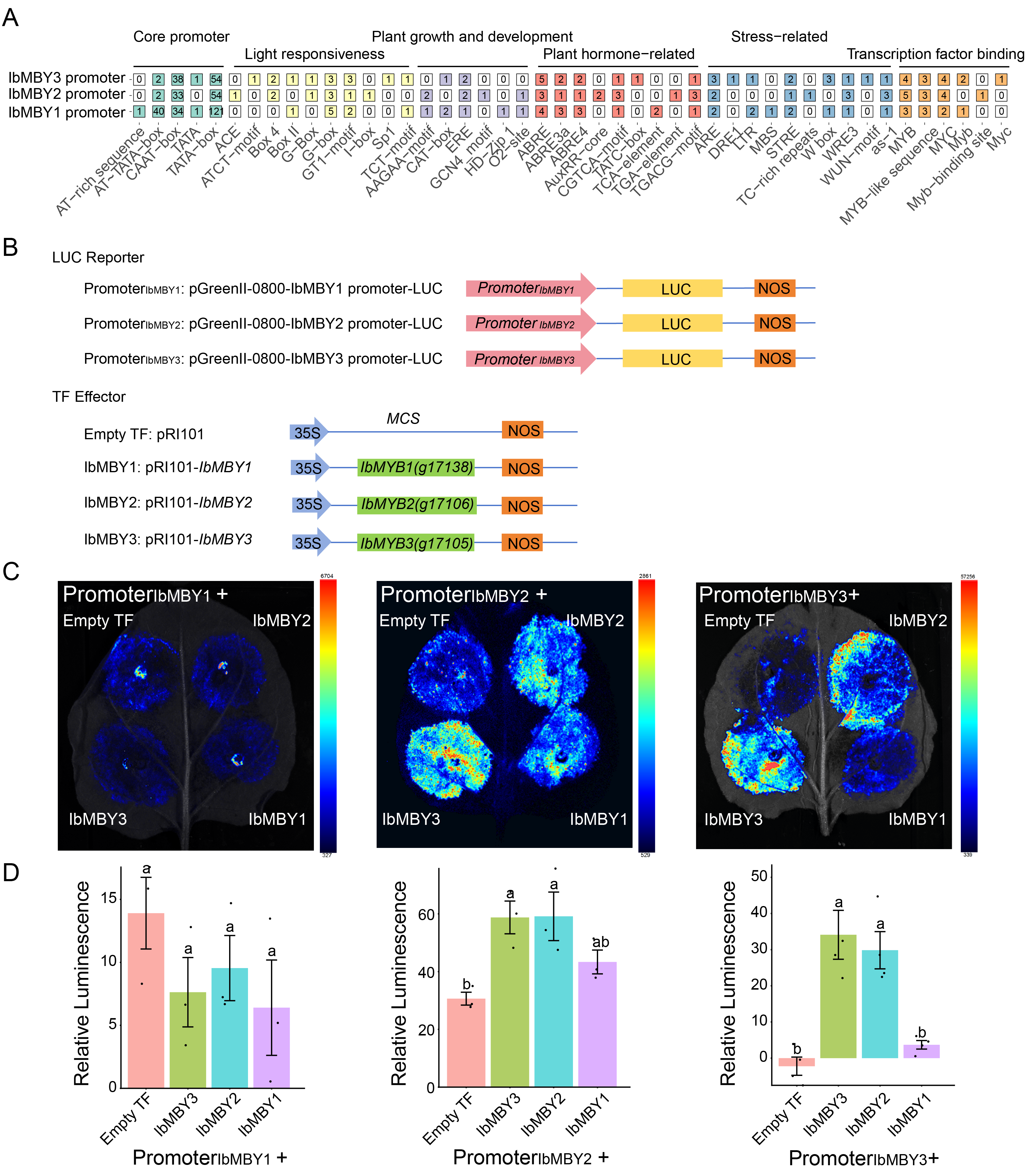


**Figure S16. Analysis of *IbMYB* promoters and transcriptional activation assays.**
**(A)** *Cis*-element analysis of IbMYB promoters. The number of *cis*-elements identified in the 2-kb upstream promoter regions of IbMYB genes using PlantCARE. **(B)** Schematic diagrams of reporter and effector constructs. Luciferase (Luc) reporter driven by *IbMYB* promoters and corresponding transcription factor (TF) effector constructs. **(C)** Transcriptional activation of *IbMYB* promoters by IbMYB proteins. Representative bioluminescence images in tobacco leaves showing activation of *IbMYB* promoters by IbMYBs.


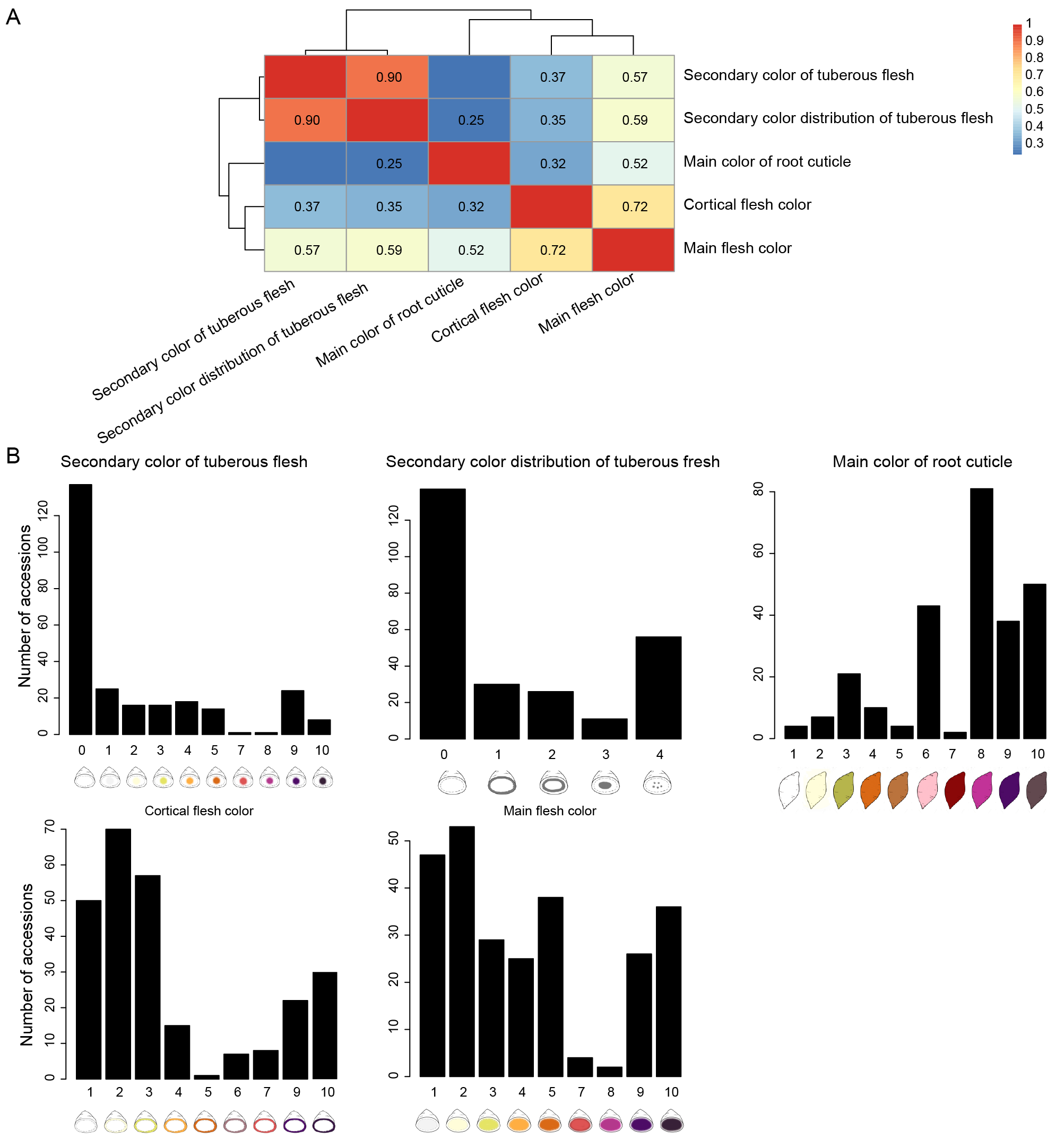


**Figure S17. Phenotypic analysis of tuberous root color traits in sweet potato.**
**(A)** Spearman correlation matrix of the five tuberous root color traits, illustrating the relationships between each trait. Correlation coefficients were calculated using Spearman’s rank correlation via the rcorr function in the R package Hmisc. Only statistically significant correlations meeting the criteria of |r| ≥ 0.25 and p ≤ 0.05 are numerically labeled, while non-significant correlations are omitted. **(B)** Bar chart depicting the distribution of tuberous root color traits across the sweet potato population, illustrating the phenotypic variation. The x-axis represents the quantified phenotypic scores, and the y-axis indicates the number of accessions.

**
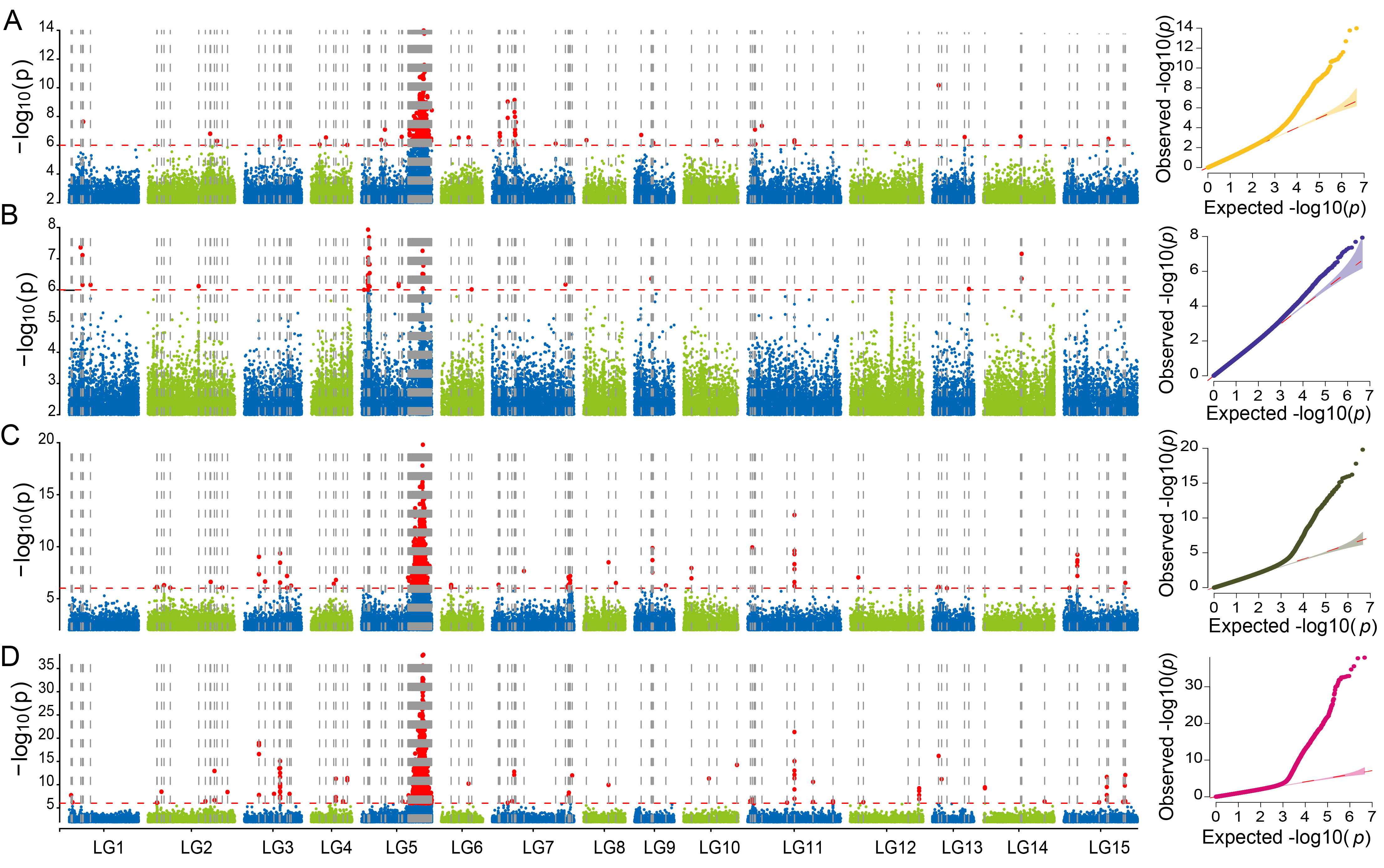
**

**Figure S18. GWAS identification of significant loci associated with tuberous root pigmentation.
(A-D)** Manhattan and Quantile-Quantile (Q-Q) plots for various traits: secondary color of tuberous flesh **(A)**, main color of root cuticle **(B)**, cortical flesh color **(C)**, and main flesh color **(D)**. Grey lines connect SNPs at identical physical positions that exceed the significance threshold in at least one trait, providing a visual reference for comparing SNP locations across traits; these lines do not indicate statistical colocalization. Trait-specific significant SNPs (p ≤ 1e-06) are highlighted as red points. Red dashed lines represent the genome-wide significance threshold in the Manhattan plots.


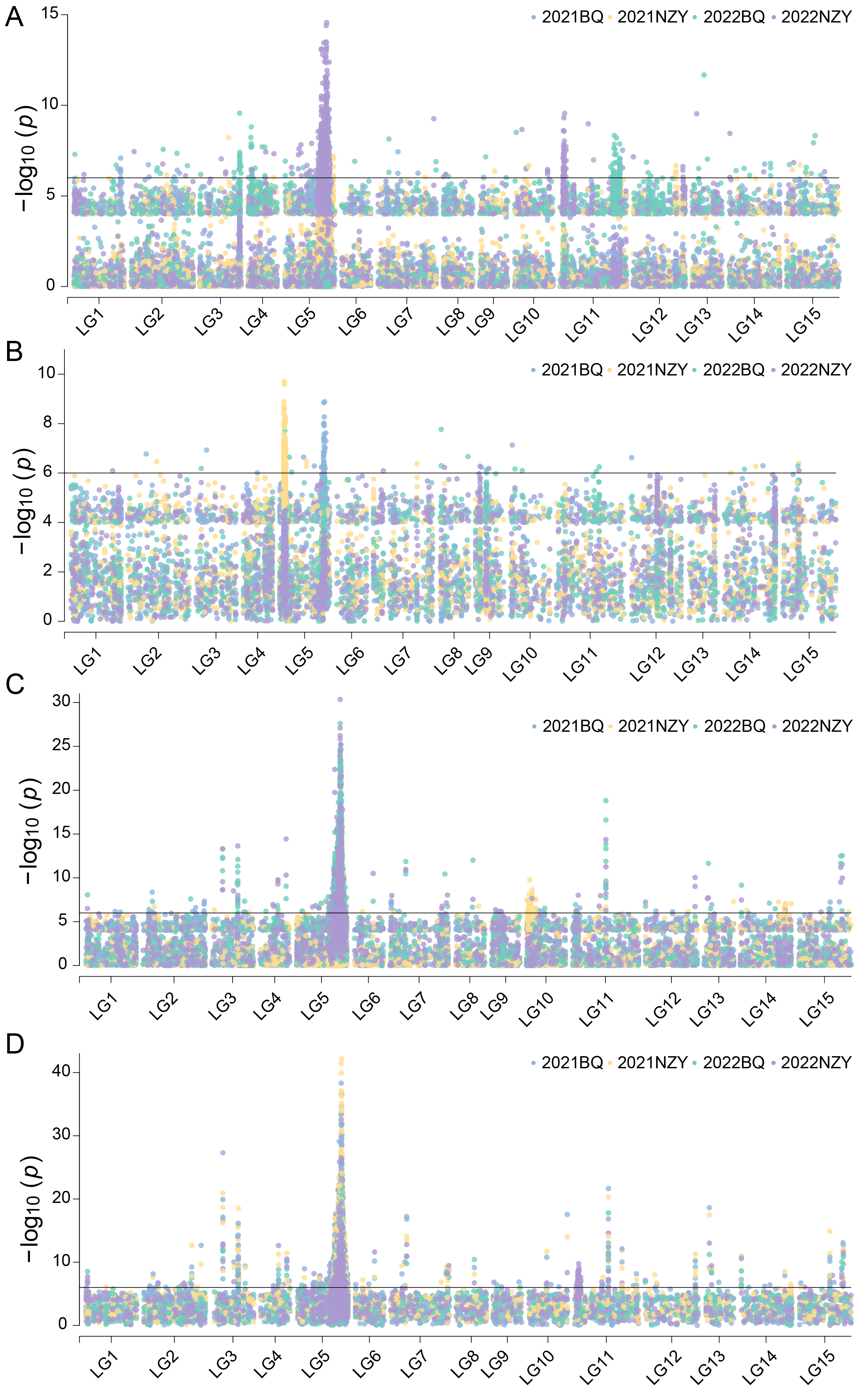


**Figure S19. GWAS identification of significant loci associated with tuberous root pigmentation.**

**(A–D)** Manhattan plots for various traits: secondary color of tuberous flesh **(A)**, main color of root cuticle **(B)**, cortical flesh color **(C)**, and main flesh color **(D)**. Phenotypic values measured in each of the four environments (2021BQ, 2021NZY, 2022BQ, 2022NZY) were used for GWAS. The black horizontal lines indicate the genome-wide significance threshold (1e-06).

**
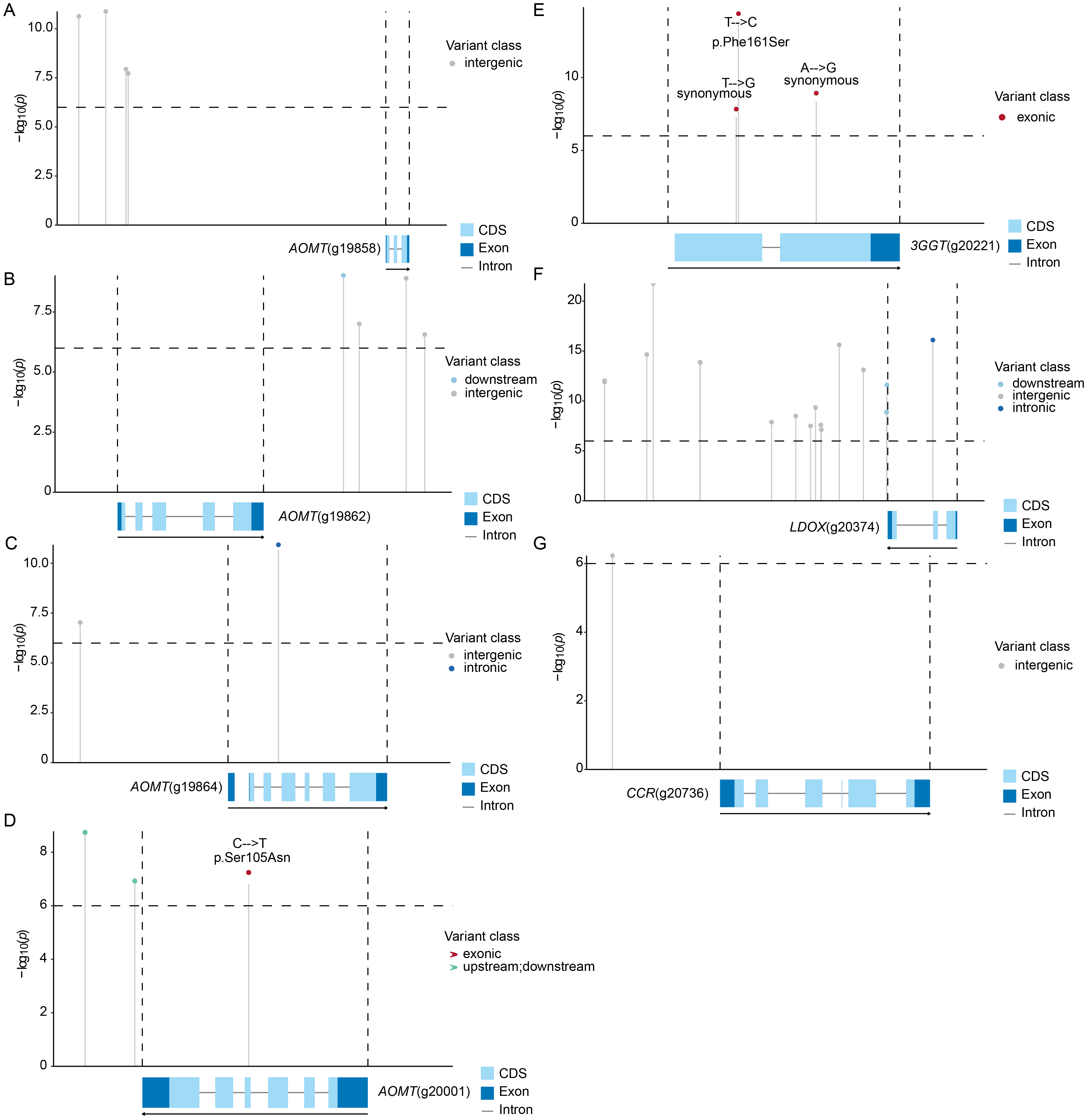
**

**Figure S20. Significant SNPs in anthocyanin structural genes.**
**(A–G)** Significant SNPs in seven candidate genes: *g19858* **(A)**, *g19862* **(B)**, *g19864* **(C)**, *g20001* **(D)**, *g20221* **(E)**, *g20374* **(F)**, and *g20376* **(G)**. Only SNPs exceeding the genome-wide significance threshold (1e-06) and annotated using ANNOVAR as located within or near the gene are shown. Genomic context is indicated as follows: exonic (coding sequence, may alter protein), intronic (within introns, may affect splicing), upstream/downstream (within 1 kb of gene boundaries, may affect regulation), UTRs (5′ or 3′ untranslated regions, may influence mRNA stability or translation), and intergenic (between genes, potential regulatory effects). Vertical dashed lines mark the start and end positions of each gene.


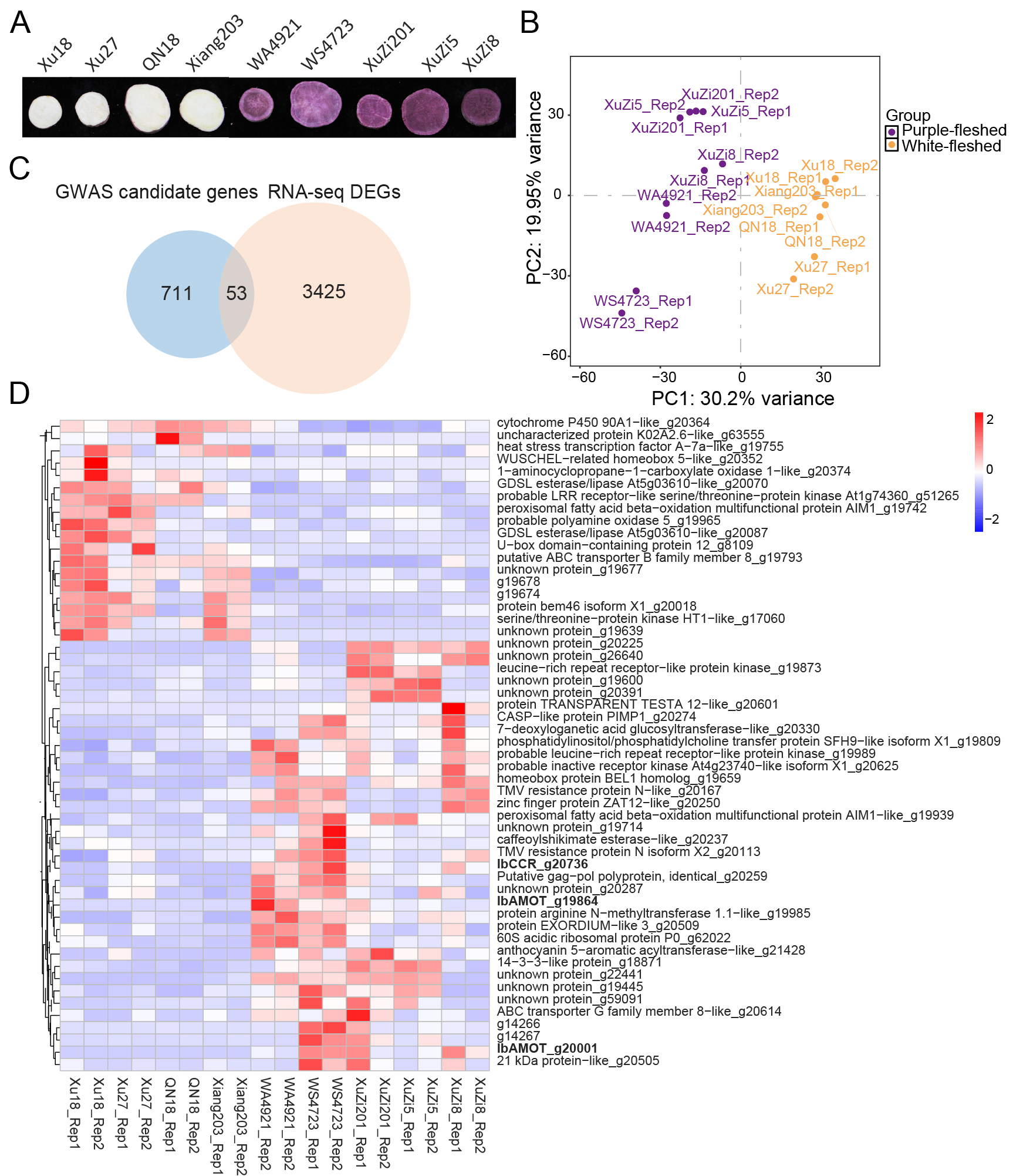


**Figure S21. RNA-seq analysis of candidate genes involved in anthocyanin accumulation in tuberous root flesh.**
**(A)** Phenotypic comparison of white-fleshed and purple-fleshed accessions used in RNA-seq analysis. QN18, Xiang203, WA4921, and WS4723 are part of the 260 accessions provided by Zhejiang A&F University, while Xu27, XuZi201, and XuZi5 are preserved in our laboratory. Meanwhile, XuZi8 and Xu18 belong to the 260 accessions but are also maintained in our laboratory. **(B)** PCA of RNA-seq data, showing a clear separation between white- and purple-fleshed accessions based on transcriptional profiles. Each accession was analyzed with two biological replicates. **(C)** Venn diagram illustrating the overlap between overall tuberous root pigmentation GWAS candidate genes and DEGs from RNA-seq analysis. **(D)** Heatmap depicting the expression profiles of 53 overlapping candidate genes across different accessions.


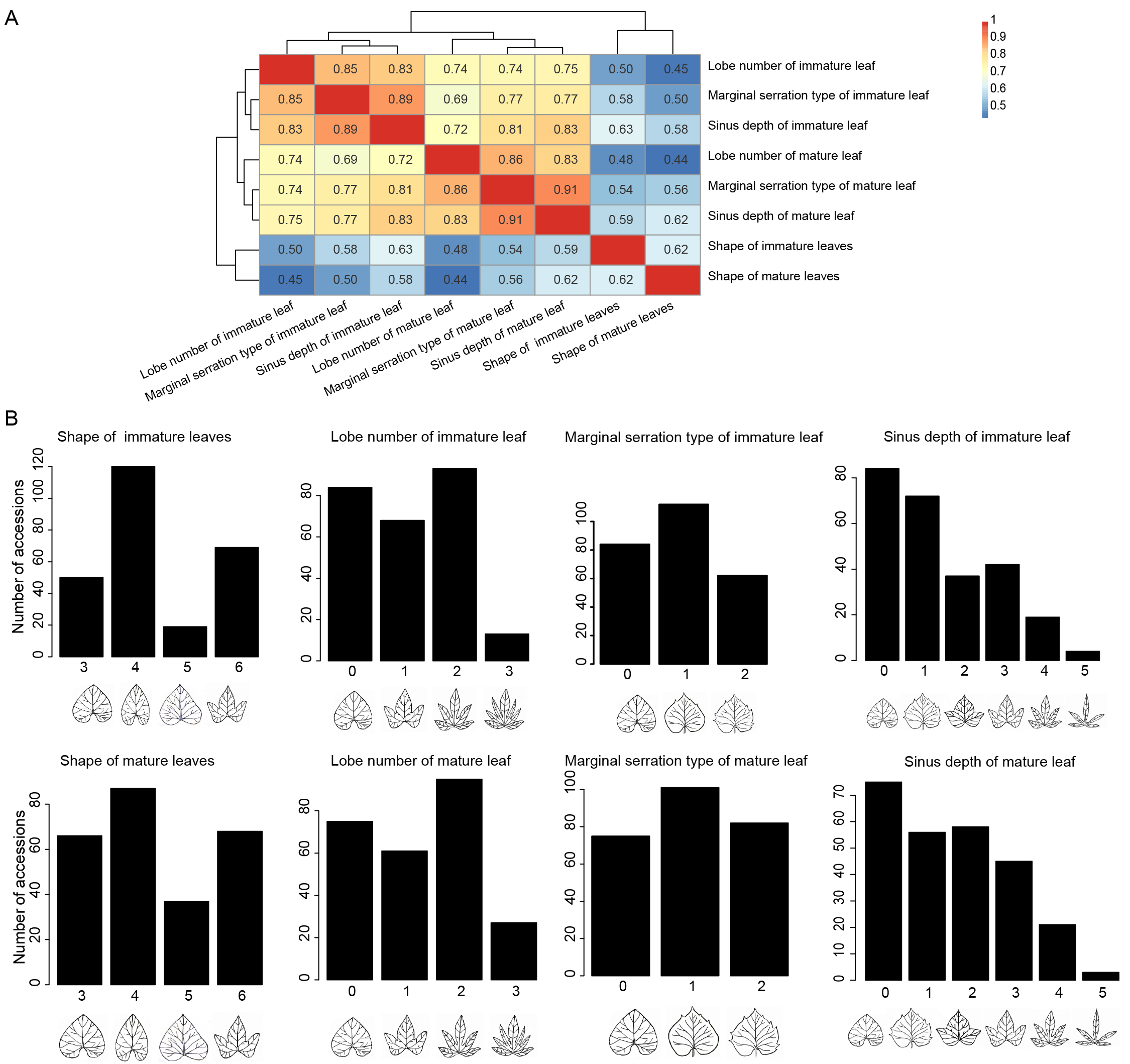


**Figure S22. Phenotypic analysis of leaf shape traits in sweet potato.**
**(A)** Spearman correlation matrix of eight leaf shape traits, illustrating their interrelationships. Correlation coefficients were calculated using Spearman’s rank correlation via the rcorr function in the R package Hmisc. Only statistically significant correlations meeting the criteria of |r| ≥ 0.25 and *p* ≤ 0.05 are numerically labeled, while non-significant correlations are omitted. **(B)** Bar chart showing the distribution of leaf shape traits across the sweet potato population, reflecting phenotypic variation. The x-axis represents the quantified phenotypic scores, and the y-axis indicates the number of accessions.


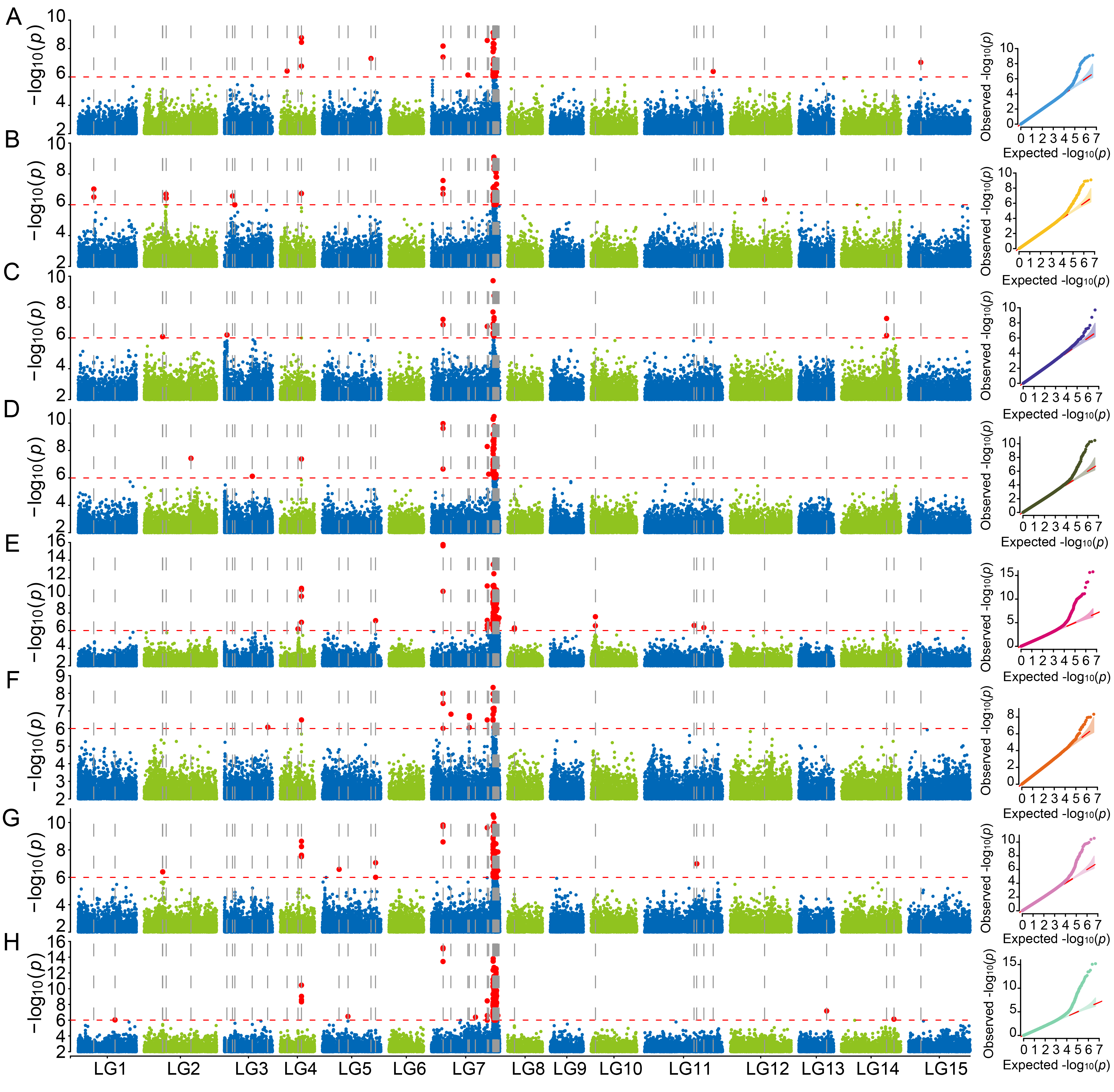


**Figure S23. GWAS identification of significant loci associated with leaf shape.**
**(A–H)** Manhattan and Quantile-Quantile (Q-Q) plots for various traits: shape of immature leaf **(A)**, shape of mature leaf **(B)**, lobe number of immature leaf **(C)**, marginal serration type of immature leaf **(D)**, sinus depth of immature leaf **(E)**, lobe number of mature leaf **(F)**, marginal serration type of mature leaf **(G)**, and sinus depth of mature leaf **(H)**. Grey lines connect SNPs at identical physical positions that exceed the significance threshold in at least one trait, providing a visual reference for comparing SNP locations across traits; these lines do not indicate statistical colocalization. Trait-specific significant SNPs (*p* ≤ 1e06) are highlighted as red points. Red dashed lines represent the genome-wide significance threshold in the Manhattan plots.


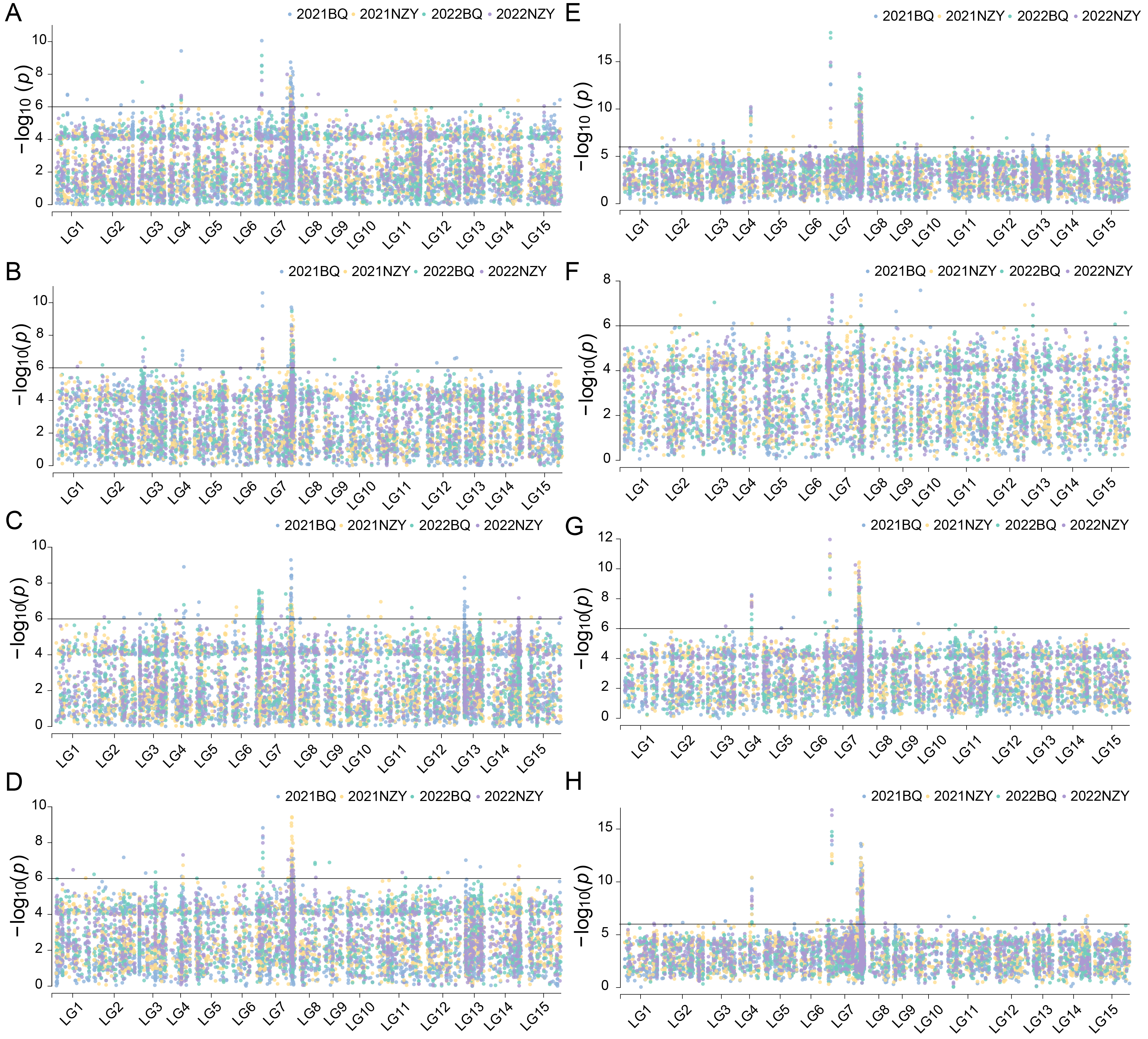


**Figure S24. GWAS identification of significant loci associated with leaf shape across two years and two locations.**
**(A–H)** Manhattan plots for eight leaf shape traits: shape of immature leaf **(A)**, shape of mature leaf **(B)**, lobe number of immature leaf **(C)**, marginal serration type of immature leaf **(D)**, sinus depth of immature leaf **(E)**, lobe number of mature leaf **(F)**, marginal serration type of mature leaf **(G)**, and sinus depth of mature leaf **(H)**. Phenotypic values measured in each of the four environments (2021BQ, 2021NZY, 2022BQ, 2022NZY) were used for GWAS. The black horizontal lines indicate the genome-wide significance threshold (1e-06).


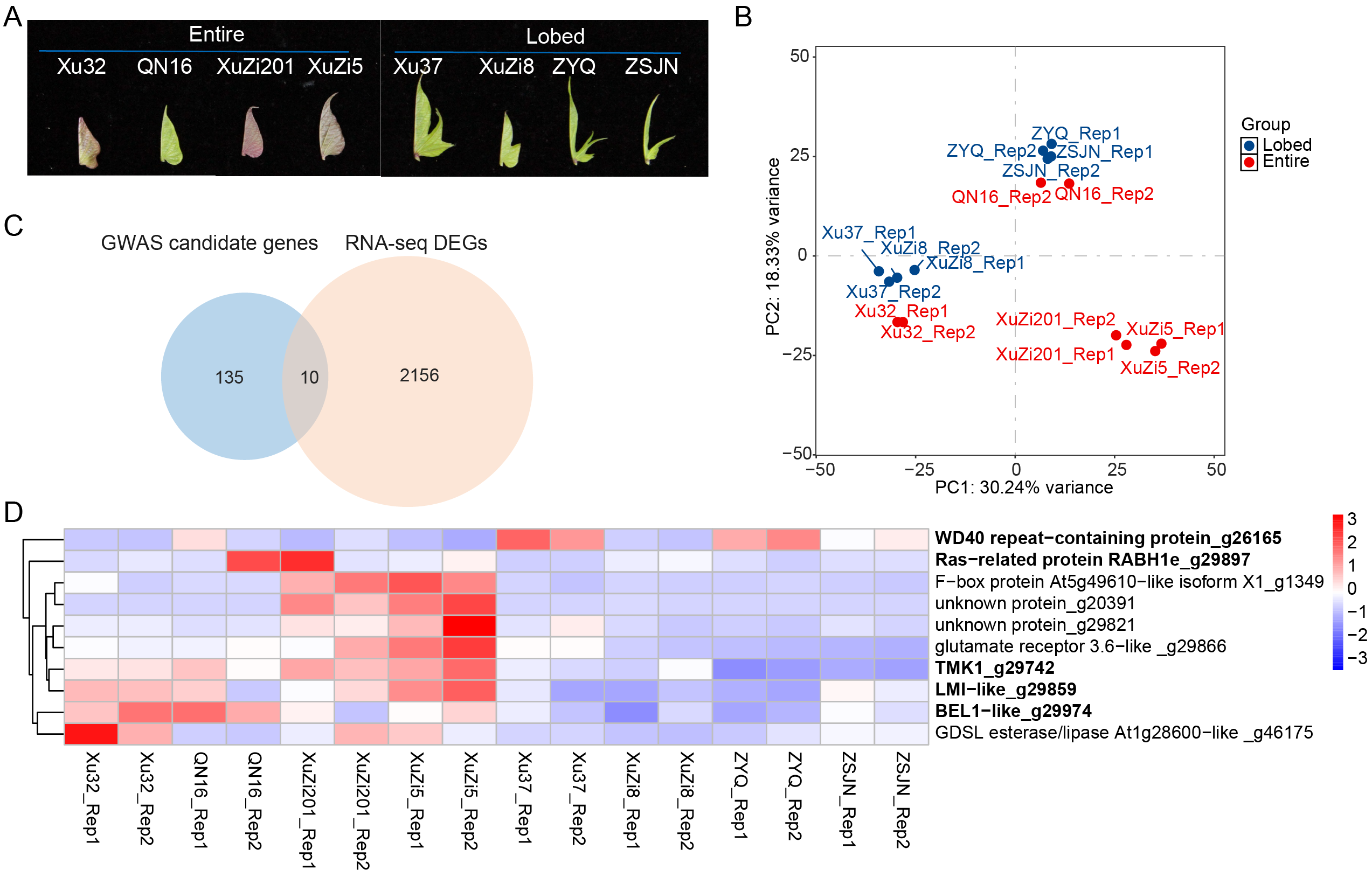


**Figure S25. RNA-seq analysis of candidate genes involved in leaf shape variation.**
**(A)** Phenotypic comparison between entire-leaf and lobed-leaf accessions. Young, unexpanded leaves were collected from each accession at the same developmental stage under uniform growth conditions. Xu32, Xu37, and QN16 were part of the 260-accession panel provided by Zhejiang A&F University, whereas ZYQ, ZSJN, XuZi201, and XuZi5 are preserved in our laboratory. XuZi8 belongs to the 260-accession panel and is also maintained in our laboratory. **(B)** PCA of leaf shape RNA-seq data. Each accession was analyzed with two biological replicates. **(C)** Venn diagram illustrating the overlap between candidate genes identified from leaf shape-related GWAS and DEGs from RNA-seq analysis. **(D)** Heatmap of expression profiles for the 10 overlapping candidate genes across diverse accessions. Each accession was analyzed with two biological replicates.


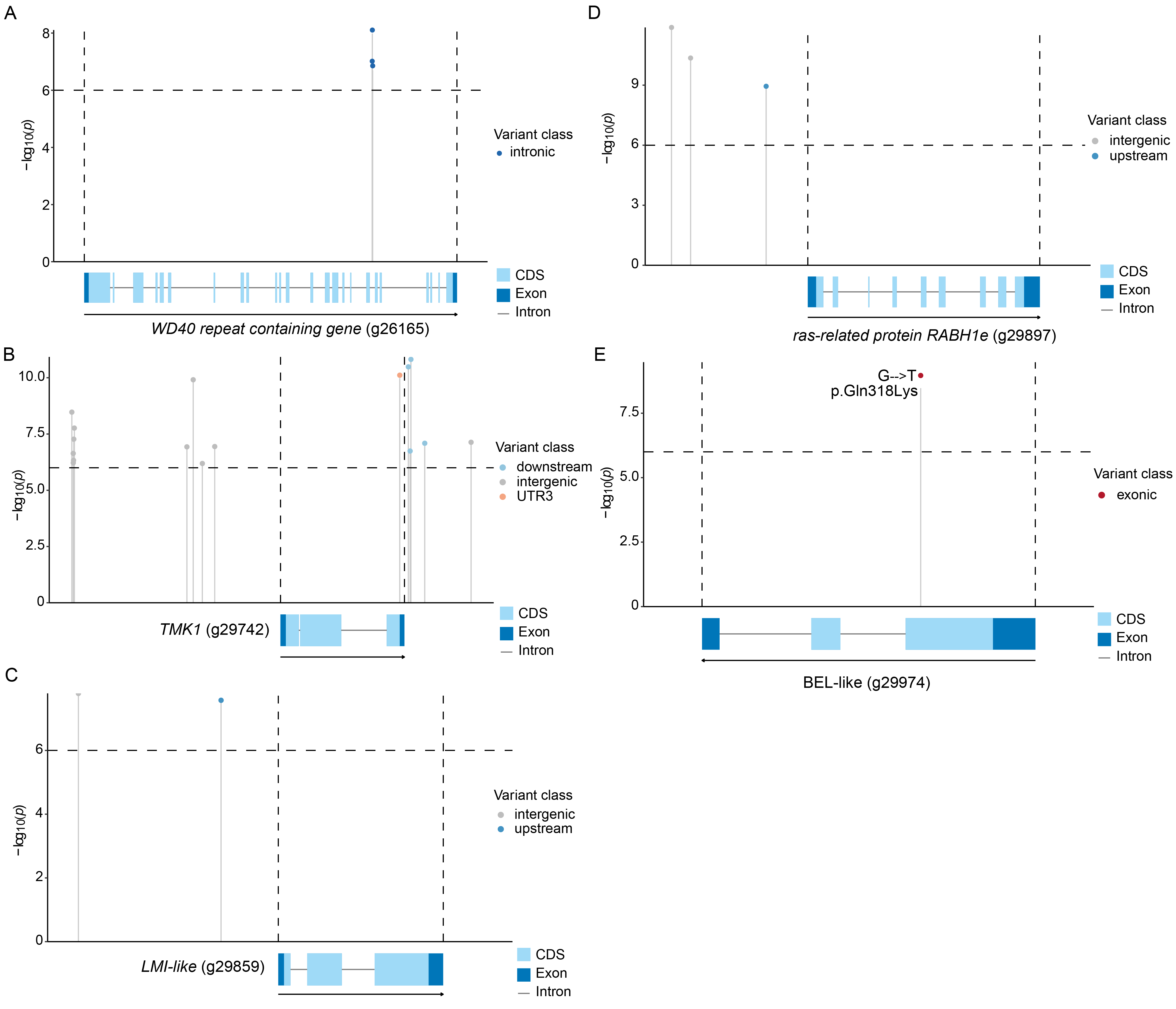


**Figure S26. Significant SNPs in candidate leaf-shape genes.**
**(A–E)** Significant SNPs in five candidate genes: *g26165* **(A)**, *g29742* **(B)**, *g29859* **(C)**, *g29897* **(D)**, and *g29974* **(E)**. Only SNPs exceeding the genome-wide significance threshold (1e-06) and annotated using ANNOVAR as located within or near the gene are shown. Genomic context is indicated as follows: exonic (coding sequence, may alter protein), intronic (within introns, may affect splicing), upstream/downstream (within 1 kb of gene boundaries, may affect regulation), UTRs (5′ or 3′ untranslated regions, may influence mRNA stability or translation), and intergenic (between genes, potential regulatory effects). Vertical dashed lines mark the start and end positions of each gene.


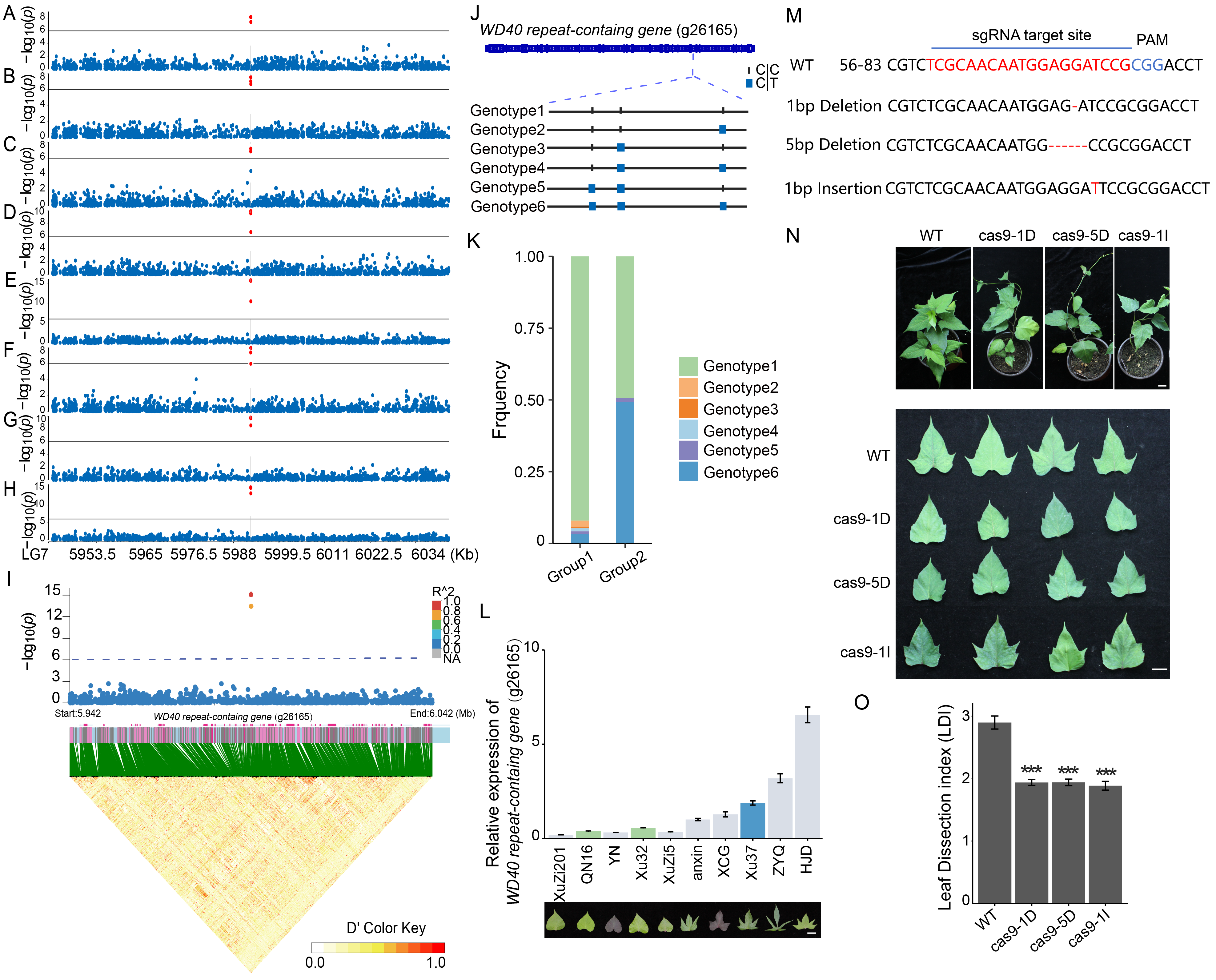


**Figure S27. Genotypes and expression analysis of the candidate *WD40 repeat-containing* gene (*g26165*) for leaf shape.
(A–H)** Manhattan plots for eight traits: shape of immature leaf **(A)**, shape of mature leaf **(B)**, lobe number of immature leaf **(C)**, marginal serration type of immature leaf **(D)**, sinus depth of immature leaf **(E)**, lobe number of mature leaf **(F)**, marginal serration type of mature leaf **(G)**, and sinus depth of mature leaf **(H)** within the WD40 repeat-containing gene region. The black horizontal lines represent the statistical significance threshold (1e-06). **(I)** LD analysis showing significant SNPs within the WD40 repeat-containing gene region. **(J)** Schematic representation of the *WD40 repeat-containing gene* (*g26165*) structure, illustrating three distinct SNP variations that give rise to six different genotypes. All the blue squares represent CT alleles, while the black line indicates the reference CC alleles. **(K)** Allele frequency distribution between the two groups, as shown in Figure 6C. **(L)** qRT-PCR analysis of *WD40 repeat-containing gene* (*g26165*) expression across accessions with varying leaf shapes. QN16, Xu32, and Xu37 are part of the 260 samples provided by Zhejiang A&F University, while YN, anxin, XCG, HJD, ZYQ, XuZi5, and XuZi201 are preserved in our lab. The colors represent the different genotypes described above, with gray indicating unknown genotype. **(M)** Mutation types and genotyping results of three independent CRISPR/Cas9-edited *g26165* knockout lines in the lobed-leaf sweet potato cultivar Xushu 22. Small insertions and deletions were detected at the target site, including 1-bp and 5-bp deletions and a 1-bp insertion. **(N)** Representative leaf phenotypes of wild-type (WT) and g26165 knockout lines. Compared with WT plants, all knockout lines exhibit reduced leaf lobe depth and shallower leaf margins. Scale bar = 2 cm. **(O)** Quantitative analysis of leaf dissection index (LDI) in WT and g26165 knockout lines. LDI values are significantly reduced in knockout lines relative to WT, indicating attenuated leaf lobing. Data represent mean ± SD (n = 12). Statistical significance was determined by Student’s t-test (**p* < 0.05; ***p* < 0.01; ****p* < 0.001).


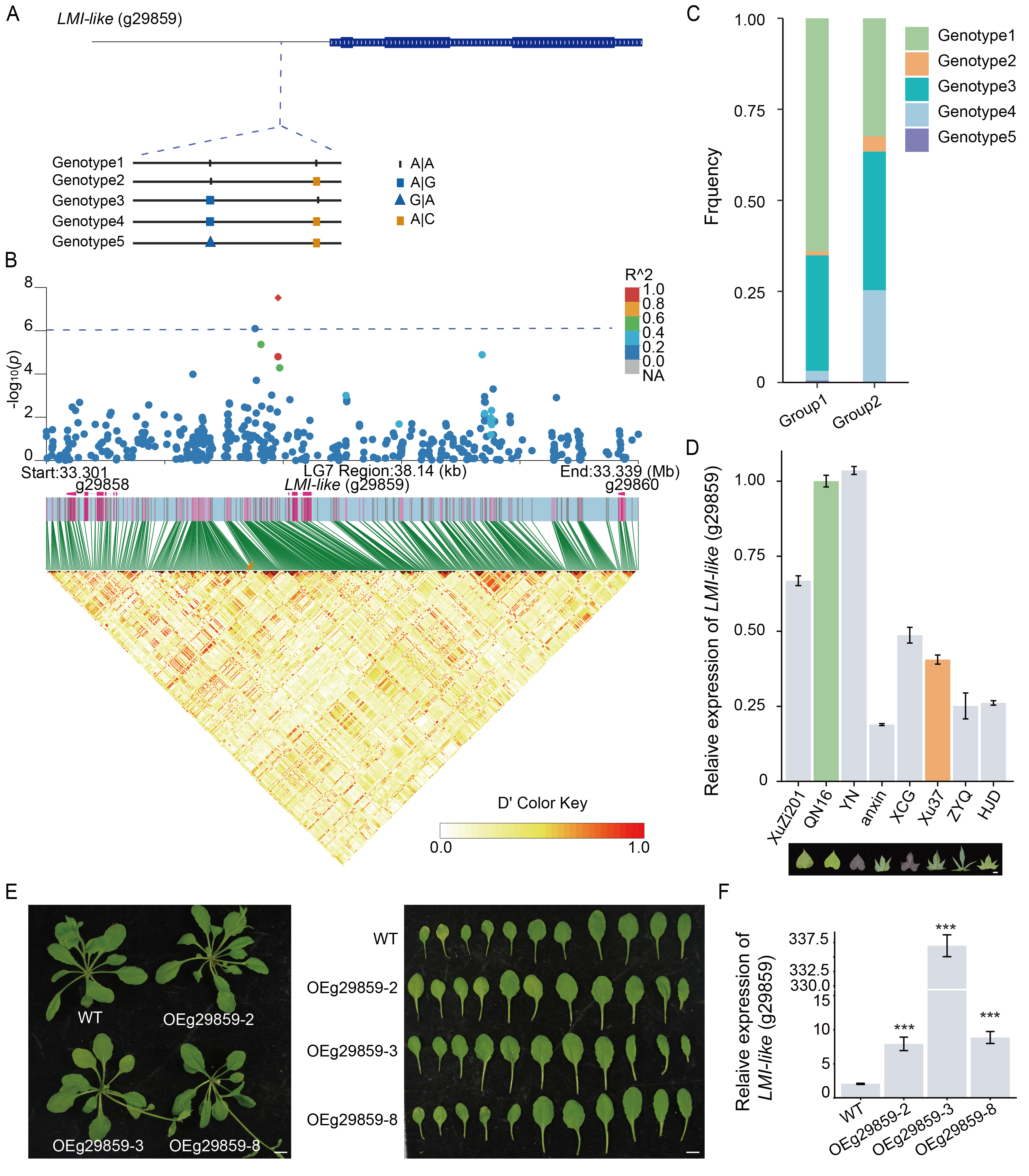


**Figure S28. Genotypes and expression analysis of the candidate *LMI-like* gene (*g29859*) for leaf notch depth.
(A)** Schematic representation of the *LMI-like* gene (*g29859*) structure, showing two distinct SNP variations in the upstream region that give rise to five major genotypes. **(B)** LD analysis of the *LMI-like* gene (*g29859*) region. The black horizontal lines represent the statistical significance threshold (1e-06) in GWAS. **(C)** Allele frequency distribution between the two groups, as shown in Figure 6C. **(D)** qRT-PCR analysis of *LMI-like gene* (*g29859*) expression across accessions with varying leaf shapes. QN16 and Xu37 are part of the 260-accession panel provided by Zhejiang A&F University, while YN, Anxin, XCG, HJD, ZYQ and XuZi201 are preserved in our laboratory. The colors represent the different genotypes described above, with gray indicating unknown genotype. **(E)** Representative leaf phenotypes of wild-type (WT) and *g29859* overexpression lines in *Arabidopsis thaliana*. Transgenic plants display enhanced leaf lobing compared with WT. **(F)** qRT-PCR analysis of g29859 expression in WT and three independent overexpression lines. Data are presented as mean ± SE (n = 3 biological replicates). Statistical significance was determined by Student’s t-test (****p* < 0.001). Scale bar = 1 cm.


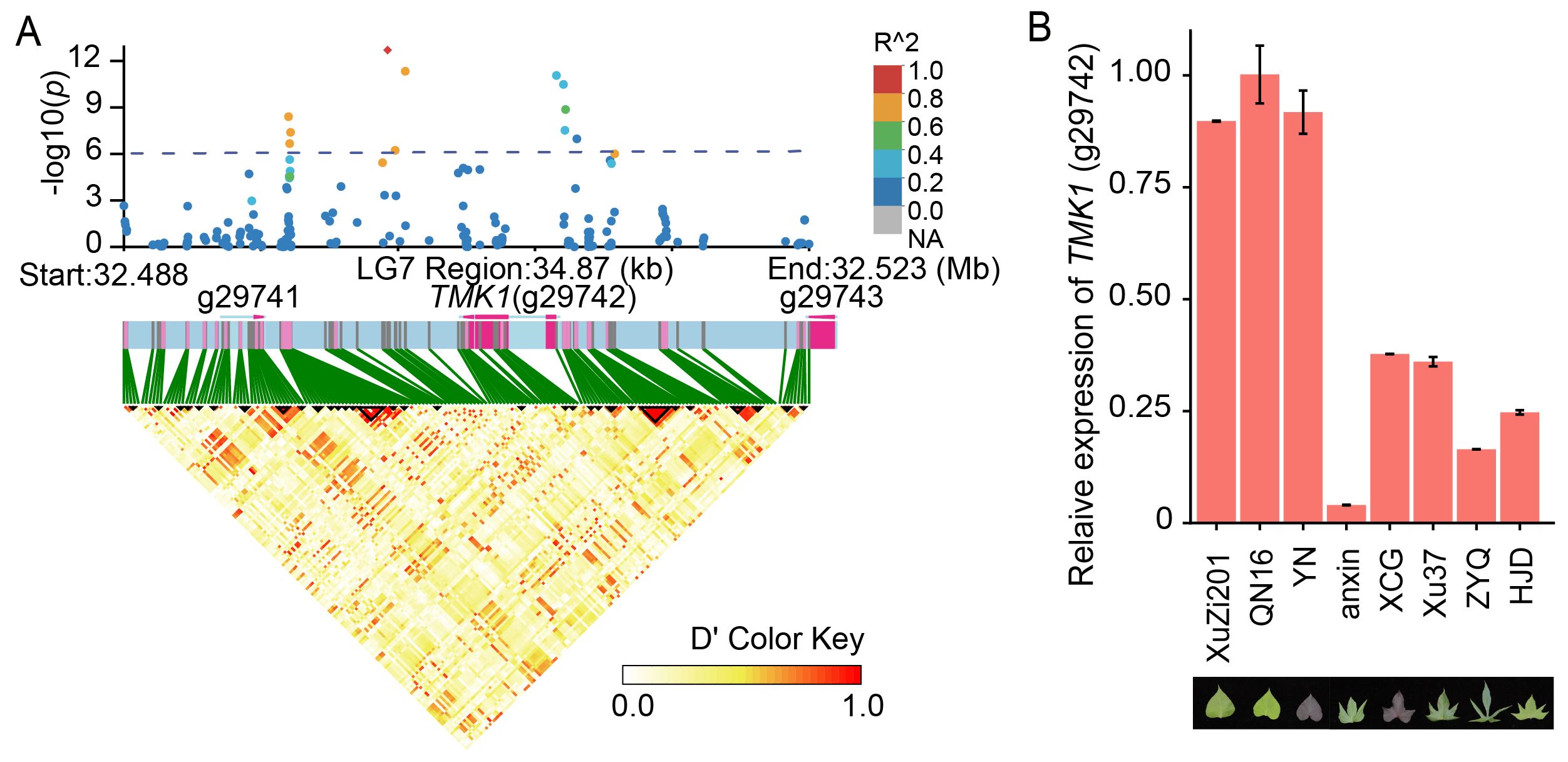


**Figure S29. LD block and expression analysis of the candidate *TMK1* gene (*g29742*) for leaf notch depth.**

**(A)** LD analysis of the *TMK1* gene (g29742) region. The black horizontal lines represent the statistical significance threshold (1e-06) in GWAS. **(B)** qRT-PCR analysis of *TMK1* (*g29742*) expression across accessions with varying leaf shapes. QN16 and Xu37 are part of the 260-accession panel provided by Zhejiang A&F University, while YN, Anxin, XCG, HJD, ZYQ, and XuZi201 are preserved in our lab.

**Supplementary Materials and Methods**

**Comparative genome evolution**

Genome assemblies and corresponding gene annotation files for five species, *Ipomoea trifida* (Y22) (Li et al., 2019), *Ipomoea triloba* (NSP323) (Wu et al., 2018), *Ipomoea nil* (Hoshino et al., 2016), *Ipomoea purpurea* (Gupta et al., 2021), and *Cuscuta europaea* (Neumann et al. 2023), were downloaded from public databases. The longest transcript was extracted using custom scripts, and the corresponding protein sequences were generated using gffread.

Orthologous gene families were identified using OrthoFinder (Emms and Kelly 2015). Single-copy orthologous gene families were aligned using MAFFT v7.0 (Katoh and Standley 2013), and poorly aligned regions were removed with trimAl v1.4.rev22 (Capella-Gutiérrez et al. 2009). A maximum-likelihood phylogenetic tree was constructed using RAxML v8.2 with the GTRGAMMA substitution model and 100 bootstrap replicates using *Cuscuta europaea* as the outgroup (Stamatakis 2014).

Divergence times were estimated under a relaxed molecular clock model using MCMCTree implemented in PAML v4.9e (Yang 2007). The divergence time between *Cuscuta europaea* and *Ipomoea* species (34.7–56.5 MYA) was used as the calibration point.

Based on the inferred phylogeny and gene family clustering results, gene family expansion and contraction were analyzed using CAFE v4.2 (De Bie et al. 2006). Synteny relationships and local gene organization were further examined using JCVI (Tang et al. 2008) to compare the structure of MYB gene clusters across *Ipomoea* species and *Cuscuta europaea*.

**Gene expression analysis by qRT-PCR and phenotypic observation**

For qRT-PCR analysis of sweet potato genes (Figures 3A,3B, 5G, 7F, and Figures S27L, S28D, S29B), the first and second fully expanded leaves and the flesh of 5-month-old tuberous roots were collected for RNA extraction. For gene expression analysis in transgenic *Arabidopsis thaliana* lines (Figures 4B, 7H, and Figure S28F), rosette leaves from plants grown in soil for 2–3 weeks were harvested for qRT-PCR. For phenotypic analysis of transgenic *Arabidopsis* plants (Figure 4), seedlings were grown on culture medium for 14 days and then transferred to soil. Photographs were taken at three developmental stages: 7 days on culture medium and 15 and 30 days after transfer to soil.

**References**

Chang, L., G. Mei, Y. Hu, J. Deng, and T. Zhang. 2019. "LMI1 Like and KNOX1 Genes Coordinately Regulate Plant Leaf Development in Dicotyledons." *Plant Molecular Biology* 99: 449–460.

De Bie, T., N. Cristianini, J. P. Demuth, and M. W. Hahn. 2006. "CAFE: A Computational Tool for the Study of Gene Family Evolution." *Bioinformatics* 22: 1269–1271.

Emms, D. M., and S. Kelly. 2015. "OrthoFinder: Solving Fundamental Biases in Whole Genome Comparisons Dramatically Improves Orthogroup Inference Accuracy." *Genome Biology* 16: 157.

Gupta, S., A. Harkess, A. Soble, M. Van Etten, J. Leebens-Mack, and R. S. Baucom. 2021. "Inter-chromosomal Linkage Disequilibrium and Linked Fitness Cost Loci Influence the Evolution of Nontarget Site Herbicide Resistance in an Agricultural Weed." *bioRxiv*

Hoshino, A., V. Jayakumar, E. Nitasaka, A. Toyoda, H. Noguchi, T. Itoh, T. Shin-I, Y. Minakuchi, Y. Koda, A. J. Nagano, et al. 2016. "Genome Sequence and Analysis of the Japanese Morning Glory Ipomoea nil." *Nature Communications* 7: 13295.

Li, M., S. Yang, W. Xu, Z. Pu, J. Feng, Z. Wang, C. Zhang, M. Peng, C. Du, F. Lin, et al. 2019. "The Wild Sweetpotato (*Ipomoea trifida*) Genome Provides Insights into Storage Root Development." *BMC Plant Biology* 19: 119.

Neumann, P., L. Oliveira, T. Jang, P. Novák, A. Koblížková, V. Schubert, A. Houben, and J. Macas. 2023. "Disruption of the Standard Kinetochore in Holocentric Cuscuta Species." *Proceedings of the National Academy of Sciences of the United States of America* 120: e2300877120.

Stamatakis, A. 2014. "RAxML Version 8: A Tool for Phylogenetic Analysis and Post-analysis of Large Phylogenies." *Bioinformatics* 30: 1312–1313.

Wu, S., K. H. Lau, Q. Cao, J. P. Hamilton, H. Sun, C. Zhou, L. Eserman, D. C. Gemenet, B. A. Olukolu, H. Wang, et al. 2018. "Genome Sequences of Two Diploid Wild Relatives of Cultivated Sweetpotato Reveal Targets for Genetic Improvement." *Nature Communications* 9: 4580.

Yang, Z. 2007. "PAML 4: Phylogenetic Analysis by Maximum Likelihood." *Molecular Biology and Evolution* 24: 1586–1591.
